# Metal Ion Promiscuity and Structure of 2,3‐Dihydroxybenzoic Acid Decarboxylase of *Aspergillus oryzae*

**DOI:** 10.1002/cbic.202000600

**Published:** 2020-11-23

**Authors:** Gerhard Hofer, Xiang Sheng, Simone Braeuer, Stefan E. Payer, Katharina Plasch, Walter Goessler, Kurt Faber, Walter Keller, Fahmi Himo, Silvia M. Glueck

**Affiliations:** ^1^ Institute of Molecular Biosciences BioTechMed Graz University of Graz 8010 Graz Austria; ^2^ Department of Organic Chemistry Arrhenius Laboratory Stockholm University 10691 Stockholm Sweden; ^3^ Department of Chemistry, Analytical Chemistry University of Graz 8010 Graz Austria; ^4^ Department of Chemistry, Organic & Bioorganic Chemistry University of Graz 8010 Graz Austria

**Keywords:** biocatalysis, computational chemistry, enzyme structure, metal-identity, *ortho*-benzoic acid decarboxylase

## Abstract

Broad substrate tolerance and excellent regioselectivity, as well as independence from sensitive cofactors have established benzoic acid decarboxylases from microbial sources as efficient biocatalysts. Robustness under process conditions makes them particularly attractive for preparative‐scale applications. The divalent metal‐dependent enzymes are capable of catalyzing the reversible non‐oxidative (de)carboxylation of a variety of electron‐rich (hetero)aromatic substrates analogously to the chemical Kolbe‐Schmitt reaction. Elemental mass spectrometry supported by crystal structure elucidation and quantum chemical calculations verified the presence of a catalytically relevant Mg^2+^ complexed in the active site of 2,3‐dihydroxybenoic acid decarboxylase from *Aspergillus oryzae* (2,3‐DHBD_*Ao*). This unique example with respect to the nature of the metal is in contrast to mechanistically related decarboxylases, which generally have Zn^2+^ or Mn^2+^ as the catalytically active metal.

## Graphical abstract

**On your metal**: Elucidation of the unique Mg^2+^‐dependency of 2,3‐dihydroxybenzoic acid decarboxylase from *Aspergillus oryzae*, a member of the amidohydroalse superfamily, facilitates a better structure‐activity prediction of the potential biocatalyst, which is capable of catalyzing the regioselective *ortho*‐(de)carboxylation of phenol derivatives.

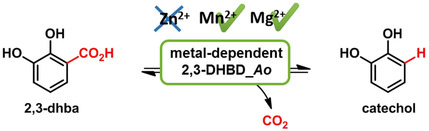

The use of carbon dioxide as a C_1_ reagent for the production of valuable chemicals, such as urea, (poly)carbonates and phenolic acids, has recently sparked growing attention.^[1]^ Of particular interest is the carboxylation of C nucleophiles yielding carboxylic acids.^[2]^ A perfect atom economy of 100 % and the low (no) cost of the reagent CO_2_ renders carboxylation a highly attractive method. However, in traditional chemical protocols harsh reaction conditions require significant energy input, which often causes limited (regio)selectivities.^[3]^ In this context, biocatalytic alternatives, by making use of decarboxylases in the reverse carboxylation direction, offer an elegant alternative.^[4]^ Decarboxylases from secondary metabolic pathways are particularly attractive due to their relaxed substrate portfolio.^[5]^ Three main types of enzymes, which greatly differ in their catalytic mechanism and cofactor requirement and consequently act on completely different substrates, have been elucidated so far:^[4b]^ i) *ortho*‐carboxylation of phenols in analogy to the Kolbe‐Schmitt process^[3]^ is catalyzed by metal‐dependent *o*‐benzoic acid decarboxylases (*o*‐BDs), which excel not only due to their high stability, but also by an unusually broad substrate portfolio.^[6]^ ii) Side‐chain carboxylation at the vinyl group of *p*‐hydroxystyrenes, for which no chemical protocol exists, is feasible with metal‐independent phenolic acid decarboxylases.^[7]^ iii) More recently, the (ATP‐independent) *p*‐carboxylation of phenols and the decarboxylation of electron‐rich heterocyclic and acrylic acid derivatives was shown to be catalyzed by prenylated FMN‐dependent decarboxylases.^[8]^ Although some of these enzymes display a remarkable substrate acceptance, stability‐problems of their prenylated FMN‐cofactor impose limitations on their large‐scale use.^[9]^

Due to their independence from sensitive cofactors and their excellent stability, *o*‐BDs are most attractive for large‐scale applications.[^10^, ^11^] In order to facilitate their applicability, computational methods are increasingly applied for the prediction of substrate‐structure activities aiming to minimize time‐consuming and expensive trial‐and‐error wet‐lab experiments. Although the mechanism of *o*‐BDs is basically well understood,[^12^, ^13^] conflicting data exist concerning the nature of their catalytically essential divalent metal, for example, Zn^2+^, Mn^2+^ or Mg^2+^, which aggravates computational studies leading to inaccuracies in substrate‐binding and energy pathways depending on the ionic radius and Lewis acidity^[14]^ of the metal involved. In this study, we investigated the metal dependence of 2,3‐dihydroxybenzoate decarboxylase from *Aspergillus oryzae* (2,3‐DHBD_*Ao*)^[15]^ by determination of its crystal structure, metal ion analysis by inductively coupled plasma tandem mass spectrometry (ICPMS/MS) and the catalytic energy profile by quantum chemical calculations.

All of the metal‐dependent decarboxylases identified so far are members of the amidohydrolase superfamily, which share significant structural and mechanistic similarities, such as a (β/α)_8_‐barrel fold harboring one catalytically essential divalent metal ion in the active site.^[16]^ Although the overall sequence similarity between subclasses is low, several amino acid residues involved in metal binding and catalysis are conserved (Table 1).^[12]^ The mechanism follows an electrophilic aromatic substitution in analogy to the Kolbe‐Schmitt reaction: First, a divalent metal ion chelates the carboxylate and phenol group in the *o*‐position, which facilitates protonation at the *ipso*‐carboxylate position by a highly conserved Asp residue, thereby breaking aromaticity as the rate‐determining step. Subsequent cleavage of the C−C bond yields CO_2_ and phenol as products (see figure in Table 1).^[4b]^ The identity of the electrophile CO_2_ as co‐product/co‐substrate (as opposed to the nucleophile bicarbonate) has recently been unambiguously clarified.^[17]^

**Table 1:** Simplified general mechanism of *o*‐BDs; catalytically active metal ions and their ligands among *o*‐BDs listed by increased sequence identity to 2,3‐DHBD_*Ao*.

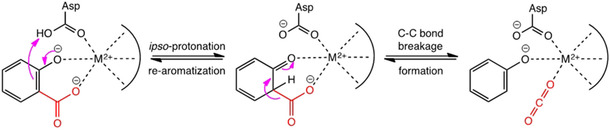						
Decarboxylase	Catalytic Asp	M^2+^ Ligands	M^2+^	Sequence identity	PDB	Ref
IDC_*Cm*	Asp323	His12, His14, His195, Asp323	Mn^2+^; (Zn^2+^)^[a]^	19 %	4HK5	[^12^, ^19^]
LigW_*Sp*	Asp296	Glu7, His173, Asp296	Mn^2+^	26 %	4ICM	[^20^, ^21^]
LigW_*Na*	Asp314	Glu19, His188, Asp314	Mn^2+^	29 %	4QRN	^[20]^
2,6‐DHBD_*Rs*	Asp287	Glu8, His10, His164, Asp287	Zn^2+^	42 %	2DVU	[^22^, ^23^, ^24^]
2,6‐DHBD_*Ps*	Asp287	Glu8, His10, His164, Asp287	Mn^2+^	42 %	4QRO	^[13]^
SAD_*Tm*	Asp298	Glu8, His169, Asp298	Zn^2+^	51 %	6JQW	[^25^, ^26^]
2,3‐DHBD_*Fo*	Asp291	Glu8, His167, Asp291	Zn^2+^	74 %	6M53	^[18]^
2,3‐DHBD_*Ao*	Asp293	Glu8, His167, Asp293	Mg^2+^	100 %	7A19	^[15]^, this study

[a] Previous assumption^[19]^ IDC_Cm=*iso*‐orotate decarboxylase from *C. militaris*;^[19]^ LigW_*Sp* and LigW_*Na*=5‐carboxyvanillate decarboxylase from *Sphingomonas paucimobilis* and *Novosphingobium aromaticivorans*;[^20^, ^21^] 2,6‐DHBD_*Rs* and 2,6‐DHBD_*Ps*=2,6‐dihydroxybenzoic acid decarboxylase^[22]^ from *Rhizobium* and *Polaromonas* species;[^13^, ^23^, ^24^] SAD=salicylic acid decarboxylase from *Trichosporon moniliiforme*;[^25^, ^26^] 2,3‐DHBD_*Fo* and 2,3‐DHBD_*Ao*=2,3‐dihydroxybenzoic acid decarboxylase from *Fusarium oxysporum* and *A. oryzae*.[^15^, ^18^]

*o*‐Benzoic acid decarboxylases (*o*‐BDs) and close relatives display an interesting variety of divalent metal ion requirement. Initially Zn^2+^ was considered as the dominating metal, but recently the picture became more complex (Table 1). Elemental mass spectroscopy (ICPMS/MS) and density functional theory (DFT) calculations identified Mn^2+^ (rather than Zn^2+^ as previously assumed) as essential metal in *iso*‐orotate decarboxylase from *Cordyceps militaris* (IDCase_*Cm*).^[12]^ In accordance, characterization of 2,3‐DHBD_*Ao* revealed that it does not contain Zn^2+^ as reported for a homologous enzyme from *Fusarium* species,^[18]^ but is also catalytically active with Mn^2+^. More surprisingly, 2,3‐DHBD_*Ao* has a significantly improved turnover rate with Mg^2+^. To the best of our knowledge, this is the first example of a Mg^2+^‐dependence of *o*‐BDs. These findings are corroborated by quantum chemical calculations, which revealed a reduced activation barrier by 2 kcal/mol in the rate determining step (see below).

The structure of recombinant 2,3‐dihydroxybenzoic acid decarboxylase of *A. oryzae* was solved by X‐ray crystallography to 1.2 Å (Table S1 in the Supporting Information). In accordance with the recently published homologue from *F. oxysporum* ^[18]^ which shows 74 % sequence identity and related decarboxylases, the enzyme features a distorted (β/α)_8_‐barrel fold with its active site at the centre of the barrel (Figure 1A). The catalytic metal shows well defined density complexed by Glu8, His167, the proton donor Asp293 and three water molecules. Interpretation of this density as the expected Zn^2+^ (Figure 1B) vastly overestimated the electron density. In contrast, Mg^2+^ gave a perfect fit (Figure 1B). Comparison (Zn^2+^ vs Mg^2+^) of the electron density based on the *F. oxysporum* crystal structure (PDB ID: 6M53)^[18]^ reflects our findings (Figure S1). Crystals grown in the absence of Mg^2+^ showed less electron density, indicating a depletion of metal. In this case, the active site was either never correctly reconstituted with the metal during expression, or it was lost during purification and crystallization due to the weak trivalent complexation of the ion. Likewise, lack of the preferred ion, such as Zn^2+^ or Mn^2+^, would be due to their low abundance in growth medium at high protein expression levels.

**Figure 1:**
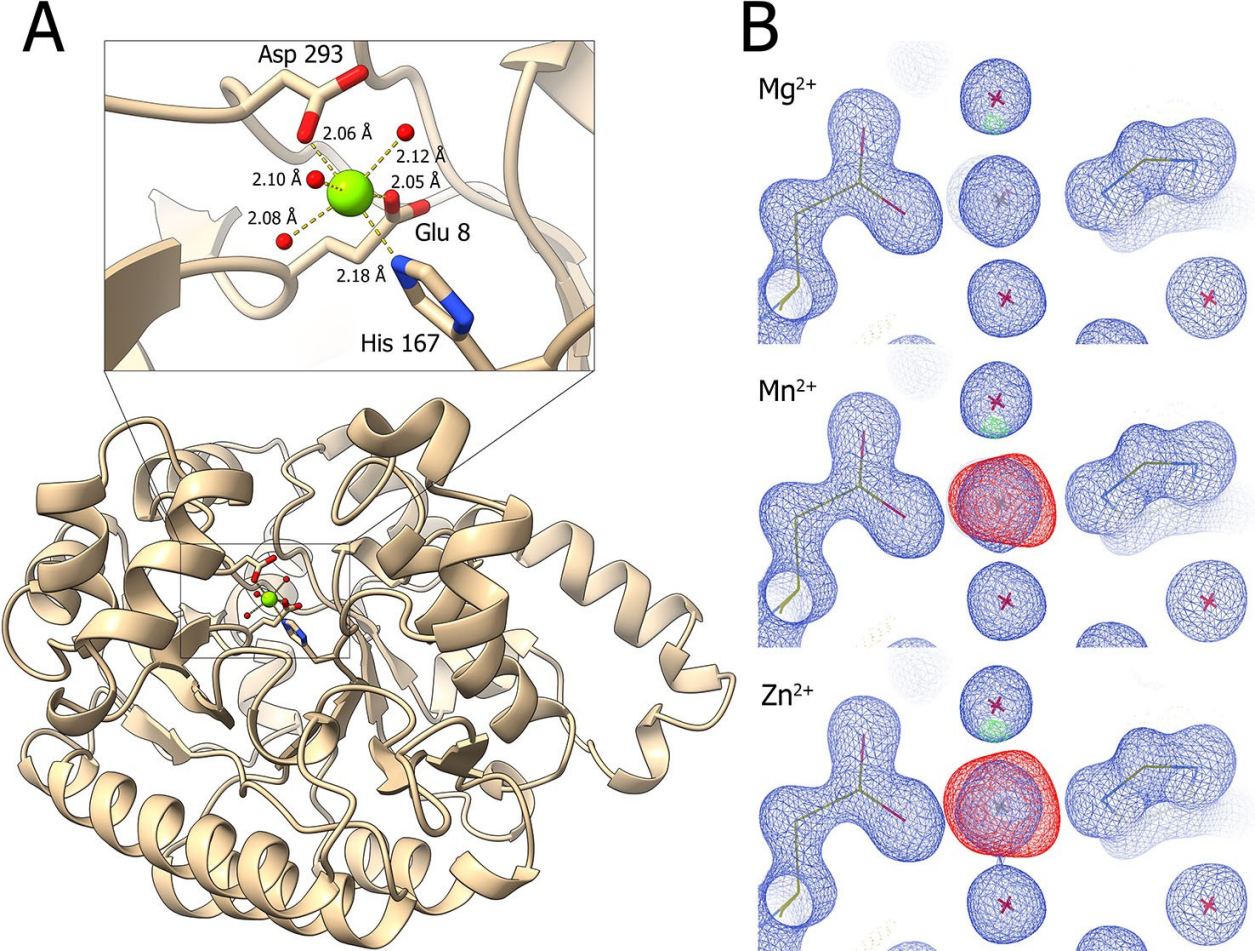
A) Cartoon representation of 2,3‐DHBD_*Ao*; complexing amino acids are shown as sticks, Mg^2+^ ions in green and water molecules in red. B) Electron density map (2 *F*_obs_−*F*_calc_) of models with Mg^2+^, Mn^2+^ or Zn^2+^ in blue contoured at 1.5 root mean square deviation (rmsd), Difference map (*F*_obs_−*F*_calc_) drawn at 5 rmsd shows the excess of model electrons for Mn^2+^ and Zn^2+^ (red). The *B* factors at full occupancy refined to 18.9 for Zn^2+^, 17 for Mn^2+^ and 9 for Mg^2+^ compared to the envelope *B* factor of 12.

To exclude that the occurrence of Mg^2+^ was a crystallographic artefact, ICPMS/MS analysis as well as activity measurements were performed.

Protein samples obtained from size exclusion chromatography (SEC) incubated with either Mg^2+^, Mn^2+^, Zn^2+^ (or a buffer devoid of these ions as control) were split into two parts to determine steady state turnover frequencies (TOF_max_, Figure 2) and their metal occupancy. SEC coupled to ICPMS/MS showed that the control sample contained no Zn^2+^ and only low amounts of Mg^2+^ and Mn^2+^ (around 17 and 10 % occupancy, respectively, Table S2, Figure S2). Incubation with Mg^2+^ or Mn^2+^ showed uptake of either ion with differences in affinity and competition. Mn^2+^ was able to displace the Mg^2+^ originally found and eluted from the SEC column in stoichiometric ratio with the protein. On the other hand, Mg^2+^ was unable to replace Mn^2+^ and was only recovered with 60 % occupancy. This reduced ratio is likely due to a significant off rate of Mg^2+^ from the active site during SEC at 30 °C in Mg^2+^‐free buffer, rather than an inability to completely saturate the enzyme, especially considering the complete occupancy in the crystal. Zn^2+^ caused complete precipitation of the enzyme even in sub‐millimolar concentrations.^[27]^ Enzyme‐bound zinc could not be detected in any of the samples.

**Figure 2:**
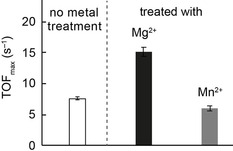
Steady‐state turnover frequency of 2,3‐DHBD_*Ao* treated with either Mn^2+^ or Mg^2+^ in the decarboxylation of 2,3‐dihydroxybenzoic acid (2,3‐dhba).

Additionally, we determined the relative activity of 2,3‐DHBD_*Ao* in the decarboxylation of the enzyme‘s natural substrate 2,3‐dihydroxybenzoic acid (2,3‐dhba)^[15]^ with respect to the metal ion in the active site. The highest rate was found with protein incubated with Mg^2+^, which was approximately 2.5 times higher than the one treated with Mn^2+^ and twice as high as the control sample without any additional metal ions (Figure 2). As mentioned above, the control samples were not completely devoid of Mg^2+^. This explains the slightly higher activity in the non‐metal‐treated preparation versus the Mn^2+^‐treated sample where most of the Mg^2+^ ions were replaced by Mn^2+^. In order to complete the data set, protein samples were also treated with Zn^2+^, which led to complete loss of enzyme activity due to precipitation.^[27]^ Kinetic parameters of 2,3‐DHBD_*Ao* loaded with Mg^2+^ or Mn^2+^ are well within the range of other metal‐depending decarboxylases with different substrate preferences (Table S3). The *k*_cat_/*K*_m_ value for Mg^2+^ containing 2,3‐DHBD and the (putatively) Zn^2+^ occupied enzyme from literature is virtually identical.

Considering that the presence of Mg^2+^ is unprecedented in this enzyme family, we set out to corroborate these findings by quantum chemical calculations.

To gain insights into the reaction mechanism of 2,3‐DHBD_*Ao*, density functional theory (DFT) calculations were performed using a large cluster model of the active site (see the Supporting Information for computational details and model design). It was found that 2,3‐DHBD_*Ao* follows a similar mechanism as previously established for LigW^[17]^ and 2,6‐DHBD,^[13]^ both in terms of the sequence of steps and also the associated energy barriers (Figure 3). For the Mg‐enzyme complex, the protonation of the substrate by Asp293 was found to be rate‐limiting, with a calculated barrier of 15.8 kcal/mol (TS1_Mg_), and the formed 2,4‐dienone intermediate (Int_Mg_) lies 8.8 kcal/mol above the enzyme‐substrate complex (E:S_Mg_). The following decarboxylation was calculated to have a low barrier (TS2_Mg_), 2.9 kcal/mol higher than Int_Mg_. The activation barrier for the Mn‐enzyme complex using the same active site model is calculated to be 2.0 kcal/mol higher than Mg‐enzyme (see the Supporting Information), which agrees well with the experimentally measured trend discussed above. A final mechanistic note worth mentioning here is that the monodentate substrate binding mode, that is, with only the carboxylate group of 2,3‐dihydroxybenzonate being coordinated to the metal, is less favored than the bidentate binding shown in Figure 3 (see also the Supporting Information).

**Figure 3:**
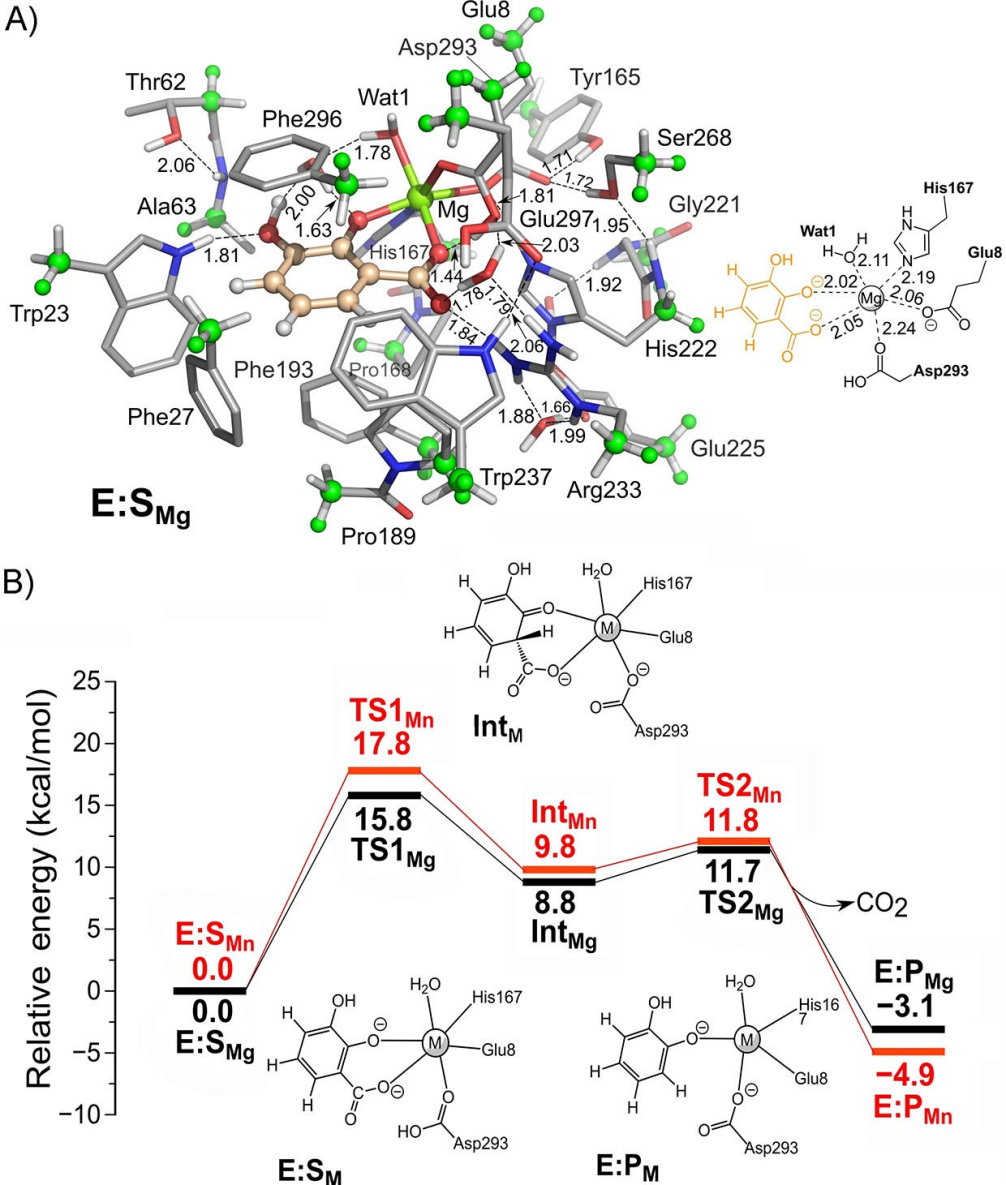
a) Optimized structure of the model of the enzyme–substrate complex for the Mg‐dependent enzyme obtained by the quantum chemical model calculations, and b) the calculated energy profiles for the reactions of Mg‐ and Mn‐dependent enzymes.

To summarize, crystal structure elucidation and metal analysis by ICPMS/MS supported by mechanistic quantum chemical calculations have identified Mg^2+^ as the catalytically relevant metal in the active site of 2,3‐dihydroxybenzoic acid decarboxylase from *A. oryzae*. This finding represents a unique example within metal‐dependent *o*‐benzoic acid decarboxylases, which generally depend on Zn^2+^ or Mn^2+^, and is essential for future structure‐activity predictions.

## Conflict of interest

The authors declare no conflict of interest.

## Supporting information

As a service to our authors and readers, this journal provides supporting information supplied by the authors. Such materials are peer reviewed and may be re‐organized for online delivery, but are not copy‐edited or typeset. Technical support issues arising from supporting information (other than missing files) should be addressed to the authors.

Supplementary
