# Supplementary material for: Metal Ion Promiscuity and Structure of 2,3‐Dihydroxybenzoic Acid Decarboxylase of Aspergillus oryzae
Source: Chembiochem. 2020 Nov 23;22(4):652–6. doi: 10.1002/cbic.202000600 (PMC7894528; doi:10.1002/cbic.202000600)
Supplement: Supplementary file 1 — Supplementary [file CBIC-22-652-s001.pdf]

ChemBioChem

Supporting Information

Metal Ion Promiscuity and Structure of 2,3-Dihydroxybenzoic Acid Decarboxylase of *Aspergillus oryzae*

Gerhard Hofer[+], Xiang Sheng[+], Simone Braeuer, Stefan E. Payer, Katharina Plasch, Walter Goessler, Kurt Faber, Walter Keller, Fahmi Himo,* and Silvia M. Glueck*

Supporting Information

Table of Contents

Refinement statistics S2
Expression and purification S3
Crystallization and structure determination S4
Incubation with metal ions S4
Elemental mass spectrometry S4
Determination of the decarboxylation activity S7
Computational methods S11
Geometries of transition states and intermediates of the Mg-dependent enzyme S13
Geometries of transition states and intermediates of the Mn-dependent enzyme S15
Alternative monodentate binding mode of the substrate S17
Cartesian coordinates S19
References S46

Table S1. Refinement statistics of PDB:7A19 and PDB:7A1A

	7A19 (Mg condition)	7A1A (Ca condition)
**Wavelength [Å]**	1.0332	1.0332
**Resolution range**	47.64 - 1.21 (1.254 - 1.21)	49.95 - 1.53 (1.584 - 1.53)
**Space group**	P 43 21 2	P 43 21 2
**Unit cell**	99.794 99.794 129.177 90 90 90	99.895 99.895 129.168 90 90 90
**Total reflections**	696751 (3510)	2342821 (239201)
**Unique reflections**	165014 (2990)	98740 (9747)
**Multiplicity**	4.2 (1.2)	23.7 (24.5)
**Completeness (%)**	83.12 (15.34)	99.64 (99.08)
**Mean I/sigma(I)**	6.99 (1.07)	8.94 (1.82)
**Wilson B-factor**	11.53	15.44
**R-merge**	0.1235 (0.3631)	0.2276 (0.8963)
**CC1/2**	0.986 (0.764)	0.986 (0.959)
**Reflections used in refinement**	163886 (2990)	98457 (9680)
**Reflections used for R-free**	8099 (138)	4845 (474)
**R-work**	0.1411 (0.2525)	0.1920 (0.2378)
**R-free**	0.1717 (0.2994)	0.2246 (0.2738)
**Number of non-hydrogen atoms**	6763	6258
**macromolecules**	5573	5540
**ligands**	3	3
**solvent**	1187	715
**Protein residues**	678	678
**RMS(bonds)**	0.007	0.007
**RMS(angles)**	0.99	0.85
**Ramachandran favored (%)**	95.1	95.55
**Ramachandran allowed (%)**	4.6	4.15
**Ramachandran outliers (%)**	0.3	0.3
**Rotamer outliers (%)**	0.34	0
**Average B-factor**	15.11	18.52
**macromolecules**	12.79	17.61
**Mg^{2+}, Ca^{2+}**	15.49	19.42
**Solvent**	26.03	25.56

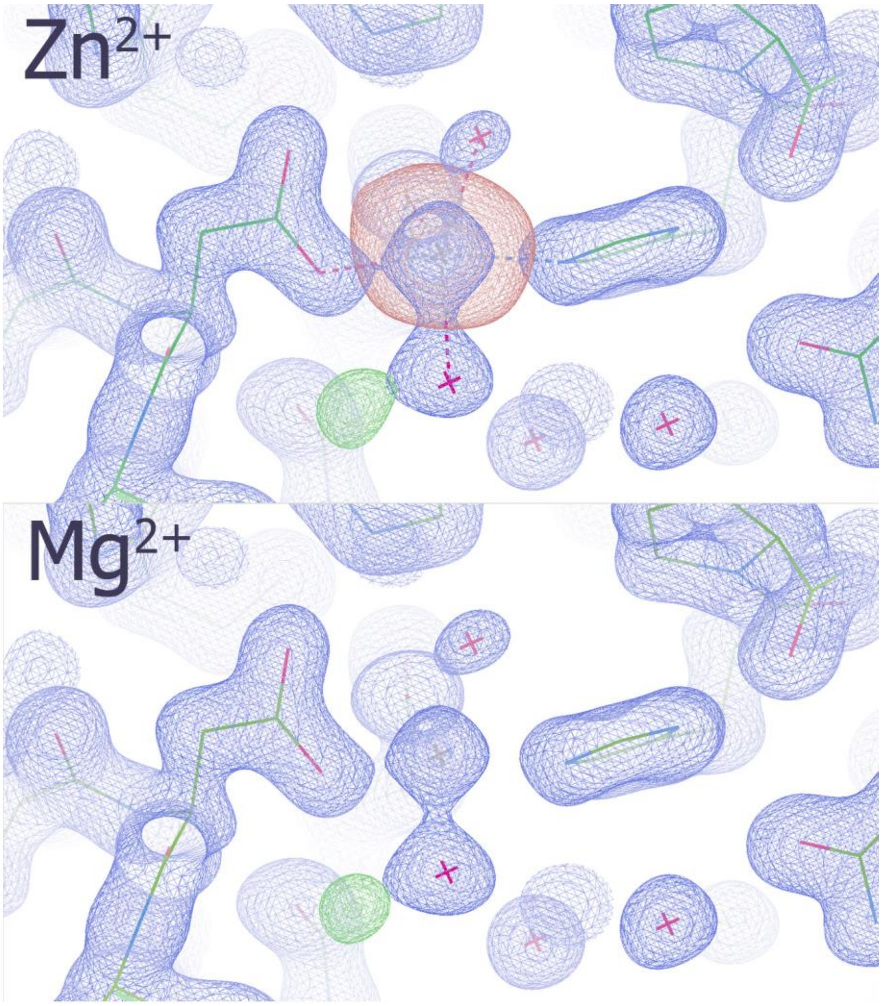

Figure S1. Electron density map ($2F_{obs}$-F_{calc}) of 2,3-DHBD_*Fo* (PDB ID 6M53) as deposited with Zn^{2+} or Mg^{2+}, respectively, contoured at 1.5 root mean square deviation (r.m.s.d.) (blue). Difference map (F_{obs}-F_{calc}) drawn at 5 r.m.s.d shows the excess of model electrons for Mn^{2+} and Zn^{2+} (red/green). Both models were refined by PDB redo.[1] The B factors in chain A at full occupancy refined to 42.3 for Zn^{2+} and to 28.7 for Mg^{2+} compared to the envelope B factor of 27.6 and 25.9, respectively.

Expression and purification

The synthetic gene for 2,3-DHBD_*Ao* (accession number XP_001817513) was cloned into pET29a(+) using NdeI/Xho1. *E. coli* BL21-DE3 cultures transfected with this plasmid were grown to an OD600 of 0.6 in LB medium at 37 °C before being induced with 1 mM IPTG and left to shake at 180 rpm over night at 20 °C. Pellets harvested by centrifugation were lysed by sonication and purified by Ni NTA purification as described previously.[2] Pooled and concentrated fractions of the Ni purification were polished by preparative SEC on a Superdex 200 Increase 10/300 column (Cytiva) with 150 mM NaCl 10 mM MES pH 6.5 as the mobile

phase. The single peak corresponding to the tetramer was pooled and used for further experiments.

Crystallization and structure determination

The purified protein was crystallized at 30 mg/mL from a 50 mM Tris pH 7.5 buffer in sitting drop setups mixed with equal volumes 0.1 M magnesium chloride 0.1 M Na HEPES 7.5 and 10% w/v PEG 4000 for the condition fully saturated by magnesium and 0.1 M calcium acetate 0.1 M Na acetate 4.5 and 10% w/v PEG 4000 for the crystals with partially empty ion binding site. Diffraction was collected at beamline P11 at PETRA III at 1.0332 Å.[3] No anomalous signal was found and therefore Friedel equivalent reflections were merged during data reduction. Phases were obtained by molecular replacement using the structure of 2,6-DHBD (PDB code 2DVT). The structures were refined using the phenix suite[4] with iterative model building in coot.[5] The coordinates and diffraction data were deposited in the PDB as etries 7A19 and 7A1A. For the refinement statistics see table S1.

Incubation with metal ions

Decarboxylase samples devoid of Mg^{2+} and Mn^{2+} ions obtained from SEC were mixed 1+1 with solutions of $MgSO_4$ and $MnSO_4$ in SEC elution buffer (50 mM each, >1000 times molar excess), respectively, while the control sample was diluted 1+1 with non-modified elution buffer. All samples were incubated without shaking at 37 °C overnight and triplicates for each treatment were performed. For activity measurements the protein concentration in the individual samples was adjusted to 0.1 – 0.8 mg/mL by dilution with potassium phosphate buffer (100 mM, pH 5.5, i.e. reaction buffer).

Elemental mass spectrometry

Element concentrations and distributions (bound to the enzyme vs. free) were determined by high performance liquid chromatography (HPLC, 1200, Agilent Technologies, Waldbronn, Germany) coupled to inductively coupled plasma tandem mass spectrometry (ICPMS/MS, 8800, Agilent Technologies, Waldbronn, Germany) as described elsewhere.[6] The SEC column Yarra SEC-X150 (150 x 4.6 mm, 1.8 µm, Phenomenex, Aschaffenburg, Germany) was used as stationary phase. The mobile phase consisted of 40 mM $NH_4H_2PO_4$ (99.99%, Suprapur®, Merck KGaA, Darmstadt, Germany) and 20 mM NaCl (≥99.5%, p.a., Carl Roth, Karlsruhe, Germany) at pH 6.5 (adjusted with ammonia solution, 25%, Suprapur®, Merck KGaA). The flow rate was 0.5 mL/min, the temperature 30 °C and the injection volume 10 µL.

ICPMS/MS was operated in oxygen reaction mode (30% cell gas), to improve the detection limit for sulfur. Because of the high salt concentration of the mobile phase, high matrix introduction (HMI) was used ("HMI-4", 0.65 L/min nebulizer gas and 0.34 L/min dilution gas, 10 mm sampling depth). The following m/z ratios were investigated: 24 → 24 (Mg), 32 → 48 (S), 55 → 55 (Mn), 66 → 66 (Zn). Each sample was analyzed three times. $MnCl_2 * 4 H_2O$ (Merck KGaA), $MgCl_2 * 6 H_2O$ (Carl Roth) and sulfate [$(NH_4)_2SO_4$ in H_2O, IC-Standard-Solution Roti®Star, Carl Roth] were used for external calibration (each: 0.05 – 50 mg/L, referring to the element). The 5 mg/L standard was measured after every 10th sample to monitor the stability of the measurement. The column recovery was controlled with flow injections of the samples. The element concentrations were converted to molarities, and the S molar concentrations were converted to molar protein concentration by dividing through 13 (13 S atoms per protein). The occupancies were determined by comparing the molarities of Mg^{2+}, Mn^{2+} and protein (Table S2).

Table S2. Concentrations (in µM and in % occupancy) of bound Mg^{2+} and Mn^{2+} in the different samples (in reference to the protein concentration).

**Sample**	**Mg^{2+} [µM]**	**Mn^{2+} [µM]**	**Mg^{2+} [% occupancy]**	**Mn^{2+} [% occupancy]**
control	3.8 ± 0.1	2.1 ± 0.3	17 ± 1.0	9.7 ±1.5
treated with Mg^{2+}	10 ± 2.0	1.8 ± 0.5	61 ± 5.0	11 ± 2.0
treated with Mn^{2+}	0.74 ± 0.22	18 ± 2.0	4.4 ± 1.3	109 ±13

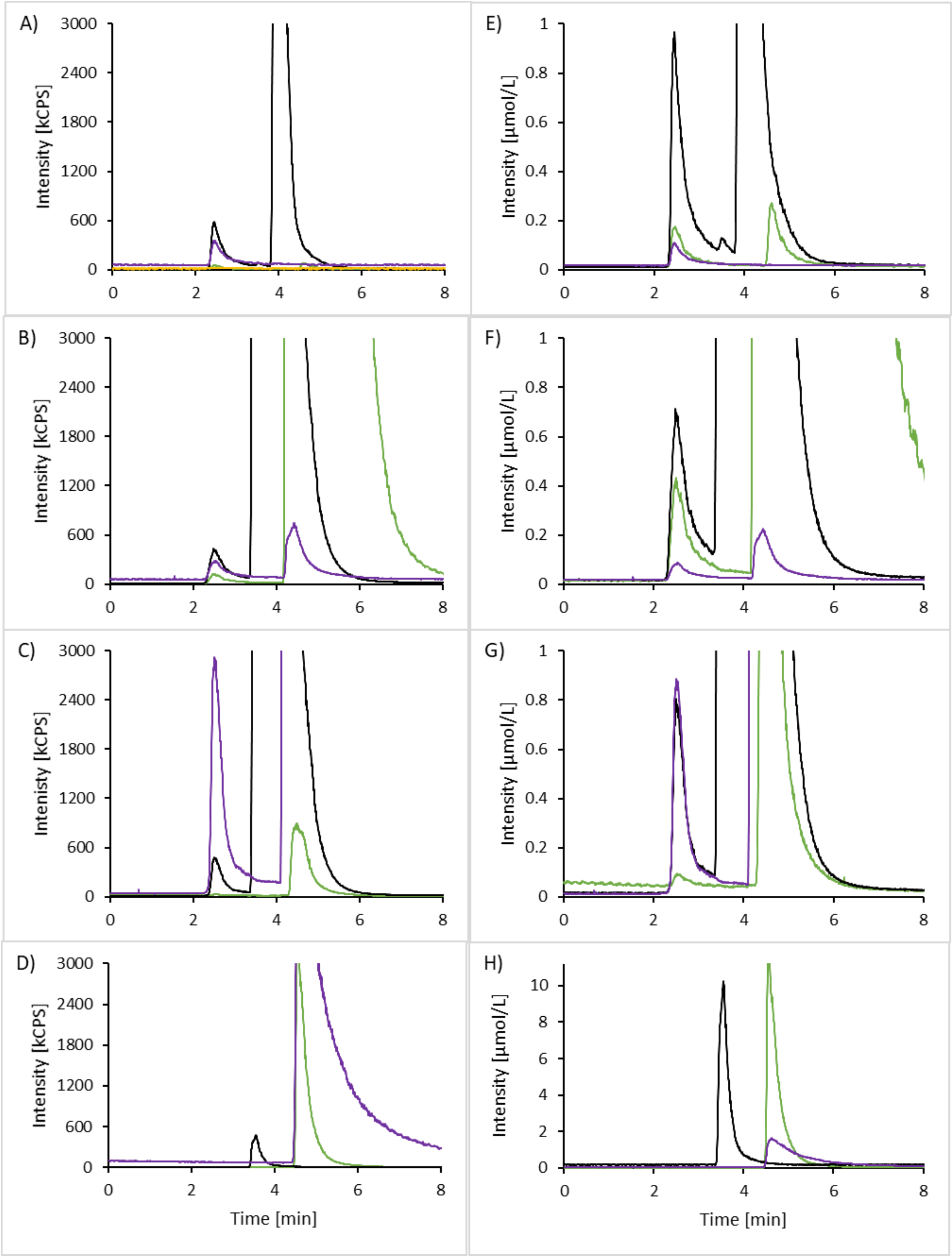

Figure S2. SEC-ICPMS/MS chromatograms of Mg (green), Mn (purple) and S* (black) in the protein samples. The Zn trace (yellow) is shown for the control sample (panel A) only, because it caused precipitation of the protein upon incubation. The protein peak appears at 2.5 minutes,

while the latter S peak is from free sulfate. Left: raw chromatograms in kCPS. Right: Normalized via the external calibrations to µmol/L. A and E: control sample, B and F: treated with Mg^{2+}, C and G: treated with Mn^{2+}. D and H: standard solution with 5 mg/L of S, Mg and Mn. *in E-G, the S-trace is normalized to the protein (14 S per protein).

Determination of the decarboxylation activity

Decarboxylation activity measurements of reconstituted decarboxylases were performed on a SpectraMax M2 plate reader (Molecular Devices) in clear 96 well plates (Greiner) by photometrical monitoring of the substrate decay. Assay mixtures contained substrate 2,3-dihydroxybenzoic acid (2,3-dhba, 2 mM, 99%, Sigma Aldrich) in potassium phosphate buffer (100 mM, pH 5.5) and 2,3-DHBD solution samples. Decarboxylation reactions were started by addition of substrate solution (100 µL/well) to protein sample aliquots (2 µL). After initial mixing for 5 sec, substrate consumption from initial concentrations between 0.1 and 1.2 mM was monitored over 10 min in 10 sec intervals at 300 nm. Turnover frequencies (TOF) in s^{-1} were calculated using the molar extinction coefficient for 2,3-dhba obtained from a calibration curve (ε_{300} = 2800 M^{-1} cm^{-1}) and were normalized to the amount of protein per well and a molar mass of 2,3-DHBD_*Ao* of M = 38857 g/mol (ExPASy Protparam tool).[7] Steady-state TOF_{max} values (s^{-1}) were derived from a rectangular hyperbola fit of 20 – 25 datapoints in a TOF vs. substrate concentration plot (Spectra Max software).

Plate and group control samples were used for each enzyme preparation and v_{max} values (mAU/min) were corrected after data reduction. Path check using a blank cuvette was enabled and the obtained rates were normalized to the individual pathlengths. The absence of spontaneous substrate decay was confirmed in the absence of enzyme. No measurable activity was observed with 2,6-dihydroxybenzoic acid in the same experimental setting.

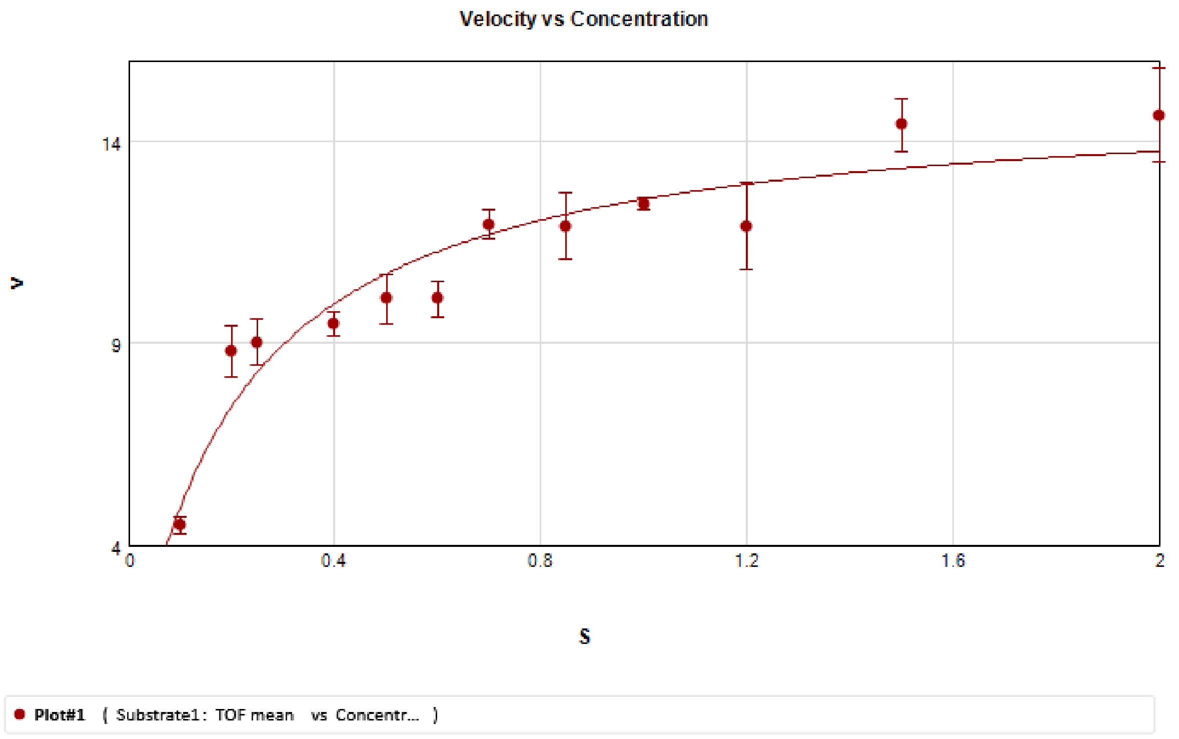

Curve Fit Results ▲

Curve Fit : Rectangular Hyperbola $y = \frac{Ax}{x+B}$

	Parameter	Estimated Value	Std. Error	Confidence Interval	Independence
Plot#1 R^2 = 0.907	A	15.18	0.734	[13.54, 16.81]	
	B	0.208	0.039	[0.120, 0.296]	

Figure S3. Michaelis-Menten fit for 2,3-DHBD_*Ao* sample treated with Mg^{2+}. "S" refers to substrate concentration in mM and "v" to the measured decarboxylation velocity in s^{-1}.

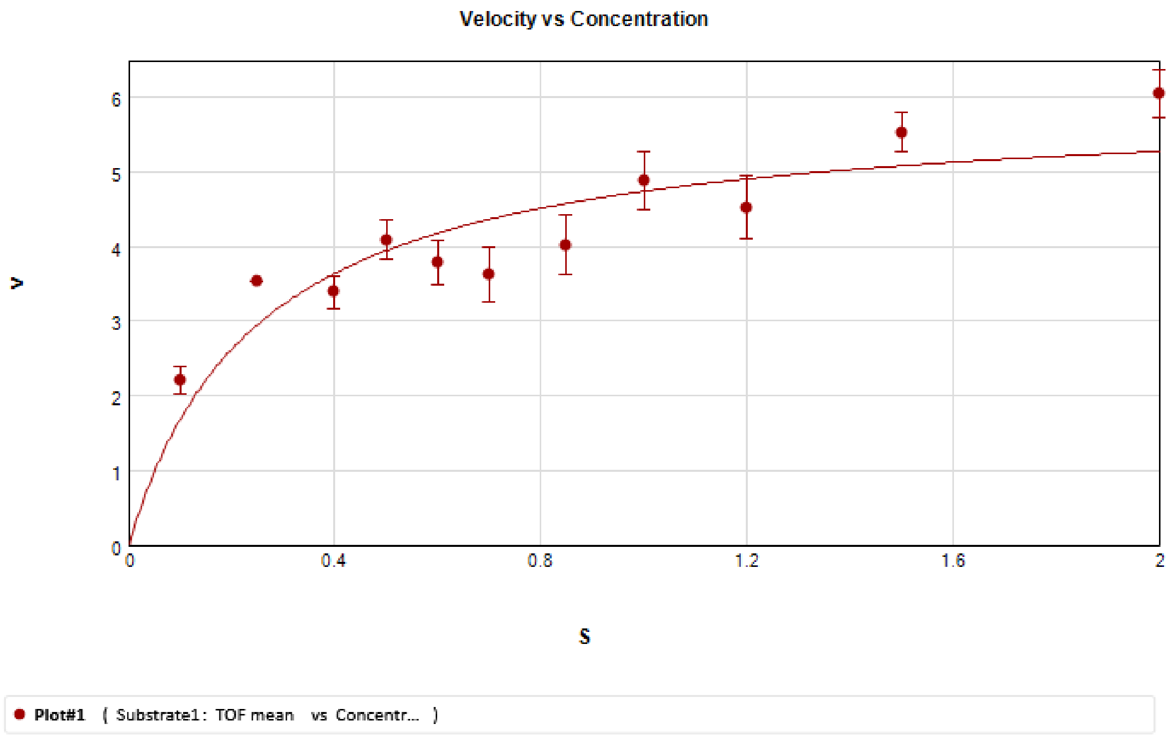

Curve Fit Results ▲

Curve Fit : Rectangular Hyperbola $y = \frac{Ax}{x + B}$

	Parameter	Estimated Value	Std. Error	Confidence Interval	Independence
Plot#1 R^2 = 0.764	A	5.943	0.536	[4.731, 7.156]	
	B	0.253	0.086	[0.058, 0.447]	

Figure S4. Michaelis-Menten fit for 2,3-DHBD_*Ao* sample treated with Mn^{2+}. “S” refers to substrate concentration in mM and “v” to the measured decarboxylation velocity in s^{-1}.

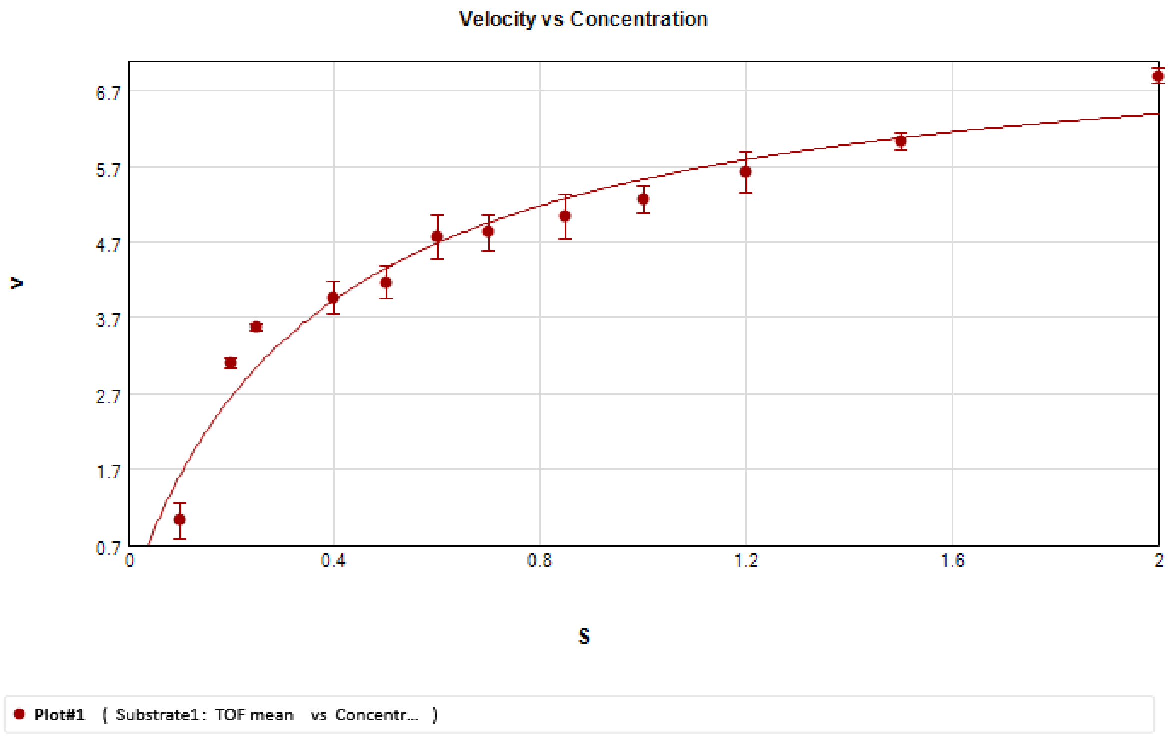

Curve Fit Results ▲

Curve Fit : Rectangular Hyperbola $y = \frac{Ax}{x + B}$

	Parameter	Estimated Value	Std. Error	Confidence Interval	Independence
Plot#1 R^2 = 0.950	A	7.576	0.418	[6.644, 8.508]	
	B	0.370	0.061	[0.234, 0.506]	

Figure S5. Michaelis-Menten fit for non-treated 2,3-DHBD_*Ao*. "S" refers to substrate concentration in mM and "v" to the measured decarboxylation velocity in s^{-1}.

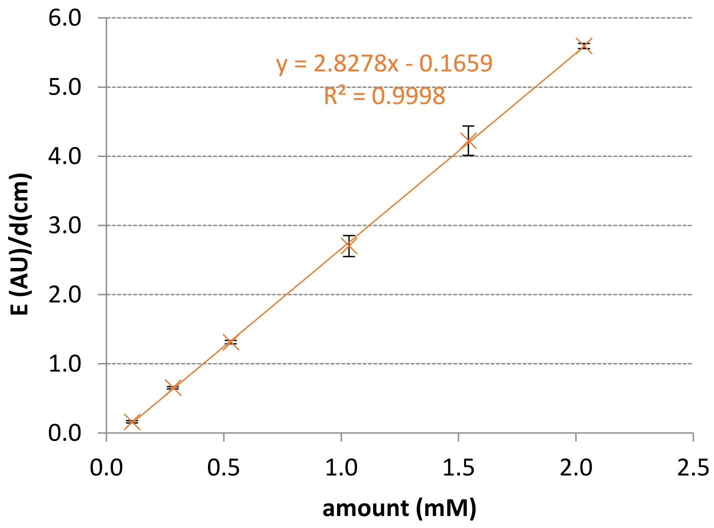

Figure S6. Plate-reader calibration curve for 2,3-DHBD at 300 nm.

Table S3. Kinetic parameters of 2,3-DHBD_*Ao* and other *o*-decarboxylases (from lit.).

Decarboxylase	M^{2+}	Substrate	K_m (µM)	k_{cat} (s^{-1})	$k_{cat}\ K_m^{-1} \times 10^3$	Ref
IDC_*Cm*	Mn^{2+}	*iso*-orotate	22	0.07	3.2	[8]
LigW_*Sp*	Mn^{2+}	5-carboxyvanillate	55	2.2	40.0	[9]
LigW_*Na*	Mn^{2+}	5-carboxyvanillate	245	27	110	[9]
2,6-DHBD_*Rs*	Zn^{2+}	2,6-dhba	71	0.95	13.0	[10]
2,6-DHBD_*Ps*	Mn^{2+}	2,6-dhba	111	0.47	4.2	[11]
SAD_*Tm*	Zn^{2+}	salicylic acid	1080	0.34	0.3	[12]
2,3-DHBD_*Fo*	Zn^{2+}	2,3-dhba	8190	27	3.3	[13]
2,3-DHBD_*Ao*	Zn^{2+}	2,3-dhba	420	3[a]	7.1	[14]
	**Mg^{2+}**	**2,3-dhba**	**208**	**15**	**7.2**	**this study**
	**Mn^{2+}**	**2,3-dhba**	**253**	**6**	**2.4**	**this study**

[a] k_{cat} was calculated from v_{max} = 4.8 µmol min^{-1} mg^{-1} assuming a molecular mass of M = 38857 g mol^{-1} for 2,3-DHBD_*Ao*; 2,3-dhba = 2,3-dihydroxybenzoic acid.

Computational methods

All calculations were performed using the B3LYP-D3(BJ) method[15] implemented in the Gaussian 09 program.[16] Geometry optimizations were carried out with LANL2DZ[17] pseudopotential for Mg and Mn, and the 6-31G(d,p) basis set for the other atoms. To obtain more accurate energies, single-point calculations on the optimized structures were performed with the larger basis set 6-311+G(2d,2p) for all the atoms, except Mg and Mn, which were treated using the same pseudopotential as in the geometry optimization. At the same level of theory as the geometry optimization, the effects of the rest of the enzyme was described using the SMD solvation model[18] with the dielectric constant $\varepsilon = 4$. Zero-point energies (ZPE) were obtained from frequency calculations. Thus, the reported energies are those for the large basis set (which include dispersion) corrected for solvation and ZPE effects. The entropy gain from releasing of CO_2 from the active site was estimated to be the translational entropy of the free molecule, which is 11.1 kcal/mol at room temperature.[19] This value is added to the energy of the CO_2 formation step.

An active site model was designed on the basis of the crystal structure reported in the present study. The 2,3-dihydroxybenzonate substrate was added to the model manually. The model consists of the metal ion along with its ligands (Glu8, His167, Asp293, Wat1, and 2,3-dihydroxybenzonate) and other residues composing of the active site (Trp23, Phe27, Thr62, Ala63, Tyr165, Pro168, Pro189, Phe193, Gly221, His222, Glu225, Arg233, Trp237, Ser268,

Phe296, Glu297). In addition, four crystallographic water molecules were also included in the model. Amino acids were truncated as shown in the figures, and hydrogen atoms were added manually. In the geometry optimizations, a number of atoms were fixed at their crystallographic positions to keep the structure of the active site pocket. These are indicated in green color in the figures below. The model consists of 306 atoms and has a total charge of 0.

Geometries of transition states and intermediates of the Mg-dependent enzyme

Optimized structures for the transition states (**TS1$_{Mg}$** and **TS2$_{Mg}$**) and intermediates (**Int$_{Mg}$** and **E:P$_{Mg}$**) in the reaction pathway of the Mg-enzyme of 2,3-DHBD_*Ao*. The energies relative to the **E:S$_{Mg}$** are given in the parentheses (kcal/mol).

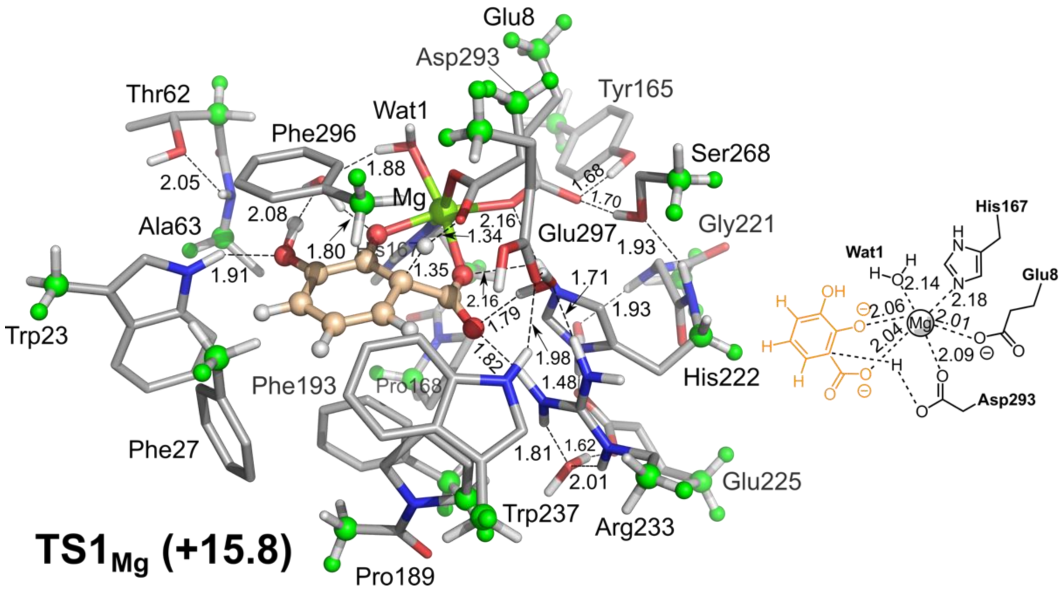

Figure S7. Optimized transition state for the protonation of substrate for the Mg-enzyme. The energy relative to **E:S$_{Mg}$** is given in kcal/mol.

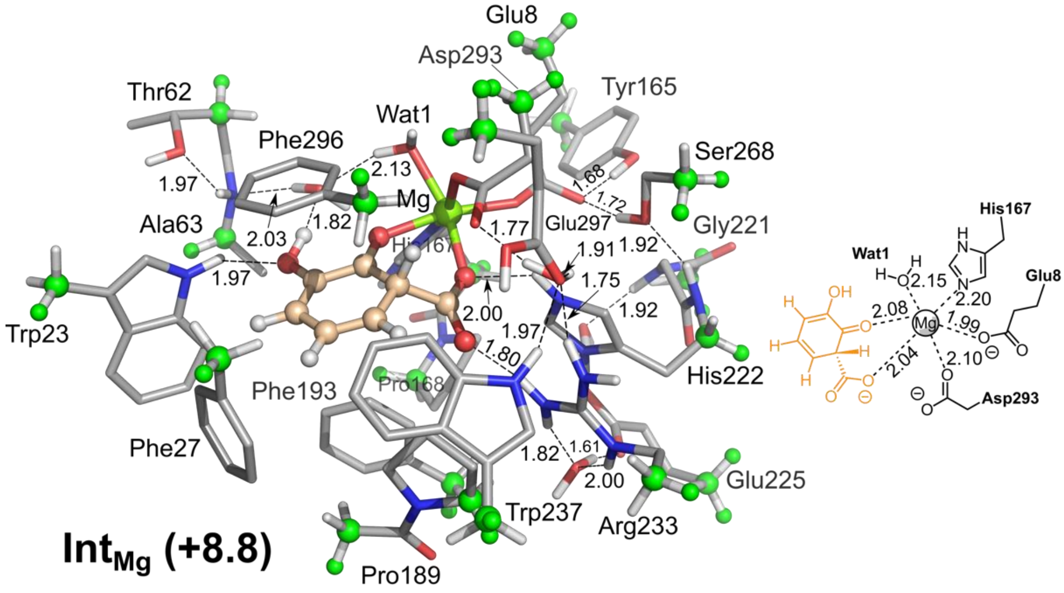

Figure S8. Optimized structure of the 2,4-dienone intermediate for the Mg-enzyme. The energy relative to **E:S$_{Mg}$** is given in kcal/mol.

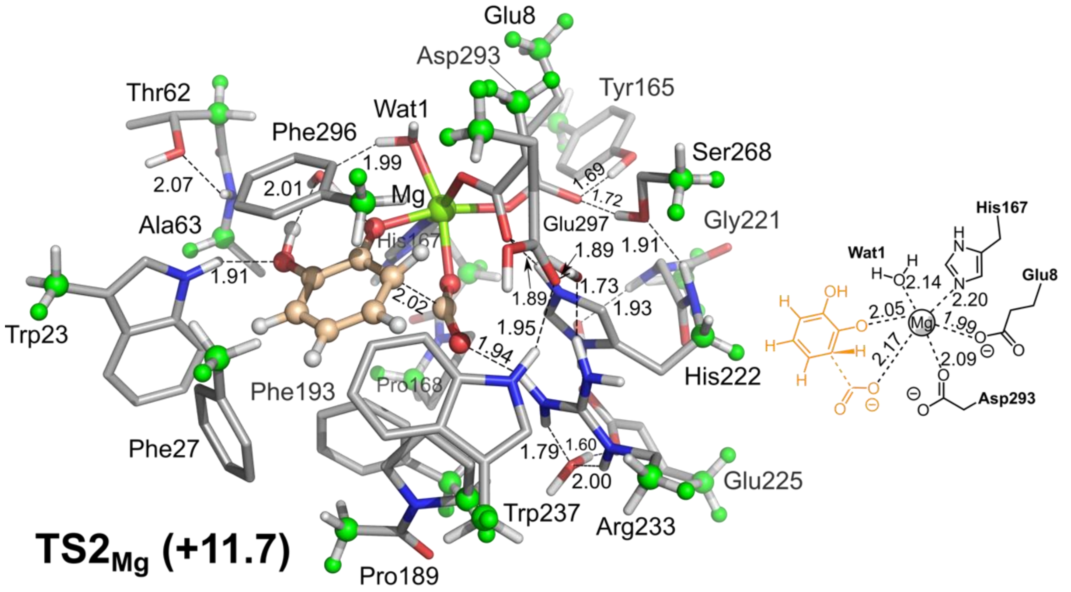

Figure S9. Optimized transition state for the decarbxoyaltion step for the Mg-enzyme. The energy relative to **E:S$_{Mg}$** is given in kcal/mol.

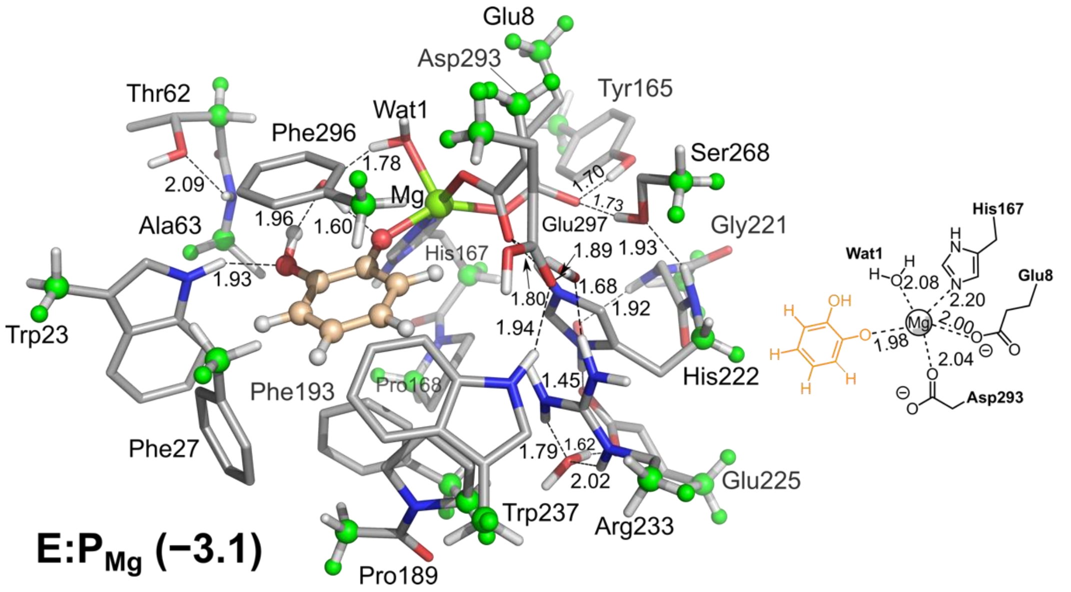

Figure S10. Optimized structure of the enzyme-product complex for the Mg-enzyme. The energy relative to **E:S$_{Mg}$** is given in kcal/mol.

Geometries of transition states and intermediates of the Mn-dependent enzyme

Optimized structures for the enzyme-substrate complex (**E:S$_{Mn}$**), transition states (**TS1$_{Mn}$** and **TS2$_{Mn}$**) and intermediates (**Int$_{Mn}$** and **E:P$_{Mn}$**) in the reaction pathway of the Mn-enzyme of 2,3-DHBD_*Ao*. The energies relative to the **E:S$_{Mn}$** are given in the parentheses (kcal/mol).

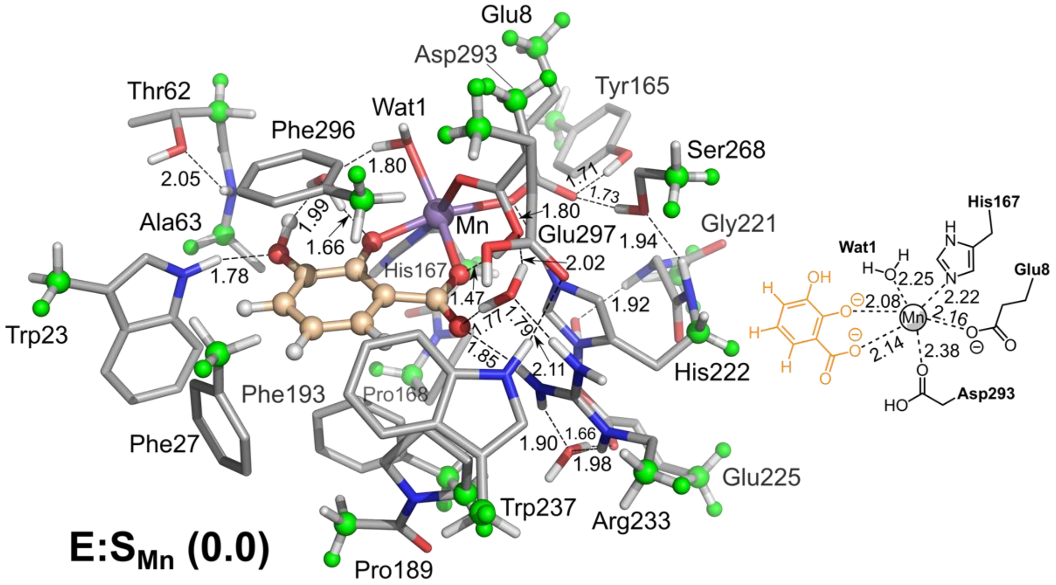

Figure S11. Optimized structure of the enzyme-substrate complex for the Mn-enzyme.

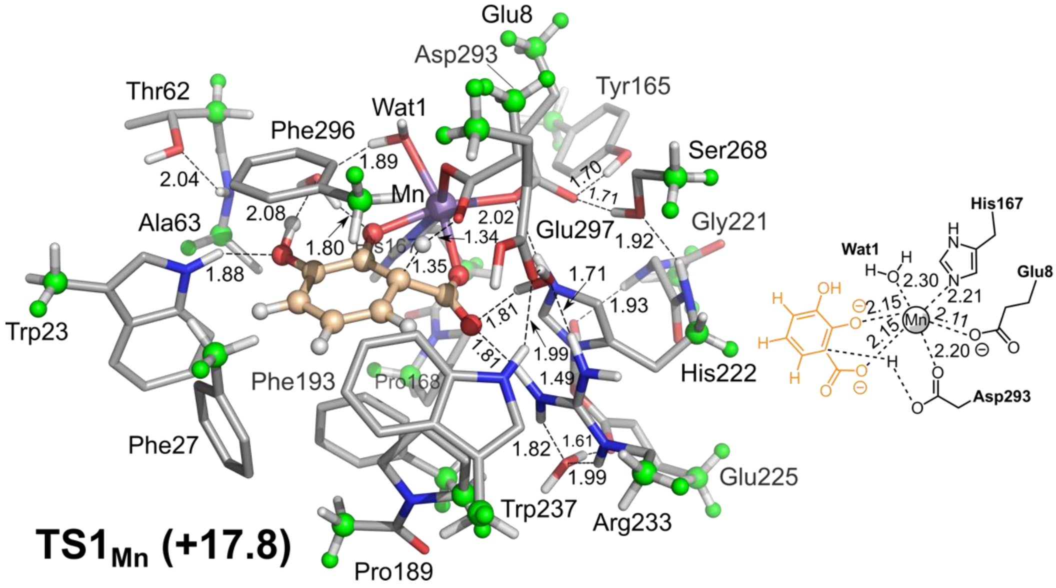

Figure S12. Optimized transition state for the protonation of substrate for the Mn-enzyme. The energy relative to **E:S$_{Mn}$** is given in kcal/mol.

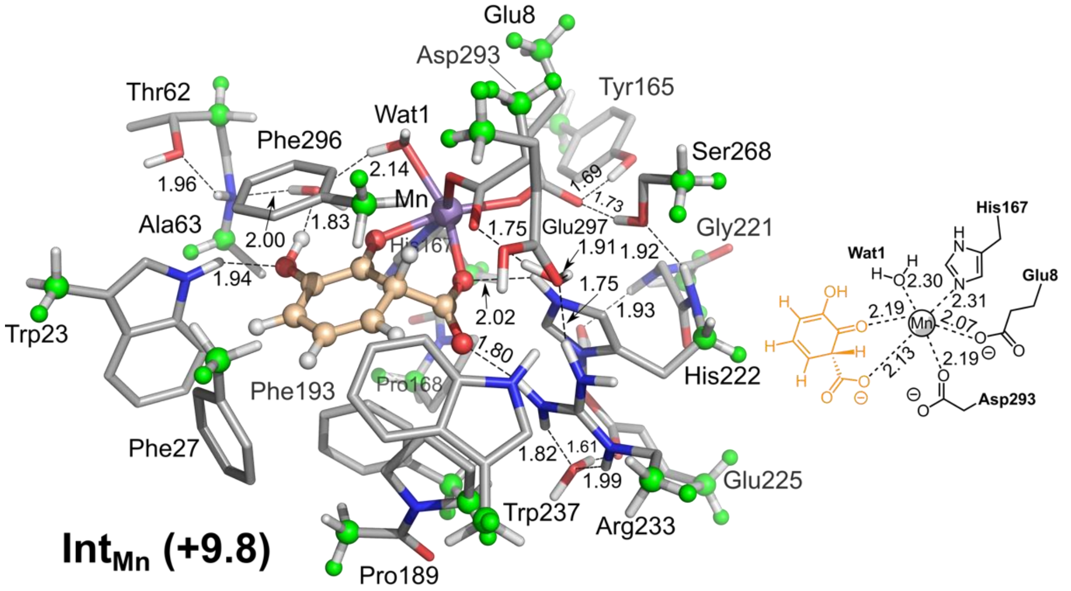

Figure S13. Optimized structure of the 2,4-dienone intermediate for the Mn-enzyme. The energy relative to **E:S$_{Mn}$** is given in kcal/mol.

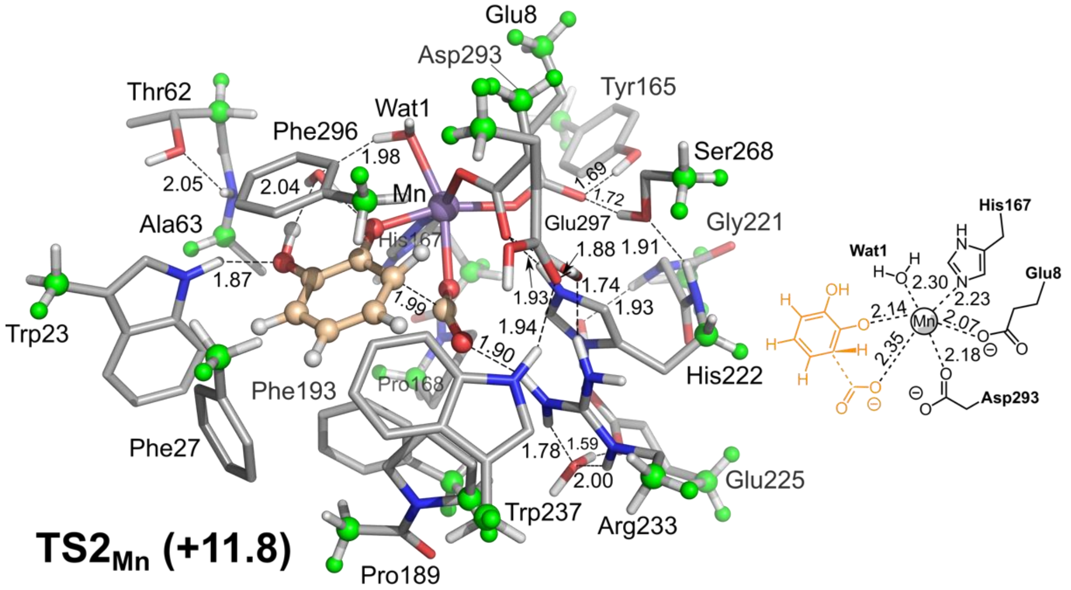

Figure S14. Optimized transition state for the decarbxoyaltion step for the Mn-enzyme. The energy relative to **E:S$_{Mn}$** is given in kcal/mol.

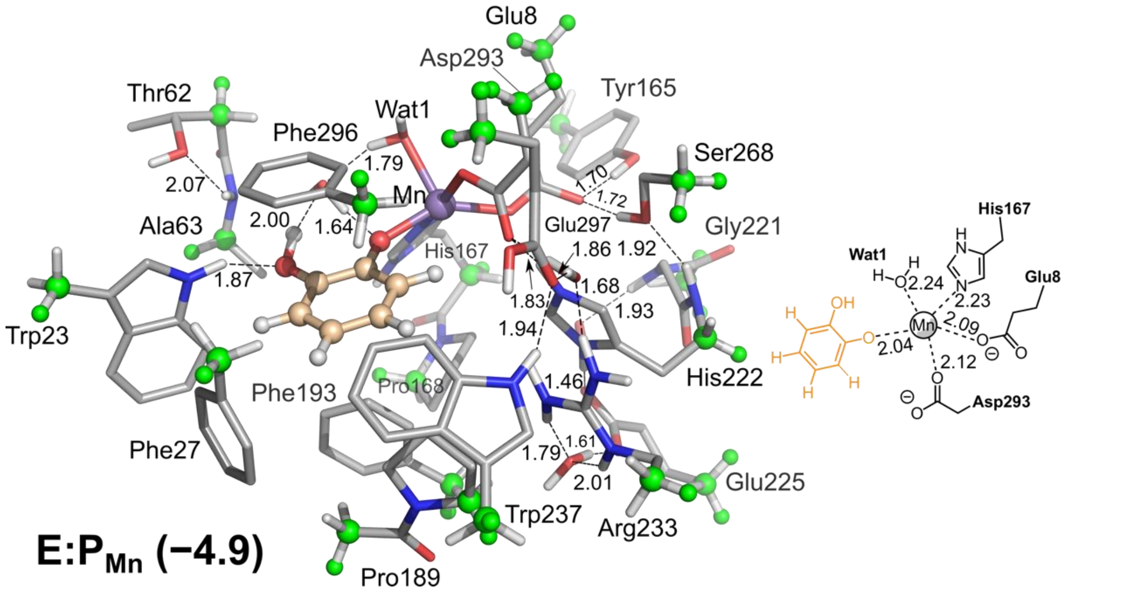

Figure S15. Optimized structure of the enzyme-product complex for the Mn-enzyme. The energy relative to **E:S$_{Mn}$** is given in kcal/mol.

Alternative monodentate binding mode of the substrate

We examined in the present study the binding mode of the substrate in 2,3-DHBD_*Ao*. In a previous work on the Mn-containing 2,6-DHBD_*Ps (γ*-RSD_*Ps)*, two binding modes were considered for the γ-resorcylate substrate, one of which is similar to the bidentate binding of 2,3-dihydroxybenzonate to the metal in 2,3-DHBD_*Ao* as shown in Figure 3 in the paper. The other one is an unproductive monodentate binding mode with only the carboxylate group being coordinated to the metal. In the case of 2,6-DHBD_*Ps*, these two binding modes were found to be comparable in energy, only 0.4 kcal/mol in favor of the monodentate mode. As 2,3-DHBD_*Ao* and 2,6-DHBD_*Ps* belong to the same family, we considered here also the monodentate binding mode for 2,3-DHBD_*Ao*. In this case, the monodentate mode is less favored, with calculated energies of 2.8 kcal/mol and 1.8 kcal/mol higher than the bidentate one for Mg- and Mn-enzymes, respectively (see Figures S16 and S17).

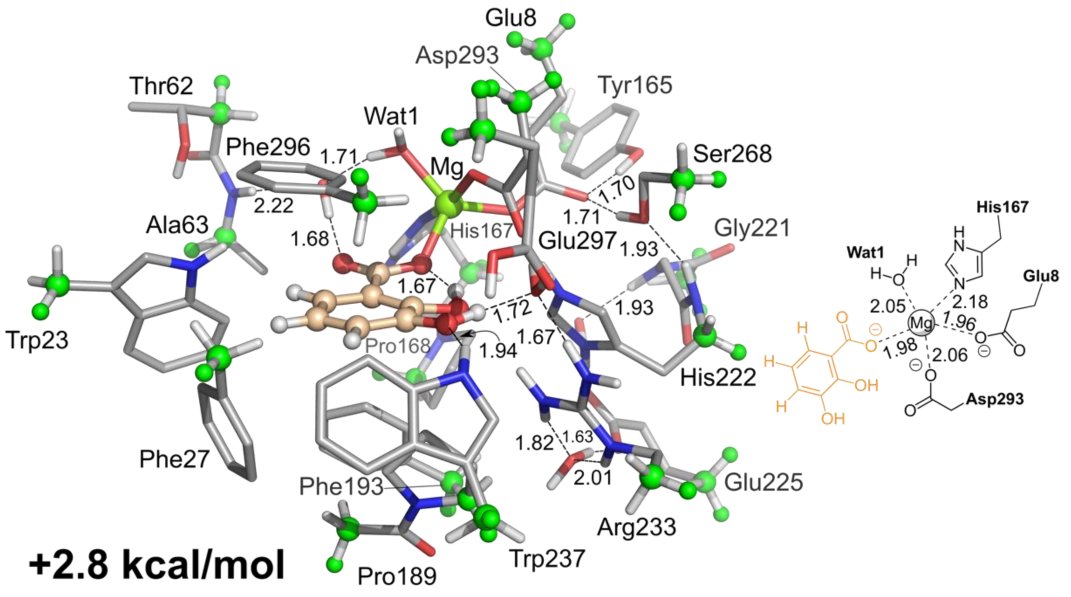

Figure S16. Optimized structure of enzyme-substrate complex with a monodentate binding mode of the 2,3-dihydroxylbenzonate substrate for the Mg-enzyme of 2,3-DHBD_*Ao*. The energy relative to **E:S$_{\mathbf{Mg}}$** is given in kcal/mol.

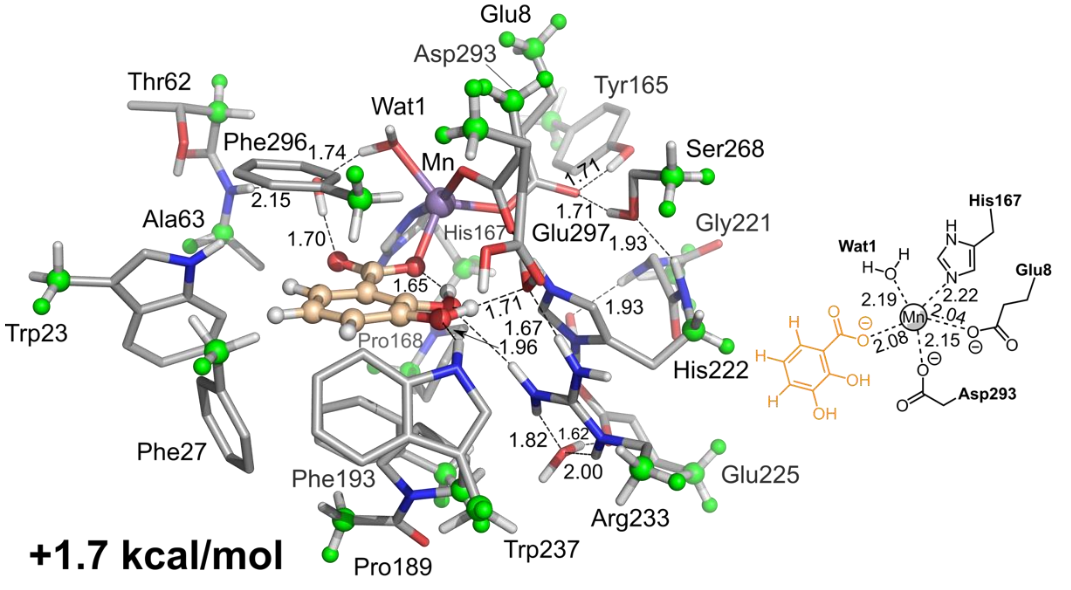

Figure S17. Optimized structure of enzyme-substrate complex with a monodentate binding mode of the 2,3-dihydroxylbenzonate substrate for the Mn-enzyme of 2,3-DHBD_*Ao*. The energy relative to **E:S$_{\mathbf{Mn}}$** is given in kcal/mol.

Cartesian coordinates

The transition states and intermediates involved in the reaction pathway of the Mg-enzyme of 2,3-DHBD_*Ao*

E:S$_{Mg}$ (0.0)

C	4.23175100	3.80121800	5.64166200
C	4.42113800	2.62897300	4.67251200
C	3.56912200	2.84533700	3.40266000
C	3.55013200	1.64000100	2.47650900
O	4.64435200	1.16474700	2.07565400
O	2.42525800	1.12721700	2.13688100
H	4.32939400	4.75788200	5.11683500
H	5.46899700	2.51577800	4.38561400
H	4.12236400	1.69289000	5.16283600
H	2.54275000	3.12165700	3.65631600
H	4.01578700	3.68362000	2.85259400
C	-10.18096900	3.73312400	-0.25954900
C	-8.74529200	3.48352300	-0.58635500
C	-7.71919100	3.51396200	0.33168000
C	-8.11910300	3.38027100	-1.88242000
C	-6.71040800	3.34674900	-1.66197400
C	-8.59805900	3.32939300	-3.20317200
N	-6.49703800	3.44743500	-0.30532500
C	-5.79026900	3.23373200	-2.71086600
C	-7.68694100	3.23498600	-4.24899100
C	-6.29732100	3.18204100	-4.00450900
H	-10.50294200	4.69363200	-0.68596900
H	-10.30130800	3.82452100	0.82582000
H	-7.77200800	3.60256600	1.40815200
H	-5.58409500	3.33883500	0.13346900
H	-9.66538200	3.35837000	-3.40235100
H	-4.72450300	3.18298200	-2.51735300
H	-8.04682400	3.19253900	-5.27235100
H	-5.61113100	3.09504800	-4.84176500
C	-7.95194300	-1.96177000	0.52903500
C	-7.63647600	-1.70466300	-0.92232100
C	-7.25493000	-2.75863400	-1.76420700
C	-7.66828900	-0.40742400	-1.44854000
C	-6.90212700	-2.51935900	-3.09055000
C	-7.29949900	-0.16113600	-2.77261400
C	-6.90906700	-1.21713700	-3.59548300
H	-8.29702400	-1.04907900	1.02366200
H	-7.06249200	-2.30753000	1.06883300
H	-7.22579100	-3.77244300	-1.37470600
H	-7.97625800	0.42315000	-0.81784600
H	-6.62423900	-3.35144700	-3.73006700
H	-7.32372100	0.85060100	-3.15717900
H	-6.61542100	-1.02826500	-4.62350400
C	7.94801300	7.52982700	0.89258900
C	7.73851400	6.08619500	1.28635300
C	8.47997600	5.51823200	2.33037400
C	6.77778500	5.27732300	0.66406000
C	8.25915100	4.21383700	2.75956500
C	6.54151700	3.96495800	1.07836800
C	7.27413300	3.42942700	2.14970900
O	7.07127800	2.18012900	2.63927100
H	8.52987800	8.05539100	1.65568800
H	6.98992900	8.05433700	0.79788300
H	9.23665200	6.11826200	2.82992100
H	6.20376400	5.67873200	-0.16879700
H	8.82867600	3.78517800	3.57738000
H	5.80958100	3.34528200	0.56787500
H	6.20131300	1.83157600	2.33936000
C	3.93551800	4.95489700	-2.55529800
C	2.96400400	4.76379800	-3.70520300
O	2.61911100	5.70654600	-4.42111700
C	3.19872200	5.61762500	-1.36007900
C	2.15827200	4.72164600	-0.77738400
C	2.20839300	3.84647300	0.27787500
N	0.92888600	4.48332900	-1.37267300
C	0.30128500	3.49064100	-0.69395000
N	1.05316200	3.08597400	0.32359200
H	4.38779100	4.01265600	-2.23502000
H	3.92109900	5.85797200	-0.57528400
H	2.75747400	6.55952000	-1.70344100
H	0.61005300	4.90768100	-2.23267800
H	3.00715100	3.72202000	0.98772900
H	-0.67545600	3.10282200	-0.93547800
N	2.40344300	3.53101900	-3.83101900
C	1.52667300	3.24664100	-4.96914500
C	1.32774500	1.72806500	-4.92467400
C	1.59746600	1.35198200	-3.46099000
C	2.71743100	2.31382700	-3.05872600
H	2.02590400	3.56680300	-5.89104000
H	0.33641600	1.43386200	-5.26196800
H	2.06567300	1.23793900	-5.56786700
H	0.70353200	1.51881100	-2.85118600
H	1.90126600	0.31019400	-3.33843100
H	2.72482900	2.52507600	-1.98805900
H	3.70188300	1.92097900	-3.32949500
C	-3.77064500	-2.94780100	-5.17305700
C	-2.59618900	-3.83635600	-4.85338700
O	-1.69783100	-4.08674200	-5.65991900
H	-3.45395400	-1.90451600	-5.12175500
H	-4.60179000	-3.07389300	-4.48248600
N	-2.58195400	-4.33272200	-3.58815900
C	-1.44262300	-5.10610500	-3.11617300
C	-1.83880600	-5.47923000	-1.68879700
C	-2.62257400	-4.24892100	-1.23683800
C	-3.44201700	-3.88785300	-2.47442800
H	-0.53388600	-4.48549100	-3.12561300
H	-2.48693100	-6.35909100	-1.69968200
H	-0.98459200	-5.71032500	-1.05072400
H	-3.25217500	-4.42073000	-0.36755800
H	-1.92581400	-3.44195500	-1.00337800
H	-4.40036400	-4.42378600	-2.48255800
H	-3.65093400	-2.81797400	-2.53202400
C	0.33995600	-1.45741900	-5.37188100
C	-0.88782400	-0.76169200	-4.84030900
C	-1.23411500	-0.87140800	-3.48440400
C	-1.72009400	-0.00065900	-5.67137100
C	-2.37228800	-0.24371900	-2.97707700
C	-2.85895100	0.63518500	-5.16724500
C	-3.19348000	0.51215900	-3.81842100
H	0.22675500	-2.53866000	-5.22738000
H	1.24868100	-1.13514100	-4.85481000
H	-0.61136100	-1.46490200	-2.81877500
H	-1.47514500	0.08704700	-6.72643700
H	-2.62768300	-0.35258800	-1.93117600
H	-3.49244700	1.21204000	-5.83553100
H	-4.09305800	0.97582500	-3.42616900
C	9.75257500	1.89482000	-1.76799000
C	9.22461000	1.01570000	-0.64632800
O	9.96077100	0.45160800	0.15601500
H	8.97359800	2.56391100	-2.14866500
N	7.86525700	0.90157000	-0.62166700
C	7.24864600	-0.06020300	0.24569200

C	7.21015400	-1.47325600	-0.34800200
O	7.40968900	-1.71183300	-1.53783500
H	7.30719800	1.23193900	-1.40819600
H	7.76759300	-0.06427200	1.20660200
H	6.21172900	0.24234000	0.42621200
N	6.85194300	-2.43258300	0.54743200
C	6.40647900	-3.74709200	0.10210400
C	5.24644700	-3.67768200	-0.92518400
C	4.28240800	-2.59751100	-0.52224300
C	3.72891000	-2.31748600	0.70389800
N	3.97138600	-1.51737900	-1.33409900
C	3.28614900	-0.61881400	-0.63167500
N	3.12591200	-1.08411500	0.60817700
H	6.55501900	-2.12627900	1.47050300
H	6.06108200	-4.26917900	1.00102800
H	5.63056600	-3.45439900	-1.92005900
H	4.74789400	-4.65157400	-0.97311200
H	4.29371100	-1.28625200	-2.35389400
H	3.77527600	-2.84830200	1.63697400
H	2.93346900	0.33483600	-0.98555500
C	7.75244500	-3.63470000	-5.28988800
C	7.41773000	-2.22761200	-5.78661100
C	6.81689200	-1.29888000	-4.72694800
C	5.45154900	-1.66089900	-4.15981300
O	5.00702800	-2.83250500	-4.20647600
O	4.82595900	-0.69909000	-3.56817100
H	6.83342500	-4.10587100	-4.93277900
H	8.32130700	-1.74521800	-6.18068200
H	6.72077500	-2.30704300	-6.63076600
H	7.46800800	-1.23958600	-3.84402800
H	6.73890600	-0.27800800	-5.11921800
C	5.24930400	-2.70140500	4.75040300
C	4.96060900	-1.32417800	4.17344000
O	5.45561100	-1.28617900	2.84675600
H	4.94221100	-2.75609000	5.80022500
H	3.87985600	-1.11395800	4.17764700
H	5.44167600	-0.55196400	4.79181600
H	5.20586200	-0.41903900	2.46361400
C	1.14984800	-0.18035200	6.25661600
C	1.15275600	-1.25842500	5.17806800
C	0.78376700	-0.73956200	3.81678600
O	0.20591300	0.33754800	3.63645000
O	1.15465000	-1.54395500	2.83877900
H	1.33788000	0.80014000	5.81108500
H	2.12993100	-1.73833000	5.07850900
H	0.45585500	-2.06666900	5.41741900
C	-3.82584100	-1.19843200	4.29815100
C	-4.19148800	0.21628700	3.92216600
C	-5.48618100	0.52383500	3.48602600
C	-3.26271900	1.25695300	4.03265900
C	-5.84963900	1.83652300	3.19074100
C	-3.62621300	2.57435100	3.74848500
C	-4.92397500	2.87342500	3.32950500
H	-2.74014500	-1.31866700	4.37001100
H	-4.19009000	-1.91354800	3.55463700
H	-6.21380900	-0.27539800	3.37088700
H	-2.24597600	1.02503900	4.34006300
H	-6.85827000	2.04526700	2.84722600
H	-2.89693600	3.37250300	3.83012700
H	-5.19848000	3.90046600	3.10292700
C	-2.36211100	-3.96764800	7.74789800
C	-0.91177200	-4.27625600	7.36844100
C	-0.64260000	-4.38658500	5.85212100
C	-1.21855600	-5.57248200	5.11715300
O	-2.56434700	-5.61962700	5.18214300
O	-0.56475000	-6.37957600	4.48264000
H	-3.01462200	-4.81854000	7.53883600
H	-0.26033800	-3.47579400	7.73975600
H	-0.57473500	-5.19385000	7.86476600
H	-1.05393800	-3.50420500	5.34583500
H	0.43234900	-4.41594800	5.66226800
C	-2.13057600	-0.37597800	0.61138400
C	-2.33313200	1.03041200	0.70643800
C	-3.63798600	1.51049900	0.39544500
C	-4.67953500	0.65849600	0.08524800
C	-4.48139200	-0.73084200	0.09007100
C	-3.22626200	-1.23138800	0.35819100
O	-1.40224600	1.89744300	1.04340100
O	-0.67353900	-2.23865700	0.47552300
O	0.25871800	-0.27892500	0.91739700
Mg	0.44595500	1.57217300	1.78818800
O	0.09825300	3.09691300	3.20546000
O	-0.42117200	-3.96953600	2.50174000
O	-1.82881400	4.35296800	1.76460100
O	5.97714400	1.60216400	-2.74431100
C	1.98417400	-8.34405100	-0.84964300
C	2.93605200	-7.13511400	-0.84059000
N	2.31215800	-5.87294800	-1.25703500
C	1.58497100	-5.06629500	-0.45692400
N	1.24441200	-3.85723100	-0.89690300
N	1.22891800	-5.47291100	0.77308500
H	1.43852200	-8.36901600	-1.79787500
H	1.23181700	-8.28250400	-0.05571800
H	3.39162500	-7.00323800	0.14868100
H	3.75650100	-7.30926500	-1.54036800
H	2.58639000	-5.44540200	-2.14582900
H	1.68458900	-3.53558400	-1.76345700
H	0.64250800	-3.23097600	-0.35030500
H	1.28446400	-6.45280600	0.99311000
H	0.60959200	-4.90358600	1.37069600
C	-3.49850100	-9.01133100	-0.63061700
C	-3.09477300	-7.91443400	0.31790100
C	-1.92627900	-7.81835500	1.03634000
C	-3.90149300	-6.78870800	0.71746500
C	-3.14710400	-6.03862900	1.66820300
C	-5.17511300	-6.32690800	0.34408200
N	-1.94307900	-6.68949700	1.83945600
C	-3.62843400	-4.84743900	2.22824200
C	-5.66131100	-5.15406400	0.90917000
C	-4.89362200	-4.42156300	1.83806800
H	-2.64244600	-9.41233000	-1.18052600
H	-4.24172500	-8.66914400	-1.35523600
H	-1.07282400	-8.48255100	1.04617100
H	-1.24966400	-6.44780100	2.53732900
H	-5.77093700	-6.87861400	-0.37726600
H	-3.02795600	-4.27195600	2.92252100
H	-6.64782000	-4.79357800	0.63813200
H	-5.29598800	-3.50229100	2.25234800
O	2.71458400	-3.83931000	-3.31237600
H	-5.29920800	-1.39399700	-0.15264800
H	-5.65283300	1.06959800	-0.14899800
H	-3.04777600	-2.29564500	0.34889600
C	-0.80103000	-0.99656900	0.66118700
O	-3.85080900	2.88116200	0.36511800
H	-3.40145800	3.30549200	1.11874500
H	-1.78700300	5.07149100	1.11646200
H	-1.65353400	3.48648400	1.29094300
H	-0.29733200	2.73300800	4.00761700
H	-0.53254200	3.76372900	2.84076000
C	-3.78430900	7.80361900	2.29380200
C	-3.39506000	7.91919300	0.83060000
O	-2.98898000	8.95720300	0.32639900
C	-5.29558300	7.60990200	2.49854500
C	-6.14589500	8.61779400	1.73348600
O	-5.59024900	6.25643000	2.08820800
H	-3.29088400	6.92547100	2.72973600
H	-5.50470100	7.69950000	3.57368700
H	-6.51849600	6.20259100	1.82645900
H	-7.21151400	8.46306500	1.93761800
H	-5.89110200	9.63872000	2.03071500
H	-5.98116300	8.53079500	0.65551800
N	-3.55813400	6.74130400	0.13682100

C	-3.66256300	6.76088700	-1.31893300
C	-2.87897100	5.60484100	-1.93993000
H	-4.16040900	6.07461200	0.61157200
H	-3.28395500	7.72749300	-1.65453300
H	-3.03480500	5.55801500	-3.02189500
H	-1.80774800	5.73511700	-1.74751000
H	-3.18834200	4.64837500	-1.50567100
H	0.86489200	-1.10010100	1.92819500
H	2.78117400	-0.46626900	1.35530700
H	-0.83282000	-3.41626400	1.80157400
H	0.25000800	-3.36231600	2.85409000
H	5.63654900	0.72049900	-3.04774200
H	6.41521100	1.96743700	-3.52249000
H	3.57487500	-3.40580100	-3.60429700
H	2.16407200	-3.85676900	-4.10595600
H	-3.39911000	8.68219900	2.82126000
H	-4.72307800	6.69668000	-1.58363700
H	-10.93777900	3.01068200	-0.58386000
H	-8.72720900	-2.72371100	0.66287600
H	-3.93346100	-9.82361300	-0.03902700
H	-4.14550700	-3.12643500	-6.18625100
H	-1.22143100	-5.99224800	-3.72050500
H	2.49642600	-9.30652100	-0.74427000
H	8.13088700	-4.25978300	-6.10556600
H	8.44609100	-3.64408300	-4.44197400
H	7.28398400	-4.29710900	-0.25444800
H	4.71866600	-3.48416100	4.19831200
H	6.33264100	-2.85341000	4.70128200
H	10.58377700	2.51276300	-1.41191000
H	10.06590400	1.25363200	-2.59870700
H	8.47298800	7.67140100	-0.05790600
H	4.94919100	3.81558900	6.46913800
H	3.22535200	3.75250900	6.07011800
H	1.96675100	-0.36893200	6.96147400
H	0.18050200	-0.12821300	6.76435700
H	4.75956600	5.61749100	-2.83799200
H	0.59408000	3.82053600	-4.96013600
H	0.48382900	-1.31209600	-6.44770900
H	-4.26073000	-1.47114400	5.26540600
H	-2.74874200	-3.12621500	7.16323800
H	-2.46742500	-3.71435400	8.80913500
H	-2.86366200	-6.35081400	4.61389100

TS1$_{Mg}$ (+15.8)

C	4.09340400	4.02044500	5.52409600
C	4.32614300	2.80903900	4.61564700
C	3.63216600	3.01413500	3.25281100
C	3.57797500	1.73511000	2.43185800
O	4.66631800	1.20068300	2.07780500
O	2.44489200	1.22951400	2.15315100
H	4.21374000	4.95444700	4.96397500
H	5.39240600	2.64014000	4.44670500
H	3.93218300	1.90516600	5.09830100
H	2.61782200	3.39633900	3.39323400
H	4.20890500	3.76458300	2.69770600
C	-10.17620900	3.80690900	-0.71307900
C	-8.73890300	3.41035600	-0.81844600
C	-7.88128300	3.24319000	0.24436800
C	-7.92943400	3.29508200	-2.00792900
C	-6.58933800	3.06066700	-1.58004000
C	-8.19182100	3.39250500	-3.38571300
N	-6.59466000	3.02328700	-0.20286500
C	-5.52829800	2.92379700	-2.48305700
C	-7.13940700	3.26693100	-4.28567200
C	-5.82064900	3.03640200	-3.83774000
H	-10.36783500	4.70588600	-1.31316400
H	-10.41616700	4.06632300	0.32390800
H	-8.09771000	3.28366300	1.30200300
H	-5.75967900	2.88852400	0.35626000
H	-9.20322400	3.56666900	-3.74150900
H	-4.52176400	2.73395100	-2.13083300
H	-7.33206100	3.34415900	-5.35133000
H	-5.01851900	2.93895400	-4.56339400
C	-8.02030700	-1.86524700	0.38447100
C	-7.58982200	-1.71970000	-1.05080600
C	-7.22904700	-2.84226200	-1.80788500
C	-7.51937500	-0.45572700	-1.64987300
C	-6.81289700	-2.70289500	-3.13040400
C	-7.08757900	-0.31061400	-2.97017300
C	-6.73105800	-1.43583500	-3.71301900
H	-8.39491100	-0.91534600	0.77767600
H	-7.17596300	-2.16410400	1.01887300
H	-7.27595400	-3.83038600	-1.35786300
H	-7.81441500	0.42486100	-1.08485900
H	-6.55822600	-3.58622600	-3.70780900
H	-7.04186500	0.67672700	-3.41401300
H	-6.39448600	-1.32843200	-4.73947200
C	7.95598300	7.49932500	0.70265100
C	7.73787900	6.07704800	1.16523800
C	8.43196000	5.57578000	2.27398300
C	6.81454900	5.22368800	0.54510100
C	8.20144900	4.29500800	2.76505400
C	6.57032700	3.93350900	1.02024000
C	7.25431600	3.46450000	2.15460300
O	7.03992300	2.24367100	2.70121700
H	8.51783700	8.06449600	1.45242600
H	7.00024000	8.01683100	0.55690800
H	9.15878000	6.21091300	2.77474900
H	6.27617500	5.57100800	-0.33497500
H	8.73450200	3.91994500	3.63240200
H	5.86739300	3.27921900	0.51271000
H	6.18322200	1.86570400	2.38472800
C	4.00199600	4.80443600	-2.72101700
C	3.06597400	4.56548100	-3.89100100
O	2.75703900	5.47342500	-4.66597000
C	3.23092400	5.54153800	-1.59238200
C	2.17059600	4.68689400	-0.98343900
C	2.18558500	3.91000600	0.14692600
N	0.97090700	4.37771000	-1.60728600
C	0.33151900	3.43181200	-0.87226100
N	1.04247300	3.13501600	0.20725600
H	4.43467600	3.87802700	-2.33548400
H	3.93034100	5.82683200	-0.80170000
H	2.80647300	6.46206800	-2.00758900
H	0.69122300	4.71011400	-2.51940700
H	2.94937800	3.86260300	0.90391800
H	-0.61909400	2.99430700	-1.13587600
N	2.48634500	3.33695100	-3.96703300
C	1.63468600	3.00981000	-5.11333100
C	1.42848900	1.49398000	-5.01011400
C	1.67557600	1.17126200	-3.52896600
C	2.78276400	2.15188600	-3.13814200
H	2.15834500	3.28810700	-6.03525400
H	0.44123000	1.18970300	-5.35067900
H	2.17358600	0.97765900	-5.62371600
H	0.77071200	1.35039300	-2.93907100
H	1.98530600	0.13682900	-3.36544800
H	2.76268200	2.40343900	-2.07613300
H	3.77523500	1.75272800	-3.36699200
C	-3.71490200	-3.14286600	-5.16546000
C	-2.56478800	-4.03357000	-4.77144300
O	-1.64730400	-4.32773600	-5.54106800
H	-3.37784100	-2.10470100	-5.15548300
H	-4.56440600	-3.22115300	-4.49127400
N	-2.59123600	-4.47920300	-3.48708300
C	-1.45686900	-5.22564300	-2.95826500
C	-1.84787800	-5.49227100	-1.50712000
C	-2.64088000	-4.23519100	-1.14446300
C	-3.45860300	-3.96879100	-2.40827200
H	-0.54988000	-4.60575000	-3.00894700

H	-2.49021500	-6.37365900	-1.44470900
H	-0.98744300	-5.66187000	-0.85815400
H	-3.27532900	-4.35442900	-0.26837200
H	-1.94962100	-3.40787200	-0.96193500
H	-4.41051200	-4.51517800	-2.38198100
H	-3.68059500	-2.90867500	-2.53910100
C	0.41283700	-1.69747600	-5.33235100
C	-0.79168700	-0.99971200	-4.75516400
C	-1.07029900	-1.15451400	-3.38648400
C	-1.66148000	-0.21165000	-5.51754600
C	-2.18349600	-0.55292700	-2.80071000
C	-2.77678800	0.39977400	-4.93351400
C	-3.04859200	0.22588100	-3.57579800
H	0.29502100	-2.77432400	-5.15774000
H	1.33009700	-1.38177800	-4.82471700
H	-0.41171800	-1.76355300	-2.77240300
H	-1.47084600	-0.08722300	-6.58002900
H	-2.38853600	-0.70653600	-1.74645500
H	-3.44691300	0.99436600	-5.54828100
H	-3.93468600	0.66734500	-3.13215300
C	9.76961500	1.73475600	-1.65771000
C	9.21337900	0.90880400	-0.50974600
O	9.93145400	0.37562500	0.32959700
H	9.00407800	2.39169600	-2.08401700
N	7.85398800	0.80503000	-0.50724000
C	7.21109200	-0.11074800	0.39026800
C	7.16956900	-1.54764200	-0.14085700
O	7.42480000	-1.84798700	-1.30633300
H	7.31390600	1.10944500	-1.31628700
H	7.71713400	-0.07967300	1.35769100
H	6.17712100	0.21227700	0.54503200
N	6.75154800	-2.45583200	0.78133500
C	6.32672200	-3.78878100	0.38346100
C	5.21816600	-3.77568100	-0.69538100
C	4.20151700	-2.73396700	-0.34240100
C	3.55377400	-2.50510900	0.84569600
N	3.88712600	-1.66359100	-1.16555600
C	3.08948000	-0.82202700	-0.50875700
N	2.87796600	-1.31737400	0.71090600
H	6.40424600	-2.10259700	1.67116900
H	5.94490900	-4.27045800	1.28998600
H	5.64294400	-3.54991200	-1.67294500
H	4.76013900	-4.76993100	-0.75810100
H	4.26448000	-1.44050300	-2.15858000
H	3.53587100	-3.05289400	1.76863900
H	2.67387600	0.09852500	-0.87858700
C	7.80078400	-3.93141100	-4.97414100
C	7.48757700	-2.54018400	-5.52968800
C	6.87693300	-1.56517600	-4.51569300
C	5.48770900	-1.88725500	-3.97956900
O	5.02091400	-3.04984900	-4.05267000
O	4.87117200	-0.91399200	-3.40254000
H	6.86983700	-4.38029700	-4.61892200
H	8.40281100	-2.08172500	-5.92557400
H	6.80378100	-2.64495700	-6.38168700
H	7.50517600	-1.48503200	-3.61813000
H	6.82762600	-0.55699800	-4.94475500
C	5.07003500	-2.52420700	4.95080100
C	4.81068800	-1.18196800	4.28534800
O	5.19695100	-1.27483000	2.92628100
H	4.73797800	-2.51523300	5.99400100
H	3.74726000	-0.91407400	4.35988800
H	5.37515700	-0.39178600	4.80269600
H	4.98985700	-0.41178700	2.49959800
C	0.95996900	0.09655300	6.24362200
C	0.93367600	-0.82970000	5.02715200
C	-0.05552100	-0.34419000	3.97480400
O	-0.01203200	0.85837800	3.61419000
O	-0.88037000	-1.19689500	3.48548600
H	1.15384000	1.10666700	5.86019800
H	1.90586300	-0.80620900	4.52460000

H	0.73173800	-1.86600100	5.27754900
C	-3.97735700	-0.96703800	4.21344000
C	-4.33142000	0.45185400	3.83770100
C	-5.64673500	0.78044400	3.48526800
C	-3.37957700	1.47702100	3.87018700
C	-6.00412500	2.09449600	3.18725900
C	-3.73303700	2.79397800	3.56623000
C	-5.04776300	3.11334800	3.22388300
H	-2.90241000	-1.11231600	4.29567700
H	-4.36589300	-1.67951300	3.48051500
H	-6.39870400	-0.00387300	3.44470400
H	-2.34858500	1.24371800	4.11394600
H	-7.03516000	2.31971500	2.93369900
H	-2.97769500	3.57232900	3.56986500
H	-5.31285400	4.13763500	2.97485100
C	-2.62106200	-3.58827300	7.81847100
C	-1.15423000	-3.91351800	7.52161300
C	-0.75232200	-3.80180500	6.04852200
C	-1.25689900	-4.79535900	5.03887500
O	-2.34721000	-5.48127700	5.38436400
O	-0.71861200	-4.93180200	3.94406400
H	-3.26694700	-4.44793800	7.62517500
H	-0.51187700	-3.20666800	8.05898700
H	-0.89984300	-4.90737400	7.90653400
H	-1.07063600	-2.83094200	5.64581200
H	0.33631200	-3.82404700	5.94483100
C	-1.86854100	-0.47958100	1.11845400
C	-2.22218000	0.92714400	0.93568500
C	-3.55997400	1.23439400	0.51116200
C	-4.48005000	0.25229200	0.25470600
C	-4.15265700	-1.12051300	0.44404800
C	-2.89769700	-1.46802400	0.84611900
O	-1.41033600	1.90121500	1.12302500
O	-0.26742200	-2.10742200	0.42659400
O	0.45133800	-0.00706800	0.73684200
Mg	0.53048500	1.73621800	1.79829100
O	0.14968800	3.48946400	2.97096200
O	1.08429400	-3.27890200	2.52243700
O	-1.94047200	4.51956800	1.41082100
O	6.03426100	1.42858800	-2.71932100
C	1.88493000	-8.38651600	-0.46574300
C	2.89393200	-7.27786800	-0.82013800
N	2.29096900	-6.02857800	-1.29121100
C	1.69335400	-5.11569200	-0.49657900
N	1.30614900	-3.95959900	-1.02580100
N	1.45404000	-5.39073900	0.79915900
H	1.17210700	-8.51151600	-1.28606100
H	1.30664600	-8.14971200	0.43213100
H	3.55734300	-7.07380300	0.03256600
H	3.54014300	-7.62118800	-1.63064100
H	2.52497300	-5.67760500	-2.22300100
H	1.68490400	-3.72961100	-1.95195100
H	0.83176100	-3.24256600	-0.46736900
H	1.87404200	-6.22086900	1.18305800
H	1.29976300	-4.60318900	1.46086600
C	-3.60820600	-8.99678200	-0.35003800
C	-3.21754200	-7.84823600	0.53701100
C	-2.01039400	-7.65780700	1.16424000
C	-4.05800200	-6.74684300	0.94066600
C	-3.28534700	-5.92110200	1.80971900
C	-5.36740500	-6.35225700	0.61875600
N	-2.04620200	-6.51303000	1.94264700
C	-3.78242800	-4.72285400	2.33912300
C	-5.86864600	-5.16910400	1.15071900
C	-5.08305500	-4.36307200	1.99969700
H	-2.74057600	-9.42946200	-0.85583500
H	-4.33130200	-8.69235100	-1.11182600
H	-1.11306800	-8.25844000	1.11550600
H	-1.27165700	-6.07957000	2.42837800
H	-5.97834100	-6.96032700	-0.04201700
H	-3.16674900	-4.07512900	2.95410200

H	-6.88074800	-4.85750000	0.91323400
H	-5.49751500	-3.43998100	2.39252400
O	2.69338800	-4.07324900	-3.41666400
H	-4.89966000	-1.87741600	0.23439600
H	-5.46818100	0.52938400	-0.09481400
H	-2.61800300	-2.50481200	0.96414200
C	-0.45011100	-0.89088000	0.69987900
O	-3.87735600	2.55635400	0.29816200
H	-3.39953800	3.11252500	0.94017900
H	-1.99607000	5.20395500	0.72433600
H	-1.67322200	3.67387200	0.98926900
H	-0.24294900	3.15152500	3.78723600
H	-0.51179700	4.08199800	2.55458000
C	-3.80345900	7.93462300	1.80790500
C	-3.37271700	7.97197600	0.35330300
O	-2.93075200	8.97436800	-0.18854900
C	-5.31998200	7.76182200	1.98731300
C	-6.14683500	8.72431300	1.14198700
O	-5.60679300	6.38716300	1.65111100
H	-3.32973500	7.07547100	2.29995200
H	-5.55300100	7.91780800	3.04992800
H	-6.54605600	6.30309500	1.44151500
H	-7.21791500	8.58577900	1.32825900
H	-5.89464400	9.76061200	1.38322400
H	-5.95695900	8.57097700	0.07563300
N	-3.53809300	6.76165100	-0.29064300
C	-3.60570400	6.72922100	-1.75087200
C	-2.82086200	5.54418000	-2.31115700
H	-4.18430200	6.14132000	0.19176200
H	-3.20866000	7.67905000	-2.11153600
H	-2.93263900	5.47534100	-3.39730900
H	-1.75621300	5.65340400	-2.07528000
H	-3.17615300	4.60373400	-1.87780900
H	-1.50256800	-0.72070100	2.39908800
H	2.34578500	-0.79366100	1.40086700
H	0.54496700	-2.75627900	1.89003400
H	0.43895200	-3.63543200	3.15819100
H	5.68035600	0.53109500	-2.95432700
H	6.52804600	1.69932400	-3.50279400
H	3.57402200	-3.61731700	-3.61003900
H	2.21431100	-4.07202900	-4.25469000
H	-3.42193800	8.83303800	2.30429700
H	-4.66040300	6.66142200	-2.03710300
H	-10.93167500	3.07687800	-1.02239600
H	-8.80567400	-2.61359500	0.53367500
H	-4.06369800	-9.77827600	0.26740300
H	-4.06741000	-3.36402900	-6.17832000
H	-1.22955800	-6.13980000	-3.51660700
H	2.38519900	-9.34753900	-0.30653800
H	8.19241800	-4.59569000	-5.75178400
H	8.47401500	-3.90796900	-4.11062900
H	7.20725800	-4.36133700	0.07294800
H	4.54516800	-3.32658400	4.42183900
H	6.15269400	-2.68729300	4.93458100
H	10.59766000	2.36129600	-1.30982800
H	10.09615900	1.05412600	-2.45090200
H	8.50457700	7.59332300	-0.24037600
H	4.79114700	4.06620900	6.36681600
H	3.07664100	3.99965400	5.92984800
H	1.75853900	-0.06648200	6.97482300
H	-0.02038400	0.17997500	6.72451100
H	4.83852400	5.44764800	-3.01403600
H	0.70749800	3.59105600	-5.15224600
H	0.58405100	-1.60242600	-6.40986500
H	-4.43739800	-1.19189000	5.18128000
H	-2.98691000	-2.77287700	7.18717900
H	-2.74998800	-3.28841800	8.86460200
H	-2.62493500	-6.01994900	4.61981900

Int$_{Mg}$ (+8.8)

C	4.16105200	3.92690000	5.62341100
C	4.37756000	2.73337000	4.68613700
C	3.58574300	2.93126200	3.37736800
C	3.53424500	1.68195700	2.50535800
O	4.62391600	1.19596200	2.08967800
O	2.40544400	1.16411500	2.24079900
H	4.26234100	4.87347400	5.08120300
H	5.43510800	2.60310500	4.44524600
H	4.04638800	1.81163000	5.18166000
H	2.56444600	3.25756800	3.59461100
H	4.08595900	3.72880500	2.81293900
C	-10.19367600	3.71364000	-0.41493900
C	-8.74717100	3.41274400	-0.66222100
C	-7.76921600	3.38311600	0.30736100
C	-8.05826000	3.28999700	-1.92556100
C	-6.66513400	3.18699300	-1.63561300
C	-8.47000600	3.28318400	-3.26965500
N	-6.51934200	3.24824200	-0.26684200
C	-5.69687100	3.06616900	-2.63998100
C	-7.51099900	3.17615800	-4.27064800
C	-6.13868600	3.06740600	-3.95830500
H	-10.47291200	4.65385300	-0.90969500
H	-10.36102600	3.86581600	0.65700500
H	-7.87426000	3.47420500	1.37896600
H	-5.62984700	3.14448500	0.21009400
H	-9.52353700	3.36141700	-3.52187100
H	-4.64617400	2.97288600	-2.39146800
H	-7.81998200	3.17091200	-5.31133200
H	-5.41370700	2.97822500	-4.76188700
C	-7.94878000	-1.96006500	0.47624400
C	-7.57406600	-1.75335500	-0.96721000
C	-7.17759500	-2.83796300	-1.76160500
C	-7.58383600	-0.47380200	-1.53662500
C	-6.79958400	-2.64669000	-3.08881000
C	-7.18930500	-0.27625300	-2.86187900
C	-6.79185600	-1.36333300	-3.63991000
H	-8.31915200	-1.03242400	0.92258000
H	-7.07929500	-2.27786100	1.06550900
H	-7.16203100	-3.83728600	-1.33479100
H	-7.90402400	0.37817000	-0.94152500
H	-6.51397700	-3.50177200	-3.69310600
H	-7.20192600	0.72151100	-3.28250900
H	-6.48113000	-1.21340000	-4.66920100
C	7.90765800	7.60358100	0.85765900
C	7.68636200	6.16341000	1.26350200
C	8.42126000	5.59887600	2.31441400
C	6.72221100	5.35328200	0.64748500
C	8.19225400	4.29934700	2.75366000
C	6.47713200	4.04527300	1.07236500
C	7.20421900	3.51171500	2.14981800
O	6.99886100	2.27010700	2.64929800
H	8.48643500	8.13211200	1.62116200
H	6.95346700	8.13301100	0.75064000
H	9.18048000	6.19826600	2.81121200
H	6.15197700	5.75039800	-0.19033700
H	8.75838400	3.87491100	3.57607800
H	5.74019500	3.42636000	0.56823800
H	6.13084000	1.90050900	2.35072900
C	3.93959500	4.96233100	-2.59137500
C	2.98510000	4.75183200	-3.75271900
O	2.63895700	5.68046500	-4.48523000
C	3.16119100	5.57691400	-1.39341400
C	2.12896000	4.65253400	-0.82150000
C	2.13405900	3.87209000	0.31105200
N	0.93725600	4.33507800	-1.45527500
C	0.29734700	3.38015500	-0.72904300
N	0.99611300	3.08661900	0.36378200
H	4.40717700	4.02499700	-2.27761500
H	3.86354400	5.82506400	-0.59312100
H	2.69618400	6.51196000	-1.72533600

H	0.67158600	4.65294600	-2.37702800
H	2.88606200	3.83617700	1.07979100
H	-0.63775400	2.92379600	-1.01731000
N	2.42724500	3.51313600	-3.86115600
C	1.56122100	3.21044000	-5.00427100
C	1.38096300	1.68806200	-4.95642000
C	1.69924000	1.30403200	-3.50335700
C	2.79575100	2.29503400	-3.11101200
H	2.06573900	3.52917200	-5.92413300
H	0.38350700	1.38307600	-5.26473000
H	2.10265300	1.20957500	-5.62579800
H	0.81425100	1.43095000	-2.87078900
H	2.04417100	0.27283500	-3.40222200
H	2.82011200	2.48688900	-2.03683400
H	3.78698500	1.93818900	-3.40563500
C	-3.70833800	-3.00893100	-5.17075300
C	-2.53307300	-3.88685800	-4.82483700
O	-1.61783500	-4.12903800	-5.61540800
H	-3.39365700	-1.96432400	-5.13131000
H	-4.54631000	-3.12745900	-4.48851100
N	-2.53761700	-4.37793400	-3.55906300
C	-1.39168400	-5.12809600	-3.06108200
C	-1.79732500	-5.48339800	-1.63009700
C	-2.63907600	-4.27824700	-1.20736800
C	-3.43579000	-3.95364900	-2.46915200
H	-0.49396300	-4.49647200	-3.07738200
H	-2.40935100	-6.38908300	-1.63244200
H	-0.94542400	-5.66406100	-0.97392000
H	-3.29084700	-4.47630900	-0.35948000
H	-1.98420000	-3.44290100	-0.95475700
H	-4.37740000	-4.51859800	-2.49931400
H	-3.67582300	-2.89140700	-2.53957700
C	0.39735800	-1.50400600	-5.35118500
C	-0.81216200	-0.83383000	-4.75660800
C	-1.08971700	-1.02720100	-3.39406500
C	-1.68858700	-0.03461900	-5.50013000
C	-2.20860600	-0.45104200	-2.79685900
C	-2.80863800	0.55416300	-4.90223400
C	-3.07904000	0.34314300	-3.54942600
H	0.29512100	-2.58603500	-5.20409100
H	1.31452800	-1.18871500	-4.84377000
H	-0.43190400	-1.64683300	-2.79099600
H	-1.49794900	0.11768000	-6.55900900
H	-2.41379000	-0.64821300	-1.75243100
H	-3.48186100	1.16019100	-5.50238100
H	-3.96778800	0.76719600	-3.09349200
C	9.76135400	1.93905900	-1.70497000
C	9.22205900	1.07367400	-0.57661400
O	9.95171400	0.52492400	0.24238400
H	8.98464400	2.60078700	-2.10332800
N	7.86379100	0.95439900	-0.56905800
C	7.23282700	0.00587000	0.30342600
C	7.21611000	-1.41913400	-0.26282200
O	7.49145100	-1.69012200	-1.43086100
H	7.31544500	1.28268000	-1.36289500
H	7.73628700	0.01976700	1.27250300
H	6.19357500	0.30908300	0.46567900
N	6.80474600	-2.35381300	0.63543900
C	6.42043400	-3.69003600	0.21295500
C	5.29759100	-3.68793000	-0.84462900
C	4.21561900	-2.73599600	-0.43773000
C	3.48996700	-2.64631100	0.72504500
N	3.84502700	-1.64646300	-1.21049700
C	2.92980900	-0.93276000	-0.55645900
N	2.70345600	-1.52524600	0.61742800
H	6.43550500	-2.02939200	1.52857500
H	6.06535800	-4.20203400	1.11405000
H	5.69579100	-3.39147000	-1.81444100
H	4.89763200	-4.70295100	-0.95299800
H	4.25758400	-1.34169900	-2.16933000
H	3.46641200	-3.25479400	1.61053900

H	2.43974400	-0.03714600	-0.89541500
C	7.81843000	-3.64846100	-5.16670000
C	7.46430900	-2.24509000	-5.66527100
C	6.86181100	-1.31686000	-4.60222600
C	5.49594200	-1.68630400	-4.03411900
O	5.03657300	-2.84596800	-4.16723100
O	4.89367500	-0.75689800	-3.37501000
H	6.90242500	-4.12688700	-4.80998100
H	8.35960900	-1.75412300	-6.06783100
H	6.76040400	-2.33438400	-6.50247800
H	7.51559000	-1.24726200	-3.72251300
H	6.77575100	-0.29829900	-5.00078700
C	5.21391800	-2.58323000	4.83464100
C	4.91764800	-1.23520500	4.19239000
O	5.16913000	-1.33387300	2.79973800
H	4.89732400	-2.59988300	5.88214500
H	3.87148300	-0.95142700	4.36522200
H	5.54045900	-0.45291300	4.65113100
H	4.93365800	-0.46501500	2.40065500
C	1.08965900	-0.05906200	6.26553000
C	1.10863600	-0.98930600	5.05695300
C	0.02672500	-0.66423100	4.02968900
O	-0.12804300	0.55728100	3.69267000
O	-0.64525800	-1.61756900	3.54698700
H	1.25838400	0.95946700	5.89348800
H	2.05859400	-0.86476200	4.52721100
H	1.03736500	-2.03946800	5.33401600
C	-3.86251300	-1.12553400	4.27362900
C	-4.32973800	0.22862500	3.80416800
C	-5.62964700	0.40379100	3.31362300
C	-3.48246000	1.34305500	3.85196700
C	-6.08122800	1.65801400	2.90714400
C	-3.93360100	2.60226700	3.45125500
C	-5.23729400	2.76929300	2.98363300
H	-2.77656800	-1.17130900	4.36655500
H	-4.16086400	-1.90365100	3.56356000
H	-6.29479300	-0.45360400	3.24695800
H	-2.45693100	1.20753800	4.18285800
H	-7.09271100	1.75924100	2.52773300
H	-3.26440900	3.45625700	3.48571300
H	-5.57610000	3.75699300	2.68205000
C	-2.42071800	-3.83842500	7.77685500
C	-0.94566200	-4.11200900	7.46543400
C	-0.54878100	-4.08467800	5.98215000
C	-1.02004100	-5.17799200	5.06565500
O	-2.24302400	-5.65091300	5.31807500
O	-0.35795900	-5.56992500	4.10735400
H	-3.04722600	-4.70724400	7.56480500
H	-0.33221400	-3.34221800	7.94741400
H	-0.63796400	-5.06504300	7.91166200
H	-0.89648200	-3.15672500	5.50429500
H	0.53981200	-4.07929300	5.88716400
C	-2.02312900	-0.50656300	1.08927700
C	-2.27840700	0.94796700	0.87429700
C	-3.53257800	1.36622100	0.28727700
C	-4.47939600	0.44596700	-0.05377100
C	-4.26835100	-0.96392200	0.15874200
C	-3.11762300	-1.42273000	0.69497600
O	-1.44198000	1.83283200	1.17987000
O	-0.51333000	-2.04901200	0.00479100
O	0.35101500	-0.14384400	0.81091000
Mg	0.47569500	1.54374900	1.94439300
O	-0.09345200	3.19801000	3.19076900
O	1.08924900	-3.57467800	2.74703600
O	-2.00796600	4.57037200	1.24704700
O	6.04386200	1.61208200	-2.77027200
C	2.02639400	-8.31875500	-0.71520100
C	2.98932200	-7.13009500	-0.89754700
N	2.35363100	-5.90213700	-1.38691100
C	1.65553500	-5.02472900	-0.63406600
N	1.27173400	-3.87465800	-1.17513200

N	1.31137900	-5.32303200	0.63162600
H	1.38723100	-8.40408800	-1.59924800
H	1.36377600	-8.17747400	0.14445400
H	3.52996600	-6.92083700	0.03577500
H	3.75047600	-7.38568700	-1.63797100
H	2.61432000	-5.53917600	-2.30689500
H	1.69545400	-3.62266800	-2.07467800
H	0.70825400	-3.19291800	-0.65261100
H	1.69710900	-6.15912600	1.03840200
H	1.09169700	-4.55920100	1.29728100
C	-3.45561900	-9.00594600	-0.54014500
C	-3.04654100	-7.89889200	0.39184500
C	-1.84123200	-7.75980800	1.03674900
C	-3.86897600	-6.80234300	0.83945900
C	-3.08530100	-6.02594300	1.74475800
C	-5.17254400	-6.37643900	0.53457400
N	-1.85752800	-6.64266500	1.85432600
C	-3.56378300	-4.84129600	2.32010300
C	-5.65677900	-5.20974300	1.11553500
C	-4.85890800	-4.44916000	1.99484000
H	-2.60007600	-9.41365200	-1.08555600
H	-4.19874900	-8.67116100	-1.26882200
H	-0.95865700	-8.38108200	0.97734100
H	-1.09990600	-6.30723900	2.43612500
H	-5.79136300	-6.94734700	-0.15153700
H	-2.94136500	-4.22875600	2.96504000
H	-6.66469500	-4.87465300	0.89255400
H	-5.25997200	-3.53495300	2.42097200
O	2.75468300	-3.94942300	-3.51288400
H	-5.05555200	-1.64623000	-0.13874400
H	-5.41339100	0.77626300	-0.49335700
H	-2.95334000	-2.47872100	0.85574500
C	-0.60182500	-0.94446800	0.56117300
O	-3.71551000	2.70357100	0.08868400
H	-3.05557400	3.22222300	0.61320600
H	-2.32236200	5.41096500	0.85339700
H	-1.22166400	4.31376400	0.74087700
H	-0.44346600	2.68184100	3.93370300
H	-0.83971100	3.68349700	2.79661200
C	-3.83912300	7.84716000	2.14187300
C	-3.39548800	7.91789500	0.69388500
O	-2.89418800	8.90802500	0.18814300
C	-5.36042000	7.69032600	2.30529700
C	-6.16191300	8.71507300	1.51028000
O	-5.67655700	6.34225600	1.89355000
H	-3.37324300	6.97622200	2.62027700
H	-5.59457100	7.79208600	3.37377400
H	-6.61710000	6.29068100	1.67909000
H	-7.23685700	8.58505000	1.67867400
H	-5.89525000	9.73079600	1.81504400
H	-5.96502500	8.62208500	0.43804200
N	-3.61358300	6.73009100	0.01023700
C	-3.67751900	6.75416900	-1.45480200
C	-2.87363600	5.61029600	-2.07005200
H	-4.33577100	6.16408300	0.45579300
H	-3.29912100	7.72448500	-1.77964600
H	-2.99406700	5.59121800	-3.15713700
H	-1.80862600	5.73455400	-1.84308800
H	-3.19901300	4.64360400	-1.67456500
H	-1.82708500	-0.64974400	2.17262200
H	2.01902000	-1.14629900	1.26566700
H	0.40693800	-2.88073200	2.89831300
H	0.83110700	-4.27356900	3.37240300
H	5.68486700	0.70695800	-2.96688600
H	6.57297800	1.82802000	-3.54774600
H	3.61928900	-3.46491400	-3.70707900
H	2.28237500	-3.97610600	-4.35434700
H	-3.46203000	8.73489400	2.66037200
H	-4.73533500	6.68114400	-1.72805000
H	-10.94409200	2.98348200	-0.73600500
H	-8.72215700	-2.72310000	0.61339400
H	-3.89205900	-9.81193200	0.05894000
H	-4.07266900	-3.20360000	-6.18485100
H	-1.16056800	-6.02139500	-3.65048600
H	2.54179000	-9.27742600	-0.59215700
H	8.20685900	-4.28337300	-5.96990900
H	8.50331700	-3.64261900	-4.31231800
H	7.30356900	-4.24113200	-0.12721000
H	4.69190700	-3.37607100	4.28862800
H	6.29829800	-2.73133900	4.79801200
H	10.58617700	2.56563700	-1.34965700
H	10.08508100	1.28751900	-2.52335300
H	8.44123200	7.73387800	-0.08966200
H	4.87025200	3.95617400	6.45724700
H	3.15060700	3.88007200	6.04275800
H	1.90052400	-0.23348400	6.98030000
H	0.11517900	-0.00320500	6.76269800
H	4.76335300	5.62537700	-2.87617200
H	0.62608400	3.78007700	-5.01209500
H	0.55172200	-1.37345000	-6.42754700
H	-4.30569300	-1.38641600	5.24036900
H	-2.80534300	-3.00819100	7.17584600
H	-2.53758600	-3.57191800	8.83274400
H	-2.48725600	-6.26850800	4.60468200

$TS2_{Mg}$ (+11.7)

C	4.14411100	3.95742700	5.57086200
C	4.37126600	2.77863600	4.61317000
C	3.59510500	3.00026900	3.29880500
C	3.55802500	1.79113300	2.36811400
O	4.65107400	1.25390900	2.03423900
O	2.43437700	1.37224600	1.94698800
H	4.22831800	4.90983500	5.03559400
H	5.43236000	2.65452700	4.38513000
H	4.03653700	1.84683600	5.08735600
H	2.57124400	3.32007000	3.51015200
H	4.09391600	3.81631900	2.75812500
C	-10.26331600	3.57699300	-0.33202600
C	-8.82325800	3.29507900	-0.62150300
C	-7.81927300	3.28578800	0.32046200
C	-8.16956400	3.17388300	-1.90302900
C	-6.76755400	3.09598600	-1.65106300
C	-8.61838900	3.14810400	-3.23489300
N	-6.58561700	3.16692500	-0.28792300
C	-5.82502000	2.98879300	-2.68109100
C	-7.68580300	3.04893200	-4.26122300
C	-6.30331700	2.97042700	-3.98662800
H	-10.56865500	4.51807100	-0.80942100
H	-10.40495200	3.71499700	0.74547200
H	-7.89575700	3.38588800	1.39374800
H	-5.68276300	3.06869000	0.16368300
H	-9.67971300	3.20605000	-3.45823700
H	-4.76496500	2.92323100	-2.46457000
H	-8.02325300	3.02879800	-5.29283700
H	-5.59922100	2.89092100	-4.80959300
C	-7.95395600	-2.07044900	0.56104400
C	-7.58730000	-1.85649100	-0.88349900
C	-7.20091600	-2.93633400	-1.68906200
C	-7.59563600	-0.57246800	-1.44317100
C	-6.83173200	-2.73621200	-3.01775000
C	-7.21250700	-0.36681700	-2.77040700
C	-6.82430600	-1.44914600	-3.55955300
H	-8.32998700	-1.14628400	1.01025900
H	-7.07526500	-2.37711700	1.14304500
H	-7.19019400	-3.93976400	-1.27154600
H	-7.90743800	0.27663900	-0.83969900
H	-6.55563500	-3.58705400	-3.63210200
H	-7.22745900	0.63329400	-3.18472500
H	-6.52230800	-1.29231600	-4.59040600
C	7.80950900	7.65122800	0.75540400

C	7.62454700	6.21124600	1.17809800
C	8.37171900	5.67906300	2.23692500
C	6.68231900	5.36988900	0.57021100
C	8.17428000	4.37991400	2.69259800
C	6.47040300	4.06161300	1.01036900
C	7.20778400	3.56095200	2.09651200
O	7.02939500	2.32085900	2.61148400
H	8.38140800	8.20130600	1.50863400
H	6.84253800	8.15716700	0.64972500
H	9.11446500	6.30389700	2.72726200
H	6.10238000	5.74190400	-0.27251200
H	8.74836000	3.98065400	3.52213400
H	5.75118500	3.41841200	0.51155800
H	6.16475600	1.93951800	2.32122900
C	3.83594500	4.95652000	-2.64566000
C	2.89402600	4.72132200	-3.81076800
O	2.58596200	5.62964100	-4.58540300
C	3.06403100	5.66603500	-1.49771900
C	2.00295800	4.81828300	-0.87618600
C	2.00258800	4.08744600	0.28469700
N	0.79226000	4.51307800	-1.48240000
C	0.13195600	3.61528600	-0.70550200
N	0.84061600	3.34332800	0.38193700
H	4.29227400	4.03273200	-2.28135900
H	3.76938600	5.94501600	-0.70996200
H	2.63552100	6.59197700	-1.89727700
H	0.51211900	4.82058100	-2.40303000
H	2.76758100	4.04891400	1.04085300
H	-0.83175600	3.19429900	-0.94847400
N	2.31249100	3.49346100	-3.88589400
C	1.45274600	3.17116900	-5.02906700
C	1.26985100	1.65203700	-4.93936400
C	1.50322000	1.32749100	-3.45611100
C	2.60964200	2.30642800	-3.05848100
H	1.95513400	3.47629200	-5.95341500
H	0.29237900	1.33434900	-5.29542500
H	2.03219100	1.15216100	-5.54580300
H	0.59472900	1.51168100	-2.87308600
H	1.80535400	0.29156900	-3.28579000
H	2.58619200	2.55357100	-1.99533500
H	3.60366100	1.90966500	-3.28740000
C	-3.75604900	-3.10070500	-5.12105200
C	-2.57082800	-3.96745400	-4.78040600
O	-1.66371800	-4.21675300	-5.57705900
H	-3.46000800	-2.05114400	-5.08088900
H	-4.58637200	-3.24048300	-4.43241200
N	-2.55878200	-4.45015400	-3.50866800
C	-1.39895900	-5.18802500	-3.02457100
C	-1.76195600	-5.50692400	-1.57417300
C	-2.58221000	-4.28302900	-1.15553900
C	-3.41926000	-3.99082300	-2.40157600
H	-0.50597900	-4.54870700	-3.07244100
H	-2.38305100	-6.40493500	-1.53157600
H	-0.88930200	-5.68034400	-0.94271400
H	-3.20526900	-4.45205000	-0.27953600
H	-1.91893900	-3.43949100	-0.95136300
H	-4.36261800	-4.55265600	-2.38185100
H	-3.65863600	-2.93046000	-2.49553700
C	0.33258700	-1.55599100	-5.34594700
C	-0.89252300	-0.88951800	-4.77886300
C	-1.18232000	-1.04652700	-3.41542000
C	-1.76714000	-0.11973100	-5.55468400
C	-2.30828400	-0.45736900	-2.84367600
C	-2.89627400	0.47880200	-4.98483200
C	-3.17591000	0.30828400	-3.62824100
H	0.24511500	-2.63757800	-5.18652700
H	1.24214500	-1.21420100	-4.84191800
H	-0.52128800	-1.64118000	-2.79217100
H	-1.56597500	0.00503300	-6.61521500
H	-2.52014300	-0.61061600	-1.79267600
H	-3.56682000	1.06387400	-5.60826300
H	-4.06826300	0.74482700	-3.19176600
C	9.69547400	1.99472100	-1.80145000
C	9.18656100	1.12227000	-0.66544500
O	9.93760400	0.57351100	0.13417600
H	8.90498500	2.64470500	-2.19145400
N	7.83046800	0.99351000	-0.62862900
C	7.22584500	0.03868200	0.25448200
C	7.21464900	-1.38651800	-0.31070200
O	7.47303500	-1.65680500	-1.48251100
H	7.25967100	1.32966600	-1.40283400
H	7.74338000	0.05831600	1.21614500
H	6.18605200	0.33113200	0.43098100
N	6.82137700	-2.32418000	0.59246700
C	6.42861200	-3.65915800	0.17041100
C	5.27342900	-3.64735200	-0.85382100
C	4.22144500	-2.68144500	-0.39621700
C	3.52656000	-2.61485000	0.78493500
N	3.89503100	-1.53178200	-1.10023900
C	3.04388600	-0.79788100	-0.38230700
N	2.79955100	-1.44466800	0.76168700
H	6.47481300	-2.00229300	1.49476300
H	6.09167100	-4.17337600	1.07739400
H	5.63906300	-3.35344000	-1.83685400
H	4.85828900	-4.65752300	-0.94800800
H	4.27290300	-1.24557800	-2.07913800
H	3.49319700	-3.27032500	1.63769600
H	2.61870500	0.15218700	-0.65284400
C	7.77601500	-3.62600000	-5.22219000
C	7.38946100	-2.22585200	-5.70771000
C	6.80722400	-1.30383600	-4.62555100
C	5.45620200	-1.67378400	-4.02156800
O	4.99536100	-2.83519600	-4.14665200
O	4.86979200	-0.74565300	-3.34821800
H	6.87224900	-4.11832000	-4.85140800
H	8.26697600	-1.72240800	-6.13335100
H	6.66408500	-2.32178600	-6.52563400
H	7.48212400	-1.23439400	-3.76221900
H	6.71068500	-0.28365700	-5.01720700
C	5.25430900	-2.54524400	4.79861700
C	4.92872100	-1.20077200	4.16221700
O	5.30614800	-1.24301800	2.79655300
H	4.94677900	-2.56591500	5.84916300
H	3.85338900	-0.98628500	4.24105800
H	5.45676000	-0.39354900	4.69129400
H	5.04700600	-0.38301000	2.39726900
C	1.11861500	-0.05594900	6.25778400
C	1.12768500	-1.05348700	5.10361600
C	0.68148400	-0.52870800	3.73956500
O	0.23453700	0.65147900	3.62529500
O	0.78675000	-1.33005200	2.76177900
H	1.28584400	0.94691600	5.84877600
H	2.12652700	-1.46914900	4.94541400
H	0.49975400	-1.91752900	5.31969800
C	-3.84098500	-1.17973500	4.31666200
C	-4.32456500	0.17240900	3.85707100
C	-5.59572500	0.31536600	3.28886400
C	-3.52846100	1.31392000	4.00537000
C	-6.06820300	1.56646400	2.89720100
C	-3.99912900	2.56990100	3.61892400
C	-5.27393700	2.70378100	3.06596000
H	-2.75124600	-1.19165000	4.42165100
H	-4.11959700	-1.96204000	3.60554400
H	-6.21842800	-0.56348900	3.14295600
H	-2.52142000	1.20833700	4.40106300
H	-7.05395400	1.64345800	2.45079400
H	-3.36426900	3.44353900	3.71594800
H	-5.62591000	3.68843000	2.76877300
C	-2.33931400	-3.86310800	7.81711400
C	-0.87094600	-4.13650600	7.48543200
C	-0.53278300	-4.12693600	5.98920200
C	-0.96392900	-5.26785400	5.11051400

O	-2.12420200	-5.84031200	5.43324900
O	-0.32358800	-5.60425100	4.11909200
H	-2.96490300	-4.73610600	7.61783800
H	-0.24821400	-3.35610400	7.93690800
H	-0.54735800	-5.08177100	7.93548100
H	-0.99292800	-3.24045100	5.52906600
H	0.54422300	-4.03270900	5.83382600
C	-2.06193300	-0.37099000	1.26210000
C	-2.33288700	1.02539900	1.04914900
C	-3.54055900	1.37792300	0.36231100
C	-4.42813100	0.41175500	-0.04315500
C	-4.17404900	-0.96724600	0.20078500
C	-3.04060300	-1.34473500	0.86921000
O	-1.50601200	1.94478400	1.41239600
O	-0.37777700	-1.77473500	-0.26353300
O	0.30454200	0.34303800	0.27915800
Mg	0.48538100	1.78508400	1.89132200
O	0.09343900	3.36110100	3.28917400
O	1.57591000	-3.86633900	2.91289000
O	-2.10391700	4.47072300	1.77124700
O	5.90908000	1.66430200	-2.73889400
C	2.07256100	-8.33498500	-0.69791900
C	3.04533100	-7.18580500	-1.01711400
N	2.39407200	-5.94674100	-1.44891900
C	1.76542700	-5.08337500	-0.62714900
N	1.33686100	-3.93018700	-1.13153300
N	1.53332000	-5.40250300	0.65816400
H	1.35914100	-8.45077900	-1.51911300
H	1.49155100	-8.14886000	0.21003700
H	3.70136200	-6.98239300	-0.15903700
H	3.70125400	-7.48071800	-1.83865100
H	2.60365900	-5.56193700	-2.37396100
H	1.71762800	-3.65279800	-2.04618300
H	0.83678000	-3.25106500	-0.56855600
H	1.96564700	-6.24470600	1.00203000
H	1.43090800	-4.66483100	1.38361900
C	-3.40085400	-9.07561500	-0.46883100
C	-2.98901200	-7.96161400	0.44988300
C	-1.78132900	-7.82152800	1.08843600
C	-3.80973900	-6.86314800	0.89590600
C	-3.02547300	-6.08954400	1.80221600
C	-5.11231500	-6.43513500	0.58967400
N	-1.79698700	-6.70603100	1.90793200
C	-3.50962400	-4.91275500	2.38786300
C	-5.59720700	-5.27009800	1.17295000
C	-4.80298400	-4.51655000	2.06136300
H	-2.54923400	-9.47996200	-1.02302000
H	-4.15525700	-8.75176900	-1.19076800
H	-0.89783700	-8.44112400	1.02521200
H	-1.03083800	-6.36406700	2.47319900
H	-5.73033700	-7.00385400	-0.09887600
H	-2.89378400	-4.31347000	3.05111700
H	-6.60320700	-4.93190100	0.94715600
H	-5.20781000	-3.60755300	2.49490000
O	2.71687400	-3.91903200	-3.50330600
H	-4.89609000	-1.69953500	-0.14234700
H	-5.33767700	0.70492500	-0.55461500
H	-2.84351100	-2.39045000	1.07258400
C	-0.37694900	-0.66025300	0.18806600
O	-3.80628100	2.71068900	0.13003800
H	-3.39138700	3.25823900	0.82476800
H	-1.95595600	5.13648600	1.08276800
H	-1.76212200	3.60175200	1.44164300
H	-0.24110000	2.87842700	4.05841700
H	-0.61647400	3.96156700	2.98597300
C	-3.92666300	7.78378100	2.14841800
C	-3.53751500	7.87694200	0.68340800
O	-3.14754000	8.91090300	0.15900100
C	-5.43816800	7.58896400	2.35321800
C	-6.28716800	8.59525500	1.58425500
O	-5.73631500	6.23497000	1.94820900

H	-3.42900000	6.91711100	2.60135500
H	-5.64664900	7.68378400	3.42817200
H	-6.66683400	6.18451500	1.69472200
H	-7.35324500	8.44151700	1.78733900
H	-6.03276100	9.61704700	1.87882000
H	-6.12057100	8.50535500	0.50679300
N	-3.68233600	6.68292400	0.01090300
C	-3.78766000	6.67750400	-1.44516700
C	-2.98352800	5.53217200	-2.05752800
H	-4.27989200	6.02111700	0.49826600
H	-3.42694600	7.64649100	-1.79377900
H	-3.12927300	5.48580300	-3.14103300
H	-1.91648800	5.67635500	-1.85521300
H	-3.28510900	4.56963700	-1.63221600
H	-1.42905300	-0.60160100	2.11604300
H	2.17202100	-1.11371000	1.49983000
H	1.23830400	-2.93212100	2.91687400
H	0.95923600	-4.39510900	3.44706500
H	5.60240900	0.74629900	-2.96307700
H	6.35623400	1.96740100	-3.53833500
H	3.58821900	-3.43546600	-3.69110800
H	2.22825900	-3.89925600	-4.33573900
H	-3.55358500	8.67739500	2.65972000
H	-4.84723400	6.59299100	-1.70823300
H	-11.00941800	2.83814700	-0.64324600
H	-8.71842300	-2.84052100	0.70852800
H	-3.82339100	-9.88353400	0.13769400
H	-4.12787100	-3.30315800	-6.13089400
H	-1.16440800	-6.08147700	-3.61245500
H	2.59830000	-9.28799500	-0.57561000
H	8.16325600	-4.26043800	-6.02639300
H	8.46875700	-3.60991900	-4.37425900
H	7.31407400	-4.20290000	-0.17571200
H	4.73509700	-3.34539200	4.26080100
H	6.33969000	-2.68267200	4.75256600
H	10.51729800	2.63092700	-1.45641000
H	10.01802700	1.34304800	-2.62017500
H	8.33292000	7.78284400	-0.19738500
H	4.86079000	3.99716800	6.39790900
H	3.13818400	3.90234000	5.99980000
H	1.93778500	-0.21955800	6.96570200
H	0.14834800	-0.00777400	6.76394000
H	4.65040800	5.62650600	-2.94080300
H	0.51197200	3.73149600	-5.03045300
H	0.47567300	-1.42832000	-6.42424500
H	-4.27260200	-1.44102700	5.28855100
H	-2.73844700	-3.03999600	7.21643100
H	-2.44967000	-3.59423400	8.87324800
H	-2.35031100	-6.48551700	4.73827000

E:P_{Mg} (–3.1)

C	4.13449900	3.93465100	5.54822800
C	4.34158800	2.74626600	4.59459400
C	3.54448300	2.96525600	3.29128300
C	3.49709500	1.77216200	2.33629900
O	4.58090300	1.20945100	2.02012600
O	2.36996200	1.40223800	1.86869500
H	4.23010200	4.88271600	5.00695700
H	5.39795700	2.61495200	4.34980400
H	4.00896400	1.81968100	5.07986100
H	2.52404100	3.28628200	3.52162200
H	4.02962900	3.78866700	2.74939800
C	-10.24752700	3.65543200	-0.42190800
C	-8.80720200	3.31424000	-0.64890500
C	-7.84905700	3.20911300	0.33524400
C	-8.10186500	3.20300600	-1.90353800
C	-6.71958400	3.03609200	-1.59260000
C	-8.48876500	3.25078700	-3.25436100
N	-6.59734300	3.04119600	-0.22130600
C	-5.73661600	2.91910100	-2.58280000

C	-7.51568300	3.13951900	-4.24121000
C	-6.15322800	2.97748600	-3.90792100
H	-10.50652000	4.58489500	-0.94625300
H	-10.42325300	3.83801300	0.64392800
H	-7.97057700	3.26892100	1.40707500
H	-5.71494800	2.88240400	0.25715700
H	-9.53409200	3.37478000	-3.52259600
H	-4.69528400	2.78579800	-2.31499000
H	-7.80526400	3.17673400	-5.28700500
H	-5.41602000	2.89307100	-4.70112900
C	-7.99882700	-2.00817900	0.52205100
C	-7.62134000	-1.82919200	-0.92551000
C	-7.26322800	-2.93278300	-1.71164800
C	-7.60019700	-0.55665600	-1.50938200
C	-6.89204300	-2.76726800	-3.04459700
C	-7.21631600	-0.38476700	-2.84107600
C	-6.85751700	-1.49088400	-3.61098900
H	-8.37100200	-1.07178200	0.94771400
H	-7.12792300	-2.30813800	1.11838200
H	-7.27832300	-3.92861900	-1.27632900
H	-7.88991100	0.31009800	-0.92078600
H	-6.63717500	-3.63702900	-3.64275300
H	-7.20639800	0.60885500	-3.27221500
H	-6.55720400	-1.36091200	-4.64622300
C	7.85954200	7.55771600	0.72488200
C	7.64772300	6.12225400	1.15059800
C	8.38961200	5.57616300	2.20620000
C	6.68580900	5.29930700	0.54833500
C	8.16927600	4.28123300	2.66365500
C	6.45060700	3.99541200	0.99050400
C	7.18436500	3.48134400	2.07238900
O	6.98598900	2.24338100	2.58841300
H	8.43856000	8.09964900	1.47869100
H	6.90212800	8.08037900	0.61443700
H	9.14676300	6.18659700	2.69245700
H	6.10862800	5.68233500	-0.29119100
H	8.73971600	3.87118400	3.49039100
H	5.71667200	3.36609000	0.49516100
H	6.11579600	1.87719000	2.29976500
C	3.87572000	4.87865600	-2.67650600
C	2.95179100	4.63762500	-3.85360900
O	2.68289200	5.53362100	-4.65562000
C	3.10475700	5.64135200	-1.56382500
C	2.04504800	4.82089300	-0.91194500
C	2.04917100	4.13596100	0.27546600
N	0.84350500	4.47489100	-1.51325800
C	0.19194700	3.59717100	-0.70767400
N	0.89832400	3.38002900	0.39477400
H	4.31305200	3.95917400	-2.28035400
H	3.80982800	5.95081700	-0.78729900
H	2.68282100	6.55120400	-2.00509700
H	0.56398600	4.74134800	-2.44657700
H	2.81100300	4.13960200	1.03603300
H	-0.76168500	3.14456500	-0.93174200
N	2.33968900	3.42313400	-3.91098100
C	1.48632000	3.10014500	-5.05885600
C	1.28642700	1.58455400	-4.95682300
C	1.49141700	1.27676200	-3.46600500
C	2.60661700	2.24422500	-3.06377800
H	1.99876600	3.39305300	-5.98129500
H	0.31140600	1.27213800	-5.32407200
H	2.05405800	1.07022000	-5.54426200
H	0.57490300	1.48510500	-2.90361200
H	1.77212600	0.23771000	-3.27811600
H	2.57196500	2.50831000	-2.00434400
H	3.59783100	1.82942000	-3.27225100
C	-3.78415500	-3.12020600	-5.13216100
C	-2.61726600	-4.00478100	-4.77637100
O	-1.71335600	-4.28115500	-5.56815500
H	-3.45862700	-2.07847900	-5.10839400
H	-4.61967900	-3.21995900	-4.44256900
N	-2.61059400	-4.47064400	-3.49738000
C	-1.45802100	-5.21590300	-3.00961600
C	-1.81452300	-5.50909000	-1.55400400
C	-2.59585600	-4.25734500	-1.14978600
C	-3.44753000	-3.97153800	-2.38767800
H	-0.55719900	-4.58551800	-3.06816000
H	-2.46117300	-6.38755500	-1.49284100
H	-0.94036100	-5.69905000	-0.92891800
H	-3.20330900	-4.38201500	-0.25604300
H	-1.90571200	-3.42378200	-0.98121600
H	-4.39910600	-4.51637300	-2.34014200
H	-3.66972100	-2.90921700	-2.49658400
C	0.32068300	-1.61777500	-5.34798900
C	-0.88680000	-0.94112700	-4.75356300
C	-1.14441400	-1.10143400	-3.38295600
C	-1.77578100	-0.16286800	-5.50305900
C	-2.25161200	-0.51152600	-2.77523000
C	-2.88971400	0.43351700	-4.90066100
C	-3.13638500	0.25668800	-3.53866100
H	0.21659200	-2.69974800	-5.19370300
H	1.24051300	-1.29376400	-4.84928600
H	-0.46614300	-1.70821600	-2.78680800
H	-1.60097400	-0.03348300	-6.56772900
H	-2.43191300	-0.65205200	-1.71560600
H	-3.57574700	1.02185500	-5.50378800
H	-4.01626800	0.68972400	-3.07555000
C	9.70133200	1.86482700	-1.78295600
C	9.18309000	1.00096500	-0.64406100
O	9.92834800	0.44209200	0.15385200
H	8.91754300	2.51872100	-2.17938700
N	7.82526800	0.89018200	-0.60173100
C	7.21205700	-0.05383300	0.28708400
C	7.18550300	-1.48205000	-0.27016400
O	7.43236800	-1.75957500	-1.44263400
H	7.25515600	1.23882300	-1.37075000
H	7.72868600	-0.03397800	1.24917000
H	6.17468200	0.24929100	0.46054400
N	6.78759100	-2.41412400	0.63846000
C	6.36895700	-3.74231200	0.21262400
C	5.21577300	-3.69521600	-0.81690400
C	4.20058100	-2.67667800	-0.38074000
C	3.50159400	-2.56389400	0.79538500
N	3.92062100	-1.52665700	-1.10505900
C	3.09762500	-0.74879300	-0.40076800
N	2.81876000	-1.36675000	0.75199100
H	6.43898900	-2.08207000	1.53621200
H	6.01707000	-4.25071400	1.11721300
H	5.59543100	-3.43055800	-1.80265400
H	4.75740600	-4.68760800	-0.89891700
H	4.30454900	-1.26153900	-2.09922100
H	3.44854000	-3.19825600	1.66256500
H	2.73826600	0.22684000	-0.68159600
C	7.74248800	-3.76058800	-5.17347200
C	7.37354600	-2.36218600	-5.67553200
C	6.80329600	-1.42053700	-4.60517700
C	5.44573000	-1.75480400	-3.99848000
O	4.95851400	-2.90834400	-4.08554400
O	4.87653000	-0.79506000	-3.35133100
H	6.83172500	-4.24108900	-4.80324500
H	8.25757400	-1.87456500	-6.10576500
H	6.64829200	-2.45846500	-6.49346500
H	7.47654600	-1.35582800	-3.73987400
H	6.72728300	-0.40204300	-5.00494100
C	5.18333000	-2.58416000	4.82701400
C	4.87200500	-1.23566700	4.19120400
O	5.23782300	-1.28473600	2.82204900
H	4.87134400	-2.59991700	5.87633100
H	3.80094600	-1.00352500	4.28213100
H	5.41878000	-0.43731300	4.71454500
H	4.99088800	-0.42276100	2.42145900
C	1.06608300	-0.04350300	6.24869000

C	1.04945200	-1.03154600	5.08460800
C	0.71629000	-0.43030800	3.71897900
O	0.28593700	0.75697700	3.65357300
O	0.89263900	-1.17674800	2.70715700
H	1.24251200	0.95893800	5.84403400
H	2.00564800	-1.54907200	4.96860100
H	0.31875800	-1.82392500	5.25005900
C	-3.89539500	-1.13169700	4.29134800
C	-4.26030300	0.27703800	3.89821300
C	-5.56367900	0.57919700	3.48896700
C	-3.32215400	1.31266800	3.96002600
C	-5.92777800	1.88520600	3.16669600
C	-3.68560700	2.62074300	3.63979100
C	-4.98974200	2.91760500	3.23902400
H	-2.80948300	-1.24949800	4.36103400
H	-4.26069900	-1.85269700	3.55519000
H	-6.29934700	-0.21806900	3.41782700
H	-2.29711300	1.08497900	4.24179900
H	-6.94887500	2.08781400	2.86082600
H	-2.95081400	3.41559800	3.67858000
H	-5.25986500	3.93875800	2.98246200
C	-2.43728000	-3.80529200	7.81786100
C	-0.97021400	-4.09709900	7.49679600
C	-0.62256700	-4.05689300	6.00482800
C	-1.06276600	-5.16704800	5.09247000
O	-2.20016200	-5.77618100	5.42271500
O	-0.44372100	-5.44624800	4.06879300
H	-3.07156000	-4.67271300	7.62105400
H	-0.33926900	-3.33698300	7.97080400
H	-0.66626800	-5.05729300	7.92846200
H	-1.07352700	-3.15556300	5.56571500
H	0.45569900	-3.96668700	5.85489100
C	-1.96150100	-0.47307900	1.11657000
C	-2.21583100	0.88697600	0.87111000
C	-3.49170000	1.22688100	0.36051100
C	-4.43660900	0.25002700	0.06494800
C	-4.15872500	-1.09914700	0.30425600
C	-2.92279700	-1.44957600	0.84920500
O	-1.30391300	1.83771800	1.10660900
Mg	0.45133300	1.95217300	2.00643300
O	-0.05055900	3.53922300	3.26145700
O	1.49989200	-3.76458900	2.91406400
O	-2.03075600	4.28570700	1.57978500
O	5.91268800	1.60613000	-2.69875600
C	1.97069800	-8.38067800	-0.64420000
C	2.94477400	-7.24831300	-1.01108500
N	2.29230000	-5.98562700	-1.37811900
C	1.74528500	-5.13112700	-0.49628600
N	1.27947000	-3.95831400	-0.95118300
N	1.61420200	-5.45135100	0.79647400
H	1.23270900	-8.50502600	-1.44193500
H	1.41629100	-8.16776800	0.27481700
H	3.65982900	-7.06956700	-0.19587500
H	3.53866100	-7.54094400	-1.87907300
H	2.47163100	-5.57449500	-2.29831000
H	1.66271300	-3.63812700	-1.85359900
H	1.00093600	-3.26656200	-0.27146100
H	2.03993600	-6.30613500	1.11534400
H	1.48324300	-4.71409500	1.52503300
C	-3.51072400	-9.06532000	-0.43634600
C	-3.09113400	-7.94814200	0.47407100
C	-1.88672900	-7.81442000	1.11971300
C	-3.90381000	-6.83596300	0.90158800
C	-3.11949000	-6.06146500	1.80582800
C	-5.19843600	-6.39710700	0.57749500
N	-1.89769300	-6.69103400	1.92966200
C	-3.59532300	-4.87222800	2.37318900
C	-5.67498400	-5.21992200	1.14270100
C	-4.88052500	-4.46520300	2.02934000
H	-2.66178800	-9.48334900	-0.98470700
H	-4.25914200	-8.73921100	-1.16365100
H	-1.00992600	-8.44494000	1.07043000
H	-1.12820800	-6.33533800	2.48155800
H	-5.81609900	-6.96634300	-0.11095900
H	-2.97782300	-4.26604200	3.02807700
H	-6.67329600	-4.87055600	0.90139800
H	-5.27518800	-3.54291000	2.44256700
O	2.62422800	-3.85626900	-3.34836400
H	-4.89756300	-1.85474600	0.06023100
H	-5.39301600	0.55048800	-0.34680100
H	-2.70279100	-2.48831900	1.06749100
O	-3.82036400	2.55572100	0.11988600
H	-3.40701100	3.12494100	0.79430200
H	-1.87813600	4.93641100	0.87936100
H	-1.72526200	3.37888100	1.25183400
H	-0.42772900	3.19842900	4.08325700
H	-0.75697200	4.05759600	2.80047600
C	-3.88127200	7.81662100	2.05971900
C	-3.50089600	7.90274800	0.59233500
O	-3.12345100	8.93836000	0.06006600
C	-5.38911100	7.61418100	2.27819600
C	-6.25399000	8.59159200	1.48982600
O	-5.66802800	6.24834200	1.90619000
H	-3.37835100	6.95253300	2.51218400
H	-5.59310100	7.73294400	3.35187500
H	-6.60478900	6.16600000	1.68597200
H	-7.31689500	8.43197300	1.70451200
H	-6.00780700	9.62329100	1.75566500
H	-6.09432100	8.47430300	0.41408700
N	-3.63808900	6.70562900	-0.07030600
C	-3.73606700	6.68371700	-1.52529800
C	-2.94822100	5.51377600	-2.11353500
H	-4.21322500	6.02979200	0.42441600
H	-3.35998300	7.64246000	-1.88527100
H	-3.09630200	5.44438000	-3.19548500
H	-1.87842600	5.64582100	-1.91547900
H	-3.26518700	4.56482300	-1.66836000
H	-0.99674300	-0.75047000	1.52993100
H	2.21509100	-0.99088200	1.49635200
H	1.21458800	-2.81233300	2.88069600
H	0.84620800	-4.24856000	3.44882900
H	5.61836900	0.69389000	-2.95577100
H	6.34519900	1.95147500	-3.48902800
H	3.51622100	-3.43079500	-3.57131700
H	2.10087300	-3.78585600	-4.15727500
H	-3.50177700	8.71001900	2.56660200
H	-4.79511400	6.60761900	-1.79284100
H	-10.99943700	2.92185600	-0.73155700
H	-8.77162400	-2.76956500	0.67121100
H	-3.94434700	-9.86463400	0.17369100
H	-4.15313400	-3.32610000	-6.14234300
H	-1.22963300	-6.11582700	-3.59005000
H	2.48641500	-9.33792400	-0.51233700
H	8.12720100	-4.40455500	-5.97126500
H	8.43122500	-3.74541200	-4.32225400
H	7.25052000	-4.29717000	-0.12534500
H	4.65912800	-3.38276000	4.29235300
H	6.26779300	-2.73242300	4.78754200
H	10.52779200	2.49528900	-1.43840000
H	10.02132200	1.20422500	-2.59551400
H	8.38880800	7.67740700	-0.22626200
H	4.84752500	3.97310000	6.37851400
H	3.12599600	3.89256600	5.97267400
H	1.87993400	-0.21015900	6.96142300
H	0.09366600	0.01762900	6.74983800
H	4.69822400	5.53832200	-2.97234600
H	0.55119500	3.66978300	-5.06877800
H	0.47021200	-1.49915400	-6.42644200
H	-4.33404000	-1.38194400	5.26286600
H	-2.82522000	-2.98225900	7.20939600
H	-2.54991100	-3.52758500	8.87139900
H	-2.44063500	-6.39117800	4.70506400

The enzyme-substrate complex with a monodentate binding mode of the substrate to the Mg-enzyme of 2,3-DHBD_*Ao*(+2.8)

C	4.53921300	3.57006500	5.58059000
C	4.65878200	2.35018300	4.65572100
C	4.04201400	2.65140900	3.27769200
C	3.93340400	1.45095600	2.34118700
O	4.94085300	0.71389800	2.18204900
O	2.83236400	1.24804600	1.73416700
H	4.77728900	4.49023900	5.03451800
H	5.70449700	2.06565000	4.51729200
H	4.15097600	1.48815400	5.10654700
H	3.05481600	3.11145700	3.40037300
H	4.68576300	3.38858000	2.77841300
C	-9.55422300	5.05248000	-0.88065400
C	-8.20484300	4.39636600	-0.78674300
C	-7.60120000	3.92991400	0.35762600
C	-7.22113300	4.25815900	-1.83651700
C	-6.03921700	3.71697300	-1.24698700
C	-7.21223300	4.56113000	-3.20890400
N	-6.31207100	3.50335400	0.08704100
C	-4.86720800	3.49918000	-1.98070100
C	-6.05343000	4.33941800	-3.94460500
C	-4.89257400	3.81882600	-3.33506000
H	-9.51484700	5.89497900	-1.58007800
H	-9.84156800	5.46226500	0.09330400
H	-7.98719500	3.88741500	1.36572900
H	-5.65206000	3.20714300	0.79231400
H	-8.09904400	4.96731800	-3.68680500
H	-3.96936100	3.10468000	-1.51455600
H	-6.03718800	4.57322300	-5.00476400
H	-3.99886500	3.66205900	-3.93131700
C	-8.10497200	-0.83555600	0.24062000
C	-7.65934700	-0.73219700	-1.19285600
C	-7.42376000	-1.88471600	-1.95340700
C	-7.42743700	0.51981600	-1.77410200
C	-6.97002400	-1.78094000	-3.26688800
C	-6.95291600	0.62668700	-3.08284900
C	-6.72148900	-0.52682400	-3.83238000
H	-8.34987400	0.14809800	0.65178200
H	-7.32599400	-1.27356500	0.87360700
H	-7.59272100	-2.86358700	-1.51052500
H	-7.61605100	1.42013600	-1.19739400
H	-6.81305800	-2.68195900	-3.85225100
H	-6.77078600	1.60844400	-3.50733100
H	-6.35205600	-0.45276300	-4.85060000
C	8.86350800	6.56480900	0.82672300
C	8.50294000	5.17793900	1.30624500
C	9.13401500	4.62731000	2.42920900
C	7.50600700	4.41059100	0.68797400
C	8.77425300	3.38254800	2.93495100
C	7.13265700	3.15698300	1.17682100
C	7.75665300	2.64026900	2.32468800
O	7.41938100	1.45277400	2.88599000
H	9.46356000	7.08641600	1.57841500
H	7.96393200	7.16844800	0.65644000
H	9.91463500	5.19533500	2.92952800
H	7.01116300	4.79594000	-0.20188400
H	9.25937400	2.96987800	3.81326700
H	6.37790200	2.56623300	0.66656400
H	6.53711700	1.15733200	2.55992600
C	4.67208300	4.35848600	-2.66358600
C	3.75615200	4.21862500	-3.86183700
O	3.62488500	5.13225300	-4.67908900
C	4.02559900	5.31359600	-1.62065400
C	2.84698200	4.70721600	-0.95802600
C	2.72337700	4.08572800	0.25585100
N	1.64739800	4.46485900	-1.60701100
C	0.87090900	3.69157600	-0.80856800
N	1.49534200	3.45741100	0.34080800
H	4.95529200	3.40739300	-2.20899400
H	4.76348100	5.55695200	-0.85128400
H	3.76498700	6.24444000	-2.13614900
H	1.44492100	4.71508100	-2.56471200
H	3.44981100	4.03612300	1.04982300
H	-0.11070400	3.32458800	-1.06859900
N	2.99869600	3.09139000	-3.92542700
C	2.14650500	2.85739500	-5.09590500
C	1.74668500	1.38366500	-4.96747100
C	1.85174500	1.09103100	-3.46252200
C	3.06532900	1.91890600	-3.03129100
H	2.72268300	3.05721100	-6.00526900
H	0.75381700	1.18914400	-5.36649000
H	2.46235000	0.75847900	-5.51118500
H	0.94856700	1.42804700	-2.94197100
H	1.99018300	0.02986300	-3.24236100
H	3.01089100	2.22474500	-1.98367000
H	4.00288800	1.37232000	-3.17336300
C	-3.89446200	-2.61615400	-5.24311600
C	-2.85805300	-3.62461400	-4.82402000
O	-1.93374500	-3.98365000	-5.55746800
H	-3.43645800	-1.62610000	-5.23565500
H	-4.75955300	-2.58440400	-4.58583000
N	-2.97469700	-4.09208600	-3.55213500
C	-1.93534700	-4.95226700	-3.00442600
C	-2.42743300	-5.25616000	-1.58797500
C	-3.20577900	-3.99758100	-1.21505400
C	-3.91127600	-3.62223100	-2.51651300
H	-0.96923000	-4.43233700	-3.00697100
H	-3.10135500	-6.11655500	-1.60669700
H	-1.62039500	-5.48556000	-0.89007900
H	-3.89854300	-4.13741200	-0.38763500
H	-2.50633600	-3.20380700	-0.94512300
H	-4.87967400	-4.13323400	-2.60319500
H	-4.08878300	-2.54868600	-2.58942900
C	0.37800200	-1.67167100	-5.34243300
C	-0.77924500	-0.85268200	-4.83356000
C	-1.20335600	-1.02035000	-3.50610600
C	-1.46758600	0.06876300	-5.63130000
C	-2.28344100	-0.30305700	-2.99650100
C	-2.53895300	0.80854200	-5.11796000
C	-2.95639500	0.62189100	-3.79978400
H	0.15384100	-2.72937900	-5.16035100
H	1.30498800	-1.42527600	-4.81371600
H	-0.69048000	-1.73842400	-2.87042400
H	-1.16685900	0.20077500	-6.66720300
H	-2.60990900	-0.46634500	-1.97735200
H	-3.06058000	1.51445800	-5.75832200
H	-3.80561900	1.16873300	-3.40460900
C	10.01671300	0.62534700	-1.51499100
C	9.36284600	-0.13920600	-0.37673000
O	10.01001300	-0.77201000	0.45088200
H	9.33591000	1.36638000	-1.94655700
N	8.00142000	-0.07159200	-0.36771600
C	7.25432100	-0.91237800	0.52156200
C	7.03642000	-2.32758200	-0.02481200
O	7.28940600	-2.65793800	-1.18226300
H	7.49949100	0.32299900	-1.16170500
H	7.76794600	-0.95929200	1.48487200
H	6.27062400	-0.46446400	0.68919000
N	6.46992200	-3.17507600	0.87652500
C	5.91022100	-4.44975300	0.46158700
C	4.83305100	-4.30811000	-0.64210500
C	3.91944300	-3.17012700	-0.30073600
C	3.17383500	-2.94495200	0.82834400
N	3.84684200	-2.00144500	-1.04338800
C	3.10579400	-1.10631900	-0.38868400
N	2.68102500	-1.66338600	0.74710800

H	6.13323600	-2.78390800	1.75485100
H	5.45269200	-4.88679400	1.35560600
H	5.30119200	-4.12183400	-1.60766000
H	4.27137000	-5.24553100	-0.72400700
H	4.30078000	-1.79329500	-2.01683200
H	2.99372200	-3.55901900	1.69274000
H	2.90465400	-0.09404000	-0.69548400
C	7.44194800	-4.76697900	-4.87215000
C	7.30233400	-3.34394000	-5.41934900
C	6.83108700	-2.30316700	-4.39473600
C	5.41677000	-2.43262700	-3.84528000
O	4.78794400	-3.51631000	-3.91609300
O	4.94997900	-1.38483800	-3.25549800
H	6.45952300	-5.10357500	-4.52983400
H	8.26576400	-3.00245400	-5.81868200
H	6.60582100	-3.35516400	-6.26736300
H	7.46986800	-2.31525200	-3.50111000
H	6.91768200	-1.29389400	-4.81486600
C	4.74039800	-3.04437800	5.01118000
C	4.61349900	-1.68968600	4.32849100
O	4.94783000	-1.84949100	2.95938800
H	4.39650200	-2.99516700	6.04944000
H	3.58584500	-1.30808100	4.41950200
H	5.27532700	-0.95701100	4.81247500
H	4.90118000	-0.96083500	2.54205500
C	0.95072400	0.04621500	6.24392400
C	0.82519200	-0.96769000	5.11262500
C	0.85180800	-0.37195800	3.70932800
O	0.63825100	0.86334900	3.54273200
O	1.08462800	-1.19057500	2.77295300
H	1.26910600	1.00404800	5.82039400
H	1.61375100	-1.72455200	5.14337300
H	-0.11473700	-1.52204500	5.18268700
C	-4.04496300	-0.42335800	4.13440500
C	-4.23442900	1.01211700	3.71301600
C	-5.52293500	1.56030900	3.66854900
C	-3.15128000	1.83162700	3.37661000
C	-5.72543800	2.89332100	3.31286000
C	-3.35432200	3.16184300	3.00559600
C	-4.63931500	3.70721700	2.97473000
H	-2.98526600	-0.67478900	4.23872200
H	-4.47769900	-1.11110100	3.40509100
H	-6.37477400	0.93703600	3.92931500
H	-2.14604600	1.41570000	3.39698600
H	-6.73154800	3.30309800	3.31388600
H	-2.51740800	3.79210500	2.73967600
H	-4.78298200	4.74853800	2.69667100
C	-3.06770600	-3.18755500	7.75623300
C	-1.68202000	-3.71684000	7.39300600
C	-1.39535400	-3.72413900	5.87861900
C	-2.02245200	-4.75274200	4.96323800
O	-3.32607800	-4.97665200	5.13603200
O	-1.38965900	-5.28581800	4.05836500
H	-3.83360300	-3.94402300	7.56088900
H	-0.91382600	-3.07332800	7.83670700
H	-1.52148100	-4.71702200	7.81068400
H	-1.70948500	-2.75583200	5.46399200
H	-0.31994800	-3.81051400	5.70963700
C	-2.45246700	0.31912700	0.38218400
C	-2.04911500	-0.98347100	0.73293500
C	-3.01042400	-1.92309000	1.14011600
C	-4.36016200	-1.63462200	0.96357900
C	-4.75747000	-0.36955800	0.51667700
C	-3.81632600	0.60999400	0.27066400
O	-0.75661200	-1.39668600	0.70795500
O	-1.84304300	2.56893000	-0.03984100
O	-0.23755000	1.10331900	0.55652000
Mg	1.00829600	1.95224500	1.83913300
O	0.14426900	3.46541100	2.90951900
O	-0.08802800	-3.51316500	2.49920900
O	-1.28495600	4.90064300	1.15112600
O	6.25736200	0.85382300	-2.53705200
C	0.96763900	-8.48752200	-0.46599600
C	2.08853400	-7.46244400	-0.72512200
N	1.62100200	-6.15730100	-1.21505700
C	1.03832900	-5.22267300	-0.44246300
N	0.77821800	-4.01585800	-0.95953600
N	0.68835300	-5.49170600	0.81872600
H	0.30199500	-8.52490800	-1.33360600
H	0.34940200	-8.22493800	0.39817900
H	2.69927500	-7.31782900	0.17631000
H	2.76129800	-7.84409200	-1.49544600
H	1.95299900	-5.80544900	-2.11717500
H	1.28078100	-3.76035000	-1.81959400
H	0.35802500	-3.29896600	-0.37950600
H	0.84853000	-6.40589700	1.20352000
H	0.39706100	-4.72345800	1.44779900
C	-4.56025000	-8.44058400	-0.43674200
C	-4.52523500	-7.19059200	0.40802000
C	-3.51964700	-6.72305100	1.20859700
C	-5.67245300	-6.33877100	0.66374000
C	-5.28193800	-5.39295300	1.64443400
C	-6.97455900	-6.28786100	0.14410300
N	-3.95965900	-5.65750400	2.01316900
C	-6.15534200	-4.41602400	2.12364400
C	-7.84091300	-5.29600200	0.59622000
C	-7.43760600	-4.37212800	1.57899000
H	-3.56194600	-8.83556000	-0.63889200
H	-5.08670100	-8.28855200	-1.38266300
H	-2.49806400	-7.06509000	1.29854700
H	-3.34472800	-4.85180400	2.13558900
H	-7.30089100	-7.00582600	-0.60259700
H	-5.84785600	-3.72337100	2.90141200
H	-8.84943200	-5.24051000	0.19810400
H	-8.14009900	-3.62143600	1.92625200
O	2.33528100	-4.18196400	-3.23731900
H	-5.79980800	-0.13789200	0.38820500
H	-5.09317200	-2.39841200	1.19276000
H	-4.10677500	1.61314600	-0.00125900
C	-1.46640700	1.41413000	0.26527600
O	-2.60986300	-3.11078300	1.67933900
H	-1.63617000	-3.11255400	1.86511500
H	-0.59755100	5.32077300	0.61643700
H	-1.51332900	4.06419500	0.66254800
H	-0.35317700	3.21145500	3.69653300
H	-0.38312700	4.12283300	2.38297600
C	-2.77692500	8.39418100	1.74754800
C	-2.65202000	8.50412900	0.24816700
O	-2.69553800	9.56519300	-0.36788400
C	-4.25377500	8.25768900	2.19216200
C	-5.13565500	9.41573900	1.73519700
O	-4.79396800	6.99622500	1.78134300
H	-2.26271700	7.49129500	2.09773800
H	-4.25980800	8.20661600	3.28615700
H	-4.87603900	7.00543300	0.81739400
H	-6.16103700	9.26207700	2.08186000
H	-4.76613400	10.36635500	2.13140700
H	-5.13614500	9.50193200	0.64406400
N	-2.58097000	7.28734600	-0.36645100
C	-2.66747200	7.17342700	-1.80979000
C	-2.01706300	5.88393600	-2.29879600
H	-2.56626000	6.44762300	0.20114700
H	-2.17534800	8.05150800	-2.23592400
H	-2.12418900	5.78718600	-3.38250500
H	-0.94810100	5.88054000	-2.05612100
H	-2.47982700	5.00393200	-1.84296100
H	-0.22655900	-0.55886000	0.67059900
H	2.12433100	-1.20640500	1.49044900
H	0.39864000	-2.66630300	2.70805700
H	-0.28040700	-4.03718000	3.29852100
H	5.86092000	-0.01320300	-2.81493800
H	6.75738800	1.14696000	-3.30842600

H	3.27279500	-3.85947700	-3.43584000
H	1.86855800	-4.12612300	-4.08122600
H	-2.29934400	9.24092900	2.25150400
H	-3.71818500	7.23125200	-2.11259900
H	-10.38595100	4.41739100	-1.20301300
H	-8.97596000	-1.48513300	0.37618200
H	-5.11493500	-9.16270300	0.17175000
H	-4.25478100	-2.79401300	-6.26172800
H	-1.80906100	-5.88691500	-3.56061100
H	1.34755900	-9.50086100	-0.29914400
H	7.76338600	-5.47343800	-5.64459300
H	8.09873100	-4.82385000	-3.99782100
H	6.72112500	-5.12300100	0.16467000
H	4.13238100	-3.77875700	4.47264400
H	5.79615000	-3.33493200	5.01172700
H	10.90762400	1.14908700	-1.15303600
H	10.27246900	-0.08935900	-2.30417500
H	9.43420600	6.59282300	-0.10738100
H	5.22393200	3.53256200	6.43431300
H	3.52084200	3.67014300	5.97029000
H	1.71266200	-0.21051100	6.98724500
H	-0.02011100	0.24570100	6.70929000
H	5.58364900	4.89752800	-2.94211700
H	1.29570800	3.54471800	-5.14842400
H	0.57588100	-1.59729600	-6.41692700
H	-4.54405400	-0.59216200	5.09432400
H	-3.32303200	-2.33367200	7.11995200
H	-3.17583700	-2.87335600	8.79979600
H	-3.62365900	-5.54041300	4.38930800

The transition states and intermediates involved in the reaction pathway of the Mn-enzyme of 2,3-DHBD_*Ao*

E:S$_{Mn}$ (0.0)

C	-4.28288100	-3.76523100	5.61434400
C	-4.46759300	-2.58672200	4.65290400
C	-3.61052600	-2.79181900	3.38424400
C	-3.59321900	-1.57004500	2.48210300
O	-4.67730200	-1.13613800	2.01497600
O	-2.47138600	-0.99269000	2.24122500
H	-4.38276400	-4.71943200	5.08576100
H	-5.51395500	-2.47154900	4.36054200
H	-4.17014100	-1.65488100	5.15188100
H	-2.58368600	-3.06502200	3.64265200
H	-4.05551900	-3.62396200	2.82487200
C	10.15601100	-3.75907800	-0.22302300
C	8.73056400	-3.51233300	-0.58952800
C	7.68011000	-3.53514200	0.30022900
C	8.14107400	-3.40952000	-1.90254800
C	6.72717300	-3.36907100	-1.72056100
C	8.65662200	-3.35984700	-3.20944500
N	6.47664600	-3.46732200	-0.37038100
C	5.83651100	-3.25162900	-2.79410800
C	7.77491600	-3.25805900	-4.27960500
C	6.37936900	-3.19898100	-4.07315000
H	10.48917500	-4.72661900	-0.62431900
H	10.25161900	-3.82915200	0.86632000
H	7.70268400	-3.61772500	1.37830100
H	5.55570500	-3.33643700	0.04590600
H	9.72886100	-3.39419200	-3.37930800
H	4.76556600	-3.19898200	-2.63017400
H	8.16276100	-3.21532900	-5.29269100
H	5.71717200	-3.10768100	-4.92904300
C	7.94306400	1.94513600	0.54364000
C	7.65086400	1.67957800	-0.91150500
C	7.25038800	2.72435700	-1.75638500
C	7.72492500	0.38492000	-1.43961300
C	6.92223200	2.47821800	-3.08766700
C	7.38072700	0.13135100	-2.76916800
C	6.97272200	1.17800700	-3.59534400
H	8.27720500	1.03516700	1.05045100
H	7.04847200	2.30075300	1.06731900
H	7.18281800	3.73526900	-1.36423300
H	8.04510200	-0.43894600	-0.80606600
H	6.62911600	3.30358400	-3.72899500
H	7.43722000	-0.87864400	-3.15502800
H	6.69832100	0.98347600	-4.62762500
C	-7.99086300	-7.49121000	0.85676200
C	-7.77850000	-6.04814600	1.24932500
C	-8.50465400	-5.48411800	2.30597700
C	-6.82916100	-5.23633400	0.61351500
C	-8.28007500	-4.18001600	2.73406700
C	-6.58921500	-3.92436500	1.02649900
C	-7.30686000	-3.39247300	2.10983200
O	-7.09974500	-2.14284400	2.59671700
H	-8.57746100	-8.01389800	1.61817700
H	-7.03407900	-8.01918900	0.76706200
H	-9.25214100	-6.08684600	2.81596200
H	-6.26793000	-5.63476700	-0.22948800
H	-8.83792800	-3.75397100	3.56125300
H	-5.86740300	-3.30196600	0.50513200
H	-6.23316000	-1.79562800	2.28573100
C	-3.95437600	-4.93747100	-2.57880500
C	-2.97304100	-4.75360700	-3.72091600
O	-2.62425500	-5.70101800	-4.42903800
C	-3.24233000	-5.62632200	-1.38395200
C	-2.19825400	-4.75004300	-0.78472100
C	-2.24991600	-3.87997600	0.27411900
N	-0.96897100	-4.51122700	-1.37954700
C	-0.34083000	-3.52147600	-0.70031600
N	-1.09457800	-3.12229100	0.31815100
H	-4.40038700	-3.99379600	-2.25499700
H	-3.97723200	-5.86418500	-0.61027300
H	-2.81177200	-6.57029800	-1.73468800
H	-0.65030900	-4.93579600	-2.23975300
H	-3.05034900	-3.75135800	0.98202700
H	0.63282500	-3.12541900	-0.94040400
N	-2.41079300	-3.52246000	-3.84957500
C	-1.52895600	-3.24254500	-4.98550300
C	-1.32380800	-1.72475900	-4.93881100
C	-1.58907300	-1.35053000	-3.47369100
C	-2.71524000	-2.30563600	-3.07244500
H	-2.02623200	-3.56102600	-5.90889500
H	-0.33188500	-1.43392800	-5.27732600
H	-2.06102000	-1.23056400	-5.57971300
H	-0.69527500	-1.52548700	-2.86574500
H	-1.88498400	-0.30686400	-3.34719900
H	-2.72235400	-2.52004300	-2.00217400
H	-3.69760300	-1.90518000	-3.33963700
C	3.79044800	2.93327700	-5.17906900
C	2.61584500	3.82334200	-4.86559500
O	1.71481100	4.06358200	-5.67237000
H	3.47128600	1.89085900	-5.12843700
H	4.61978400	3.05782500	-4.48638400
N	2.60222600	4.33029300	-3.60496900
C	1.46077100	5.10395200	-3.13716500
C	1.85742400	5.48799900	-1.71173800
C	2.66049800	4.27208600	-1.25499800
C	3.47761800	3.91248700	-2.49331500
H	0.55247700	4.48350500	-3.14637700
H	2.49255800	6.37729800	-1.72881400
H	1.00240700	5.70991500	-1.07166600
H	3.29151100	4.45855300	-0.38965600
H	1.97679300	3.45742600	-1.01254400
H	4.42682100	4.46469500	-2.51472100
H	3.70432400	2.84583900	-2.53993700

C	-0.32432200	1.45653300	-5.39295300
C	0.90408000	0.76409500	-4.85789100
C	1.25960800	0.89272400	-3.50602400
C	1.72897900	-0.01174900	-5.68269700
C	2.40033200	0.27064700	-2.99728900
C	2.86874500	-0.64432800	-5.17660800
C	3.21323000	-0.50141100	-3.83219200
H	-0.21441400	2.53781700	-5.24837100
H	-1.23375300	1.13204300	-4.87833300
H	0.64361700	1.49897000	-2.84560800
H	1.47765600	-0.11338900	-6.73502500
H	2.66533600	0.39779900	-1.95594200
H	3.49592700	-1.23319200	-5.84032600
H	4.11509400	-0.96030800	-3.43934300
C	-9.76426000	-1.85574100	-1.82375700
C	-9.23926500	-0.97543700	-0.70197600
O	-9.97739600	-0.40644500	0.09504600
H	-8.98566300	-2.52822600	-2.19919300
N	-7.87977000	-0.86648800	-0.67072300
C	-7.26361600	0.09581000	0.19596600
C	-7.21644300	1.50677200	-0.40178600
O	-7.41397700	1.74431900	-1.59208700
H	-7.31834200	-1.20211300	-1.45248400
H	-7.78719000	0.10557400	1.15436800
H	-6.22894100	-0.21001900	0.38200100
N	-6.85449000	2.46515800	0.49256700
C	-6.40711400	3.77868000	0.04915100
C	-5.24106900	3.71108900	-0.96951800
C	-4.27046400	2.64579100	-0.54540400
C	-3.72150400	2.39102200	0.68792900
N	-3.94701300	1.55506500	-1.33795700
C	-3.25664900	0.67480800	-0.61684500
N	-3.10604600	1.16236200	0.61636800
H	-6.56277000	2.15988800	1.41767600
H	-6.06701300	4.30099300	0.95008800
H	-5.61623400	3.47438900	-1.96459400
H	-4.75184800	4.68935000	-1.02414300
H	-4.27154000	1.31271800	-2.35605600
H	-3.77680900	2.93809900	1.61124200
H	-2.89268200	-0.28039900	-0.95510900
C	-7.72960000	3.65936000	-5.34849800
C	-7.38399200	2.25073500	-5.83383700
C	-6.79128200	1.32857800	-4.76267900
C	-5.43186400	1.69240600	-4.18048100
O	-4.98050600	2.86081800	-4.23774900
O	-4.81922500	0.73485200	-3.56814300
H	-6.81390000	4.13417200	-4.98638800
H	-8.28106600	1.76200600	-6.23494300
H	-6.67830800	2.32866800	-6.67081300
H	-7.45220900	1.27101400	-3.88716100
H	-6.70765900	0.30627500	-5.15032400
C	-5.27415500	2.73899100	4.70482600
C	-4.99073800	1.35968300	4.12852300
O	-5.47425100	1.32732400	2.79678000
H	-4.97078800	2.79234200	5.75565900
H	-3.91260400	1.13888100	4.14130900
H	-5.48462200	0.59115800	4.74149700
H	-5.22776700	0.46017900	2.41167000
C	-1.19009300	0.20717100	6.23456400
C	-1.19352200	1.30790800	5.17930800
C	-0.72514900	0.83195300	3.83454100
O	-0.07776700	-0.20459100	3.67427600
O	-1.09680100	1.63037500	2.84577300
H	-1.38059400	-0.76502000	5.77302200
H	-2.18995900	1.73356700	5.03550200
H	-0.55793300	2.14681300	5.47638900
C	3.79773100	1.20395500	4.29601700
C	4.18875700	-0.19803000	3.89829900
C	5.49470700	-0.48396300	3.48132900
C	3.26684200	-1.24885500	3.96387200
C	5.87725700	-1.78650700	3.16438900

C	3.65060500	-2.55654800	3.66251000
C	4.95948300	-2.83461500	3.26423600
H	2.71006500	1.29958200	4.37221000
H	4.14422700	1.93590700	3.55980500
H	6.21692400	0.32426900	3.39909600
H	2.24067000	-1.02898300	4.24692800
H	6.89398700	-1.97843400	2.83473100
H	2.92887100	-3.36371000	3.71163700
H	5.24722200	-3.85559400	3.02694800
C	2.32824800	3.98556700	7.73335700
C	0.88266900	4.30143000	7.34288500
C	0.63057600	4.42133200	5.82404000
C	1.21981800	5.61107400	5.10550500
O	2.56681300	5.64000100	5.16722200
O	0.57564200	6.43767000	4.48719400
H	2.98619000	4.83284400	7.52640000
H	0.22455600	3.50155000	7.70368500
H	0.54438700	5.21794600	7.84032100
H	1.04130500	3.53893800	5.31749900
H	-0.44232500	4.45980100	5.62395200
C	2.16585200	0.41755200	0.56921500
C	2.35893500	-0.99162900	0.62284800
C	3.66540100	-1.47530300	0.33358200
C	4.71876600	-0.62875400	0.05144900
C	4.52654900	0.76026500	0.07713800
C	3.27322500	1.26545100	0.34557200
O	1.42124400	-1.87666900	0.89958500
O	0.74980500	2.31465700	0.47079800
O	-0.23110800	0.37527000	0.87139700
Mn	-0.44701900	-1.56007400	1.75215300
O	-0.01559100	-3.14531100	3.29314800
O	0.45624000	4.04870500	2.48585300
O	1.85800500	-4.33281500	1.69904900
O	-5.97019900	-1.57295700	-2.76988600
C	-1.96494500	8.35841100	-0.89272300
C	-2.92157500	7.15317700	-0.87893400
N	-2.30047200	5.88596100	-1.28290300
C	-1.56599500	5.09162400	-0.47790400
N	-1.21244300	3.88297200	-0.90985300
N	-1.21393900	5.50853700	0.74938600
H	-1.41875400	8.37722000	-1.84075400
H	-1.21325400	8.29801900	-0.09814500
H	-3.38302900	7.03092200	0.10897200
H	-3.73786200	7.32516700	-1.58405500
H	-2.57142300	5.45058200	-2.16938800
H	-1.64446400	3.55290300	-1.77666600
H	-0.59687500	3.27093000	-0.36190400
H	-1.29240600	6.48655800	0.97021000
H	-0.59006700	4.94894000	1.35175400
C	3.51900400	9.00738600	-0.65075600
C	3.12285300	7.90940500	0.29951600
C	1.95346800	7.80527900	1.01566500
C	3.94034700	6.79463100	0.70751400
C	3.19131200	6.04243500	1.66095200
C	5.21963900	6.34326200	0.34039800
N	1.98255200	6.68410500	1.82831100
C	3.68211300	4.85860600	2.22802900
C	5.71493800	5.17724600	0.91192500
C	4.95148300	4.44170600	1.84223900
H	2.65942000	9.40668000	-1.19632500
H	4.26037200	8.66802200	-1.37864400
H	1.09283000	8.46011900	1.01924500
H	1.27443900	6.41886600	2.50146800
H	5.81275500	6.89750400	-0.38124100
H	3.08684800	4.28407500	2.92755600
H	6.70632500	4.82576900	0.64630700
H	5.36150700	3.52914400	2.26401000
O	-2.68618600	3.85544900	-3.33201600
H	5.34797200	1.42369000	-0.14799600
H	5.69225800	-1.04298800	-0.17683300
H	3.10653400	2.33159500	0.35982700

C	0.84686300	1.06550200	0.63162900
O	3.86145200	-2.84762400	0.28975200
H	3.41076900	-3.27233300	1.04243100
H	1.84926300	-5.07190700	1.07191500
H	1.66436500	-3.48888200	1.20227300
H	0.45310700	-2.73677800	4.03285200
H	0.60915000	-3.78254700	2.87102800
C	3.73413900	-7.80219600	2.31058000
C	3.33468000	-7.90988100	0.84927500
O	2.90196400	-8.93792800	0.34807000
C	5.24919100	-7.62823700	2.50686400
C	6.08102300	-8.65018800	1.74000500
O	5.56079900	-6.28036100	2.09107900
H	3.25389000	-6.91964100	2.75220100
H	5.46229400	-7.71803000	3.58116400
H	6.49116400	-6.23791300	1.83495900
H	7.14987400	-8.50916000	1.93692600
H	5.81461900	-9.66657800	2.04259800
H	5.91108000	-8.56469300	0.66270200
N	3.52218200	-6.73572000	0.15160900
C	3.63195500	-6.76675700	-1.30487800
C	2.85895500	-5.61007800	-1.93841000
H	4.14897200	-6.08872700	0.62286700
H	3.24945400	-7.73332300	-1.63587300
H	3.01337700	-5.57841900	-3.02115400
H	1.78660300	-5.72732100	-1.74337100
H	3.17877900	-4.65098300	-1.51721800
H	-0.80477200	1.19850500	1.94338800
H	-2.77018700	0.56227100	1.38311100
H	0.87498900	3.49175000	1.79238400
H	-0.20218500	3.43586200	2.85252100
H	-5.62908100	-0.68756900	-3.06286100
H	-6.40271500	-1.93136900	-3.55437600
H	-3.54765500	3.42530200	-3.62597100
H	-2.14264800	3.89067200	-4.12983800
H	3.34359000	-8.67828300	2.83822000
H	4.69385200	-6.70674300	-1.56500300
H	10.91669500	-3.03989500	-0.54547300
H	8.72034200	2.70467800	0.67925500
H	3.95412800	9.81943500	-0.05896500
H	4.17040800	3.10843900	-6.19096400
H	1.24527600	5.98953000	-3.74431900
H	-2.47429600	9.32286400	-0.79159200
H	-8.10227800	4.28400700	-6.16713800
H	-8.42693000	3.67291800	-4.50365700
H	-7.28116700	4.33092100	-0.31240400
H	-4.73840000	3.51872600	4.15340800
H	-6.35673800	2.89455500	4.65052500
H	-10.59914900	-2.47007300	-1.47004800
H	-10.07171600	-1.21525900	-2.65720600
H	-8.51210200	-7.63299700	-0.09575600
H	-5.00403800	-3.77534100	6.43865300
H	-3.27825000	-3.71901800	6.04719200
H	-2.00948700	0.39994600	6.93534600
H	-0.22319400	0.15271900	6.74668900
H	-4.77942200	-5.59777100	-2.86372200
H	-0.59839400	-3.81962600	-4.97115400
H	-0.46392900	1.30941900	-6.46910100
H	4.22922000	1.47728500	5.26459400
H	2.71452500	3.14155200	7.15216600
H	2.42796600	3.73414000	8.79556100
H	2.87144800	6.37829300	4.61105000

$TS1_{Mn}$ (+17.8)

C	4.10900400	4.03700800	5.46869800
C	4.33596200	2.80356400	4.58804000
C	3.62479300	2.96338500	3.22628800
C	3.58483100	1.64978000	2.46389900
O	4.65965800	1.18018200	2.00534500
O	2.46669700	1.03504800	2.35830800
H	4.23034400	4.95997500	4.89147300
H	5.40065700	2.63525700	4.40712900
H	3.95124000	1.91192700	5.09971700
H	2.60738600	3.33857300	3.37064500
H	4.19109900	3.69872500	2.64327200
C	-10.17648800	3.77947900	-0.73035000
C	-8.74143900	3.38623800	-0.85647900
C	-7.86880800	3.23090700	0.19544100
C	-7.95003300	3.25857800	-2.05651500
C	-6.60382000	3.02933800	-1.64493300
C	-8.23188800	3.33999100	-3.43151000
N	-6.58956300	3.00815000	-0.26794300
C	-5.55531100	2.88351600	-2.56113100
C	-7.19208400	3.20441000	-4.34463900
C	-5.86679400	2.98035500	-3.91272400
H	-10.38008700	4.68011600	-1.32421900
H	-10.40377600	4.03334000	0.31083800
H	-8.06929300	3.28443000	1.25541600
H	-5.74941100	2.86230100	0.28077300
H	-9.24828700	3.50960800	-3.77511900
H	-4.54367100	2.69916500	-2.22049100
H	-7.39974500	3.26917000	-5.40832300
H	-5.07494900	2.87590300	-4.64866600
C	-8.01483400	-1.88501400	0.39510700
C	-7.59106700	-1.74900600	-1.04317500
C	-7.22370700	-2.87610800	-1.79036300
C	-7.53537600	-0.49075300	-1.65566700
C	-6.81578100	-2.74681100	-3.11641700
C	-7.11143900	-0.35554500	-2.97963000
C	-6.74846100	-1.48517600	-3.71256600
H	-8.38795900	-0.93281800	0.78401500
H	-7.16787800	-2.18005100	1.02768500
H	-7.25909400	-3.85982500	-1.32969900
H	-7.83538100	0.39312600	-1.09830800
H	-6.55614900	-3.63380800	-3.68593600
H	-7.07670000	0.62741700	-3.43397100
H	-6.41822500	-1.38548100	-4.74187700
C	7.95737000	7.48930400	0.61687400
C	7.73566300	6.07022500	1.08562100
C	8.42670400	5.57323800	2.19812500
C	6.81188400	5.21630400	0.46728400
C	8.19361200	4.29495200	2.69412600
C	6.56477100	3.92862000	0.94740200
C	7.24713200	3.46396400	2.08415800
O	7.03061300	2.24451100	2.63440300
H	8.52323500	8.05562500	1.36271300
H	7.00305500	8.00968900	0.47192200
H	9.15350900	6.20954900	2.69727000
H	6.27615200	5.56087500	-0.41541900
H	8.72475700	3.92228700	3.56363800
H	5.86235900	3.27298500	0.44079900
H	6.18357300	1.86237800	2.30343000
C	3.99598800	4.77226200	-2.78063700
C	3.06585600	4.52217800	-3.95203700
O	2.77976500	5.41708000	-4.74973600
C	3.23438200	5.54561900	-1.66999900
C	2.19052800	4.70909400	-1.01459600
C	2.22505400	3.99024600	0.15273900
N	0.99451400	4.34878000	-1.61728400
C	0.37599400	3.42720200	-0.83774900
N	1.09822400	3.19778600	0.25095700
H	4.42353200	3.85220500	-2.37525000
H	3.94275000	5.86318600	-0.89994500
H	2.80099700	6.44862500	-2.11355200
H	0.70618900	4.63023200	-2.54373900
H	2.99271300	3.99235900	0.90799500
H	-0.56374800	2.95328100	-1.07445700
N	2.46740600	3.30080800	-4.00599500
C	1.62346300	2.96235300	-5.15621400
C	1.41635800	1.44810800	-5.03784200

C	1.64706500	1.14347600	-3.55015700
C	2.75529000	2.12390800	-3.16203000
H	2.15061900	3.23491100	-6.07736700
H	0.43303700	1.13925700	-5.38555300
H	2.16886800	0.92484100	-5.63633200
H	0.73731700	1.33457000	-2.97126100
H	1.94872000	0.10934000	-3.37092500
H	2.73001500	2.38645700	-2.10247500
H	3.74802900	1.71906100	-3.37979200
C	-3.72305400	-3.19321200	-5.15825200
C	-2.57141400	-4.08059300	-4.76137700
O	-1.65424700	-4.37678100	-5.53067700
H	-3.38649300	-2.15490100	-5.15583000
H	-4.57099200	-3.26742700	-4.48169100
N	-2.59538900	-4.52045400	-3.47496700
C	-1.45827400	-5.26178600	-2.94462300
C	-1.84349900	-5.51908200	-1.49036200
C	-2.63874100	-4.26164100	-1.13432600
C	-3.46193900	-4.00735400	-2.39689900
H	-0.55230900	-4.64101100	-3.00298700
H	-2.48309100	-6.40174900	-1.41907700
H	-0.98020200	-5.68117500	-0.84329100
H	-3.26905100	-4.37543900	-0.25460000
H	-1.94893200	-3.43100900	-0.96146600
H	-4.41103700	-4.55831300	-2.36402800
H	-3.68976000	-2.94930800	-2.53370200
C	0.40349300	-1.74675700	-5.34429900
C	-0.80198700	-1.04773700	-4.77072200
C	-1.08307000	-1.19834700	-3.40210400
C	-1.67078000	-0.26258900	-5.53734500
C	-2.19843600	-0.59629700	-2.82083000
C	-2.78753900	0.35016000	-4.95767400
C	-3.06240000	0.17970700	-3.60009600
H	0.28831500	-2.82256400	-5.16228200
H	1.32127400	-1.42594600	-4.84060600
H	-0.42529700	-1.80503300	-2.78477400
H	-1.47834400	-0.14188400	-6.59995100
H	-2.40729500	-0.74810400	-1.76712500
H	-3.45685600	0.94229200	-5.57564600
H	-3.95040200	0.62065100	-3.15983200
C	9.76791100	1.71181200	-1.71408200
C	9.21050200	0.89566200	-0.56059200
O	9.92597900	0.37460200	0.28840000
H	9.00258500	2.36661400	-2.14427600
N	7.85121600	0.78686000	-0.56350700
C	7.20887500	-0.12369100	0.33928400
C	7.16940400	-1.56405700	-0.18118300
O	7.41377100	-1.87106800	-1.34720200
H	7.31258400	1.07401400	-1.37967000
H	7.71213300	-0.08399100	1.30778500
H	6.17329500	0.19683900	0.48722300
N	6.76239000	-2.46588700	0.75099800
C	6.33313900	-3.80130800	0.36851300
C	5.21564300	-3.79987200	-0.70089500
C	4.19866000	-2.75916100	-0.34512400
C	3.59791500	-2.49727600	0.86038600
N	3.83993300	-1.71875500	-1.18846700
C	3.06072600	-0.86407800	-0.52600400
N	2.90291200	-1.32105200	0.71757700
H	6.43415000	-2.10885000	1.64603200
H	5.95920400	-4.27484000	1.28262900
H	5.63090300	-3.57979000	-1.68386800
H	4.76183600	-4.79670000	-0.75288300
H	4.19858400	-1.51272200	-2.19152300
H	3.62782400	-3.01428600	1.80046300
H	2.61570800	0.03563700	-0.91192200
C	7.79349300	-3.97482400	-4.99193100
C	7.46506900	-2.58924600	-5.55246900
C	6.83806300	-1.61834000	-4.54374900
C	5.45196600	-1.95632800	-4.00744100
O	5.00035800	-3.12523600	-4.07658500
O	4.82119500	-0.98901300	-3.43572900
H	6.86676500	-4.42791200	-4.63051700
H	8.37516600	-2.11970800	-5.94722600
H	6.78526700	-2.70582300	-6.40612000
H	7.46429000	-1.52391500	-3.64632500
H	6.77487200	-0.61359500	-4.97908900
C	5.08754700	-2.51042300	4.93153000
C	4.82906400	-1.17310600	4.25795200
O	5.26522600	-1.26507900	2.91303200
H	4.75599300	-2.49536200	5.97474200
H	3.76039700	-0.92085100	4.29382800
H	5.36652200	-0.37445500	4.79121200
H	5.04154700	-0.41764400	2.46749300
C	0.97946200	0.11585800	6.21943700
C	0.94158800	-0.79867000	4.99597900
C	-0.10104200	-0.32843900	3.98968200
O	-0.17271000	0.89860900	3.71760200
O	-0.84846400	-1.21192000	3.43542900
H	1.16660400	1.12819300	5.83894800
H	1.89102700	-0.73358700	4.45443300
H	0.78352900	-1.84611700	5.23383700
C	-3.96251700	-0.96218200	4.20828600
C	-4.37924500	0.42818000	3.79254400
C	-5.71017700	0.68934500	3.44447300
C	-3.47081600	1.49270700	3.78652100
C	-6.12783700	1.97872400	3.11753900
C	-3.88532800	2.78539600	3.45670200
C	-5.21674000	3.03867300	3.12259600
H	-2.88248000	-1.05700200	4.29880800
H	-4.31226300	-1.71236300	3.49333000
H	-6.42789000	-0.12742100	3.43252700
H	-2.42751600	1.30742600	4.01967600
H	-7.16967900	2.15059100	2.86660000
H	-3.16448800	3.59555800	3.43136300
H	-5.52634900	4.04646800	2.85823400
C	-2.59559800	-3.56140600	7.82522200
C	-1.12826400	-3.88316000	7.52503200
C	-0.72970400	-3.77357600	6.05042500
C	-1.23668900	-4.77155100	5.04590300
O	-2.33037500	-5.45039500	5.39586000
O	-0.69891700	-4.91853000	3.95238000
H	-3.23991100	-4.42346300	7.63804900
H	-0.48677300	-3.17320700	8.05928200
H	-0.86970800	-4.87547100	7.91123000
H	-1.04982700	-2.80390900	5.64629600
H	0.35867900	-3.79450400	5.94428400
C	-1.85625800	-0.48850300	1.07965300
C	-2.23435600	0.90890000	0.87833100
C	-3.57749900	1.19286400	0.45837200
C	-4.49003700	0.19793300	0.22890100
C	-4.14272100	-1.16656900	0.44058400
C	-2.87913900	-1.49181400	0.83503400
O	-1.44462500	1.90531200	1.04315000
O	-0.25072200	-2.12477400	0.42139500
O	0.45276300	-0.00440600	0.60136400
Mn	0.55386500	1.75570300	1.83032000
O	0.03599100	3.61260700	3.08532300
O	1.12181200	-3.28433700	2.52681400
O	-2.01481300	4.51537400	1.36667900
O	5.99295000	1.35379300	-2.76011300
C	1.89156000	-8.40620800	-0.44209900
C	2.89928000	-7.30036000	-0.80752300
N	2.29434000	-6.05349500	-1.28099500
C	1.70426500	-5.13502800	-0.48715400
N	1.31408600	-3.98198700	-1.02089600
N	1.47560500	-5.40019400	0.81234000
H	1.17545900	-8.53659800	-1.25871600
H	1.31714400	-8.16293600	0.45652600
H	3.56745000	-7.09266200	0.04059800
H	3.54097900	-7.64811400	-1.61977800
H	2.51726600	-5.71014600	-2.21859600

H	1.67886800	-3.76226500	-1.95458000
H	0.83977000	-3.26199700	-0.46541700
H	1.89916900	-6.22730100	1.19895500
H	1.32720000	-4.60677100	1.46960200
C	-3.60094400	-9.01854200	-0.30865200
C	-3.20774000	-7.86412900	0.56991600
C	-1.99732000	-7.66646800	1.18881600
C	-4.04842600	-6.76210000	0.97126000
C	-3.27262600	-5.92861800	1.83017900
C	-5.36075200	-6.37297600	0.65464100
N	-2.03111000	-6.51621800	1.95920500
C	-3.76972600	-4.72822500	2.35473500
C	-5.86203000	-5.18780400	1.18209700
C	-5.07348100	-4.37428000	2.02115100
H	-2.73471600	-9.45401500	-0.81446600
H	-4.32638700	-8.71881700	-1.07010300
H	-1.09862900	-8.26490000	1.13843000
H	-1.25506000	-6.07838100	2.43870300
H	-5.97404900	-6.98698100	0.00158000
H	-3.15195400	-4.07464100	2.96131700
H	-6.87640800	-4.88040900	0.94879300
H	-5.48795000	-3.44975800	2.41055300
O	2.67177300	-4.13218700	-3.42869400
H	-4.88195100	-1.93707900	0.25441500
H	-5.48481000	0.45759600	-0.11480500
H	-2.58597100	-2.52287900	0.96886100
C	-0.43947300	-0.89908300	0.64183000
O	-3.90325600	2.50875200	0.22526300
H	-3.42065600	3.07795200	0.85253600
H	-2.08295000	5.20708700	0.68755200
H	-1.71024900	3.68568900	0.94179500
H	-0.41747300	3.18682200	3.82667100
H	-0.63644500	4.14315200	2.60753300
C	-3.79941500	7.92520800	1.74981100
C	-3.35661700	7.94550200	0.29830700
O	-2.88934700	8.93462600	-0.24597800
C	-5.31969900	7.77008900	1.91764300
C	-6.12719800	8.74134200	1.06349300
O	-5.62250000	6.39862400	1.58301500
H	-3.33775500	7.06719700	2.25497600
H	-5.55865900	7.93203700	2.97800100
H	-6.56050900	6.32779600	1.36329000
H	-7.20154900	8.61538700	1.23958200
H	-5.86559600	9.77492700	1.30638900
H	-5.92907100	8.58503600	-0.00099500
N	-3.54300100	6.73436700	-0.34104100
C	-3.61018800	6.69891600	-1.80228600
C	-2.82719700	5.51134600	-2.36040800
H	-4.21022800	6.13514600	0.14033700
H	-3.21285800	7.64737800	-2.16613600
H	-2.93825600	5.44151500	-3.44658400
H	-1.76233100	5.61861300	-2.12409000
H	-3.18486600	4.57205700	-1.92629800
H	-1.48498300	-0.72166000	2.35830900
H	2.44694700	-0.75874300	1.43656000
H	0.58874300	-2.74588200	1.90374600
H	0.47099400	-3.63337300	3.16113100
H	5.63469000	0.45516700	-2.98467400
H	6.46637100	1.62253500	-3.55678700
H	3.55505500	-3.68465400	-3.63190100
H	2.18754400	-4.13867200	-4.26368700
H	-3.41708400	8.82673000	2.23990800
H	-4.66558400	6.62889800	-2.08539600
H	-10.93236900	3.04725500	-1.03340700
H	-8.79942700	-2.63286200	0.55074700
H	-4.05444200	-9.79660600	0.31456100
H	-4.07805100	-3.42052800	-6.16887900
H	-1.23192600	-6.17910900	-3.49814300
H	2.39273400	-9.36602300	-0.27851300
H	8.18347000	-4.64348600	-5.76664500
H	8.46893000	-3.94594600	-4.13030800
H	7.21317000	-4.37524400	0.05911900
H	4.56173700	-3.31617200	4.40866600
H	6.17024500	-2.67305700	4.91348500
H	10.59652300	2.34081200	-1.37203700
H	10.09276700	1.02667600	-2.50408100
H	8.50348800	7.57800800	-0.32810100
H	4.80888800	4.08809700	6.30933400
H	3.09329900	4.01810200	5.87718200
H	1.77999400	-0.04245600	6.94952900
H	0.00030600	0.20162400	6.70234700
H	4.83142600	5.41415300	-3.07960000
H	0.69587700	3.54289300	-5.19617100
H	0.57188700	-1.65798400	-6.42279200
H	-4.41995100	-1.18154800	5.17861700
H	-2.96349100	-2.74993500	7.19007200
H	-2.72198900	-3.25544500	8.86989400
H	-2.61022900	-5.99300000	4.63495500

Int$_{Mn}$ (+9.8)

C	4.18894500	3.93021700	5.56633400
C	4.40746100	2.72412600	4.64633900
C	3.64129500	2.90882600	3.31927500
C	3.58618900	1.62572700	2.50272700
O	4.65378700	1.17712900	2.00388300
O	2.46863600	1.02163400	2.39036000
H	4.29288600	4.87075500	5.01452100
H	5.46721000	2.58107500	4.42125000
H	4.05821600	1.81248800	5.14772400
H	2.62192800	3.25542800	3.51676900
H	4.16774300	3.67878900	2.74284800
C	-10.18460900	3.72302000	-0.42727700
C	-8.74320300	3.41807500	-0.69363000
C	-7.75280100	3.38863300	0.26288700
C	-8.07255400	3.28523900	-1.96556700
C	-6.67621400	3.17742300	-1.69353000
C	-8.50244400	3.27154100	-3.30391800
N	-6.51185400	3.24603800	-0.32741900
C	-5.72177600	3.04654800	-2.70986300
C	-7.55737100	3.15282900	-4.31680200
C	-6.18140300	3.04031800	-4.02204700
H	-10.46892100	4.66464500	-0.91643700
H	-10.33925900	3.87180700	0.64699900
H	-7.84377900	3.48373800	1.33539000
H	-5.61663800	3.13444900	0.13738700
H	-9.55888600	3.35327600	-3.54253100
H	-4.66794900	2.95199500	-2.47486600
H	-7.88019300	3.14180500	-5.35324200
H	-5.46787500	2.94298200	-4.83489900
C	-7.95016200	-1.95180000	0.48286600
C	-7.58578800	-1.75248700	-0.96436600
C	-7.18318400	-2.84013400	-1.75159900
C	-7.61374200	-0.47846000	-1.54550800
C	-6.81752900	-2.65728300	-3.08339800
C	-7.23118600	-0.28909600	-2.87556800
C	-6.82800300	-1.37914800	-3.64650600
H	-8.31550100	-1.02163700	0.92804400
H	-7.07818100	-2.27060300	1.06764200
H	-7.15262800	-3.83503500	-1.31527200
H	-7.93795800	0.37585700	-0.95585300
H	-6.52736900	-3.51492800	-3.68182600
H	-7.25726600	0.70459100	-3.30525300
H	-6.52689600	-1.23560900	-4.67957900
C	7.92949600	7.57647200	0.77253200
C	7.70959900	6.13957100	1.18806300
C	8.42077700	5.59397700	2.26473500
C	6.76879200	5.31467700	0.55576900
C	8.19161200	4.29738700	2.71256200
C	6.52412300	4.00940300	0.98862500
C	7.22797700	3.49462100	2.09041400

O	7.02084400	2.25528400	2.59584200
H	8.50987100	8.11010100	1.53120200
H	6.97498000	8.10540400	0.66462300
H	9.16128900	6.20580800	2.77433500
H	6.21772500	5.69703900	-0.30158300
H	8.73962400	3.88708700	3.55421400
H	5.80692800	3.37848200	0.47126000
H	6.16470900	1.87911900	2.27536400
C	3.94480700	4.92884900	-2.65236400
C	2.99070400	4.71429200	-3.81248600
O	2.66466000	5.63465700	-4.56356900
C	3.18381500	5.57507700	-1.46104100
C	2.15932700	4.67048700	-0.84967100
C	2.17669300	3.93245200	0.31050100
N	0.96436800	4.32636500	-1.46299300
C	0.33370900	3.39605700	-0.69948500
N	1.04251300	3.14583600	0.39738800
H	4.40668800	3.99290300	-2.32654600
H	3.89889300	5.84074700	-0.67778600
H	2.71827000	6.50359000	-1.81023300
H	0.69219700	4.60812200	-2.39461200
H	2.93525300	3.92512000	1.07425800
H	-0.60283700	2.92703500	-0.96020400
N	2.41461500	3.48170000	-3.90256600
C	1.55502200	3.17157100	-5.05002000
C	1.36985800	1.65053900	-4.98846800
C	1.67098100	1.28190100	-3.52774100
C	2.77203800	2.26883500	-3.13839100
H	2.06350700	3.48394700	-5.96943900
H	0.37504700	1.34483000	-5.30453700
H	2.09813100	1.16297900	-5.64404600
H	0.78126500	1.42475700	-2.90486600
H	2.00501100	0.24874500	-3.41075000
H	2.79255700	2.47081200	-2.06582400
H	3.76249800	1.90248400	-3.42413700
C	-3.72942600	-3.03618400	-5.17218000
C	-2.55413700	-3.91346800	-4.82530000
O	-1.63783000	-4.15451700	-5.61509200
H	-3.41279900	-1.99199300	-5.13944000
H	-4.56604900	-3.14923100	-4.48754200
N	-2.55815700	-4.40302100	-3.55916600
C	-1.41126000	-5.15114500	-3.05995100
C	-1.81665900	-5.50464200	-1.62776000
C	-2.66946400	-4.30604400	-1.20836600
C	-3.46401600	-3.98776300	-2.47252900
H	-0.51340100	-4.52025200	-3.08021600
H	-2.42079200	-6.41564800	-1.62862100
H	-0.96453900	-5.67656300	-0.96959800
H	-3.32217200	-4.50865600	-0.36236100
H	-2.02246300	-3.46573000	-0.95370400
H	-4.40077500	-4.56073200	-2.50741500
H	-3.71288200	-2.92762400	-2.54255400
C	0.37917800	-1.54167400	-5.37194600
C	-0.83026000	-0.87203000	-4.77654600
C	-1.11192200	-1.07383600	-3.41604100
C	-1.70258900	-0.06531400	-5.51675900
C	-2.23101000	-0.49912700	-2.81775500
C	-2.82196800	0.52338700	-4.91752900
C	-3.09662400	0.30372300	-3.56693200
H	0.28039500	-2.62305500	-5.21882900
H	1.29720200	-1.22123900	-4.86901600
H	-0.45782600	-1.70051500	-2.81619100
H	-1.50955700	0.09270900	-6.57437800
H	-2.44089400	-0.70475400	-1.77580000
H	-3.49182800	1.13567200	-5.51503300
H	-3.98537900	0.72708200	-3.11041000
C	9.76222100	1.89606400	-1.76993700
C	9.22625400	1.03728000	-0.63579600
O	9.95759100	0.49296400	0.18457500
H	8.98535600	2.55742500	-2.16853100
N	7.86802600	0.91875300	-0.62398900

C	7.23973400	-0.02541100	0.25442900
C	7.21827800	-1.45236500	-0.30540700
O	7.49283700	-1.73028800	-1.47195000
H	7.31683900	1.24176400	-1.41787200
H	7.74715300	-0.00852900	1.22147600
H	6.20157100	0.27897000	0.41849600
N	6.80206400	-2.37971900	0.59763700
C	6.41413100	-3.71645700	0.18372500
C	5.29247500	-3.71950100	-0.87392000
C	4.20564400	-2.77550900	-0.46446900
C	3.49028000	-2.68852900	0.70459900
N	3.81524800	-1.69526100	-1.24006000
C	2.89571400	-0.99033600	-0.58227600
N	2.68808400	-1.57920200	0.59744000
H	6.43879100	-2.05036400	1.49127600
H	6.05954100	-4.22370200	1.08763600
H	5.68981800	-3.41994000	-1.84298000
H	4.89831700	-4.73664900	-0.98394300
H	4.22402700	-1.39008100	-2.20170100
H	3.48251700	-3.29181800	1.59385000
H	2.38703000	-0.10614100	-0.92358700
C	7.79578700	-3.70257100	-5.20029200
C	7.43653600	-2.30115800	-5.70082200
C	6.83507200	-1.37167200	-4.63763900
C	5.47104100	-1.74096400	-4.06436400
O	5.01079700	-2.90042200	-4.19512200
O	4.87072100	-0.81104100	-3.40348500
H	6.88150200	-4.18042900	-4.83786900
H	8.32900100	-1.80838300	-6.10741700
H	6.73001900	-2.39420700	-6.53539000
H	7.49160700	-1.29890600	-3.76027200
H	6.74664700	-0.35410000	-5.03826500
C	5.22430200	-2.58586500	4.80398000
C	4.92668200	-1.24290600	4.15191600
O	5.18132600	-1.35469200	2.76005400
H	4.90982300	-2.59514900	5.85212600
H	3.88012900	-0.95881200	4.31956600
H	5.54966500	-0.45652800	4.60382800
H	4.94813500	-0.49158900	2.34936800
C	1.11029400	-0.04561400	6.23591700
C	1.11560400	-0.98663500	5.03535500
C	-0.00775200	-0.69310100	4.04552800
O	-0.24777700	0.53121000	3.76488500
O	-0.63341600	-1.66170200	3.53347700
H	1.27796000	0.96962600	5.85578700
H	2.04245800	-0.84357500	4.47002300
H	1.07846200	-2.03656200	5.32151800
C	-3.85039600	-1.10956700	4.26396500
C	-4.34071300	0.22812200	3.77105000
C	-5.63586000	0.37151700	3.25820100
C	-3.51576500	1.35933200	3.81758200
C	-6.10537100	1.61209300	2.82994700
C	-3.98517000	2.60494700	3.39635900
C	-5.28483200	2.74056300	2.90828900
H	-2.76432600	-1.13407700	4.35955400
H	-4.13204400	-1.90451100	3.56574100
H	-6.28294300	-0.49943900	3.19026000
H	-2.49279800	1.24261200	4.16215600
H	-7.11158600	1.69014000	2.43123000
H	-3.33418100	3.47270200	3.42991000
H	-5.63730600	3.71872600	2.59194300
C	-2.40420400	-3.80987800	7.77508900
C	-0.93065700	-4.08978000	7.46188600
C	-0.53612300	-4.07433600	5.97772500
C	-1.01334600	-5.17198500	5.06961300
O	-2.24071500	-5.63301600	5.32410700
O	-0.35326300	-5.57768500	4.11575000
H	-3.03375700	-4.67796200	7.56905300
H	-0.31397900	-3.31839200	7.93715800
H	-0.62500700	-5.04047400	7.91453300
H	-0.88151300	-3.14895300	5.49364800

H	0.55229100	-4.07330600	5.88099200
C	-2.04089700	-0.51661900	1.06882000
C	-2.30061000	0.93464300	0.82338300
C	-3.55659900	1.34004400	0.23095300
C	-4.50344900	0.41495900	-0.09252000
C	-4.28940500	-0.99057500	0.14769900
C	-3.14029000	-1.43828800	0.69553300
O	-1.47602000	1.83582800	1.10797000
O	-0.56230100	-2.09583200	-0.00386900
O	0.34039600	-0.19192500	0.75098700
Mn	0.51117200	1.54124000	1.97806000
O	-0.22522200	3.27088300	3.30869300
O	1.11188900	-3.59813200	2.74904800
O	-2.06424700	4.55575400	1.22371800
O	6.01656500	1.56089500	-2.80347300
C	2.00655800	-8.33908200	-0.70999500
C	2.97144700	-7.15325900	-0.89856700
N	2.33563500	-5.92581400	-1.38834000
C	1.63711700	-5.04900700	-0.63577500
N	1.24972200	-3.90053900	-1.17793400
N	1.29623000	-5.34535000	0.63144400
H	1.36550100	-8.42641200	-1.59248000
H	1.34591300	-8.19331400	0.15036800
H	3.51570300	-6.94266100	0.03234400
H	3.72941600	-7.41201100	-1.64115500
H	2.59045700	-5.56596300	-2.31135900
H	1.66832800	-3.65019200	-2.07989900
H	0.67554700	-3.22440000	-0.65947800
H	1.68479300	-6.18052900	1.03755800
H	1.08651100	-4.57951500	1.29853900
C	-3.47647300	-9.01268400	-0.51513600
C	-3.06335100	-7.90271900	0.41158700
C	-1.85487500	-7.76062300	1.04991200
C	-3.88468500	-6.80611900	0.86075300
C	-3.09683900	-6.02588800	1.75923800
C	-5.19052900	-6.38292400	0.56181400
N	-1.86794400	-6.64109200	1.86421100
C	-3.57311300	-4.83931400	2.33234800
C	-5.67264500	-5.21442100	1.14099000
C	-4.87037800	-4.44955800	2.01261800
H	-2.62287400	-9.42442800	-1.06052700
H	-4.22072300	-8.67968100	-1.24350600
H	-0.97193300	-8.38106200	0.98757400
H	-1.10725200	-6.30452500	2.44136000
H	-5.81258900	-6.95711400	-0.11860600
H	-2.94778100	-4.22385600	2.97170300
H	-6.68233600	-4.88141900	0.92271000
H	-5.26970500	-3.53388500	2.43728100
O	2.72513600	-3.98845900	-3.52410600
H	-5.07507300	-1.67971500	-0.13707800
H	-5.43881000	0.73576100	-0.53620600
H	-2.97676300	-2.49117500	0.87615200
C	-0.63172600	-0.98079500	0.53411800
O	-3.73549800	2.67521900	0.01556100
H	-3.07855500	3.19699000	0.54139000
H	-2.37190000	5.39189200	0.81430800
H	-1.24248800	4.31832600	0.76797400
H	-0.59704700	2.68797500	3.98919500
H	-0.97452100	3.70292600	2.86151200
C	-3.81271300	7.85327600	2.09136200
C	-3.36105100	7.90933600	0.64518300
O	-2.83285500	8.88446100	0.13799700
C	-5.33609200	7.71206000	2.25073200
C	-6.12431800	8.73910900	1.44532100
O	-5.66541500	6.36435700	1.84812000
H	-3.35570700	6.98238700	2.57823700
H	-5.57245700	7.82421900	3.31764800
H	-6.60780600	6.31948500	1.64033800
H	-7.20126700	8.62019300	1.60909300
H	-5.84984500	9.75431300	1.74491300
H	-5.92320100	8.63718600	0.37463900
N	-3.60551200	6.72447200	-0.03512200
C	-3.66462600	6.74359000	-1.50078000
C	-2.86530400	5.59349200	-2.11051700
H	-4.34455100	6.17975500	0.41016100
H	-3.28250400	7.71111600	-1.82955400
H	-2.97917700	5.57482200	-3.19833800
H	-1.80077100	5.71040500	-1.87714600
H	-3.19963300	4.62909100	-1.71667600
H	-1.84430800	-0.63351800	2.15410400
H	2.01122000	-1.20755300	1.25602600
H	0.43094900	-2.90299000	2.91055100
H	0.85708200	-4.29804300	3.37459600
H	5.65992700	0.65416100	-2.99763400
H	6.52904800	1.78408400	-3.59002400
H	3.59175800	-3.51077200	-3.72593400
H	2.24983100	-4.02131800	-4.36367700
H	-3.43198100	8.74247300	2.60466900
H	-4.72343800	6.67179200	-1.77047300
H	-10.93769300	2.99316400	-0.74273400
H	-8.72488400	-2.71239900	0.62583700
H	-3.91294900	-9.81492100	0.08893400
H	-4.09730200	-3.23460600	-6.18426800
H	-1.18401700	-6.04764800	-3.64598700
H	2.52010400	-9.29838400	-0.58416100
H	8.18028900	-4.34202800	-6.00178300
H	8.48329100	-3.69444700	-4.34803300
H	7.29494300	-4.27115000	-0.15661500
H	4.69878900	-3.37995800	4.26316900
H	6.30821900	-2.73666800	4.76472500
H	10.58957600	2.52232300	-1.41998900
H	10.08193600	1.24006100	-2.58633000
H	8.46047600	7.70122100	-0.17699100
H	4.90075400	3.96162300	6.39786600
H	3.17968000	3.88765100	5.98896200
H	1.92293100	-0.21867900	6.94900200
H	0.13746900	0.01477300	6.73578900
H	4.76922700	5.58867200	-2.94267900
H	0.62118900	3.74334700	-5.05758900
H	0.53055700	-1.41636700	-6.44935500
H	-4.29122600	-1.36502200	5.23322500
H	-2.78873700	-2.98148700	7.17148400
H	-2.51723000	-3.53830700	8.83010600
H	-2.48833600	-6.25508700	4.61585700

$TS2_{Mn}$ (+11.8)

C	4.18156500	3.96811300	5.50644500
C	4.40359800	2.77549600	4.56621200
C	3.61844300	2.96612600	3.25153800
C	3.61232700	1.73616900	2.35097900
O	4.71538000	1.23030400	2.00937800
O	2.49637100	1.25011100	1.97137400
H	4.26895300	4.91361900	4.95936900
H	5.46340100	2.65162700	4.33151400
H	4.07591200	1.85103800	5.05928900
H	2.58743500	3.26666400	3.46127700
H	4.10193100	3.78164900	2.69723400
C	-10.24452000	3.60338900	-0.35148500
C	-8.81138900	3.31117400	-0.65578000
C	-7.79677300	3.30961000	0.27433700
C	-8.17508800	3.16543200	-1.94317500
C	-6.77092300	3.08228800	-1.70636600
C	-8.63978900	3.12115500	-3.26918900
N	-6.57252400	3.17433700	-0.34703500
C	-5.84087000	2.95284100	-2.74515900
C	-7.71969600	2.99888800	-4.30436100
C	-6.33448500	2.91595600	-4.04449600
H	-10.55152300	4.54394500	-0.82900900
H	-10.37493600	3.74478600	0.72694100
H	-7.85967800	3.42498900	1.34702600

H	-5.66456000	3.07010000	0.09361300
H	-9.70322300	3.18302200	-3.48129700
H	-4.77879200	2.88636200	-2.53862100
H	-8.06916000	2.96435400	-5.33160800
H	-5.64076600	2.81893300	-4.87437600
C	-7.95909500	-2.04651400	0.58480100
C	-7.60039400	-1.85811900	-0.86555500
C	-7.20810500	-2.95227500	-1.64882900
C	-7.62731100	-0.58777100	-1.45480800
C	-6.85200700	-2.77995500	-2.98480700
C	-7.25660300	-0.40921800	-2.78967300
C	-6.86351900	-1.50577000	-3.55667100
H	-8.33023100	-1.11477600	1.02174700
H	-7.07990800	-2.35008700	1.16741300
H	-7.18190700	-3.94522000	-1.20769100
H	-7.94320000	0.27206500	-0.86878300
H	-6.57065000	-3.64289700	-3.58008700
H	-7.28467100	0.58122700	-3.22650000
H	-6.57200000	-1.36993900	-4.59351000
C	7.85018000	7.60210800	0.64813100
C	7.67229400	6.16816800	1.09182200
C	8.43435100	5.64955900	2.14684800
C	6.72230700	5.31931400	0.50727400
C	8.24412300	4.35595600	2.62116600
C	6.51729100	4.01655300	0.96633300
C	7.26996700	3.52980700	2.04821400
O	7.09773100	2.29556000	2.58045800
H	8.42095800	8.16596100	1.39221400
H	6.88087300	8.10238200	0.53690000
H	9.18320600	6.28088800	2.61917100
H	6.13074700	5.68076100	-0.33199500
H	8.82955600	3.96715400	3.44770000
H	5.79195600	3.36716600	0.48488800
H	6.23543400	1.91014400	2.29244200
C	3.85419300	4.89626700	-2.71763400
C	2.90930900	4.65452700	-3.87860000
O	2.60776200	5.55663500	-4.66296200
C	3.09634800	5.64039500	-1.58274900
C	2.03288400	4.81745600	-0.93606700
C	2.02945300	4.12352500	0.24715000
N	0.82461000	4.49327300	-1.53718100
C	0.16189200	3.61897800	-0.73771700
N	0.86775600	3.38366500	0.35962900
H	4.30311600	3.97557100	-2.33740900
H	3.80906100	5.93119000	-0.80601100
H	2.67492400	6.56050600	-2.00269000
H	0.54757200	4.77398800	-2.46739100
H	2.79008400	4.10830200	1.00926700
H	-0.79748700	3.18392700	-0.97153700
N	2.31802400	3.43071700	-3.93867400
C	1.45575600	3.10122500	-5.07837100
C	1.26567500	1.58398800	-4.97242500
C	1.49827600	1.27279300	-3.48600900
C	2.60802200	2.25169900	-3.09719100
H	1.95828400	3.39546100	-6.00599500
H	0.28635200	1.26733000	-5.32432800
H	2.02504600	1.07429800	-5.57436700
H	0.59127700	1.46281900	-2.90274300
H	1.79793400	0.23756100	-3.30742700
H	2.58276500	2.51121900	-2.03684600
H	3.60128900	1.84873500	-3.31833900
C	-3.78287200	-3.14672400	-5.10013500
C	-2.60459600	-4.02001800	-4.75414100
O	-1.69925000	-4.27673900	-5.55080000
H	-3.46764200	-2.10197400	-5.08111800
H	-4.61313700	-3.25410300	-4.40639100
N	-2.59080500	-4.49325300	-3.47963600
C	-1.42919300	-5.22657400	-2.99202300
C	-1.79142400	-5.53761600	-1.53936000
C	-2.62113100	-4.31765500	-1.12869400
C	-3.45793300	-4.03780200	-2.37692100
H	-0.53638100	-4.58836100	-3.04800800
H	-2.40585600	-6.44003200	-1.49221500
H	-0.91879400	-5.70091300	-0.90537600
H	-3.24418000	-4.48572000	-0.25256000
H	-1.96356600	-3.46926200	-0.92833500
H	-4.39626600	-4.60827100	-2.35793200
H	-3.70700100	-2.98010700	-2.47464300
C	0.31264800	-1.62315100	-5.35031900
C	-0.91109700	-0.95160400	-4.78639600
C	-1.20850500	-1.11078700	-3.42474400
C	-1.77798300	-0.17526300	-5.56449300
C	-2.33488500	-0.51792000	-2.85788600
C	-2.90667100	0.42803500	-4.99899600
C	-3.19433300	0.25485200	-3.64448400
H	0.22633000	-2.70294400	-5.17948900
H	1.22355800	-1.27634200	-4.85208300
H	-0.55473500	-1.71204000	-2.79984500
H	-1.57133400	-0.04979600	-6.62389800
H	-2.55542900	-0.67505700	-1.80942300
H	-3.57115800	1.01785500	-5.62439400
H	-4.08737900	0.69334700	-3.21150400
C	9.70224400	1.91451800	-1.86414800
C	9.19451100	1.05502400	-0.71824100
O	9.94643300	0.51202700	0.08443100
H	8.91315600	2.56432400	-2.25705900
N	7.83799400	0.93144300	-0.67505100
C	7.23501000	-0.01385700	0.21904000
C	7.21265300	-1.44293800	-0.33547300
O	7.47019100	-1.72476400	-1.50461300
H	7.26515100	1.25812500	-1.45184900
H	7.76150700	0.00966100	1.17589900
H	6.19845200	0.28499000	0.40210100
N	6.81221100	-2.36964100	0.57567900
C	6.41472900	-3.70638900	0.16684100
C	5.25769200	-3.70181200	-0.85501500
C	4.20020000	-2.73793100	-0.40485900
C	3.50811800	-2.65992200	0.77713800
N	3.85176100	-1.60823700	-1.13037700
C	2.98929700	-0.87579500	-0.42498500
N	2.75925900	-1.50390800	0.73302400
H	6.46453900	-2.03827300	1.47423000
H	6.07842300	-4.21164000	1.07926800
H	5.62182200	-3.41225400	-1.83992500
H	4.84634600	-4.71406200	-0.94458300
H	4.23160600	-1.32904700	-2.11051100
H	3.48703900	-3.29773300	1.64293300
H	2.54041700	0.05655700	-0.71782500
C	7.74656800	-3.72704000	-5.22967700
C	7.36311500	-2.32959300	-5.72528600
C	6.78373200	-1.39887800	-4.64888000
C	5.43212300	-1.76329800	-4.04266200
O	4.96934800	-2.92418100	-4.16341300
O	4.84691900	-0.83157700	-3.37314100
H	6.84211800	-4.21291500	-4.85214100
H	8.24123900	-1.83119400	-6.15556000
H	6.63644500	-2.42985100	-6.54151600
H	7.45952700	-1.32499200	-3.78664400
H	6.68879500	-0.38113600	-5.04729400
C	5.25911700	-2.54624600	4.78838700
C	4.95283100	-1.20278000	4.13885700
O	5.26268400	-1.28936500	2.75825700
H	4.95464800	-2.55333300	5.83985100
H	3.89353700	-0.93748100	4.26551900
H	5.53921400	-0.40845300	4.62438900
H	5.03585400	-0.42233800	2.35339100
C	1.13935300	-0.02481500	6.23737800
C	1.14961200	-1.02632700	5.08827600
C	0.61788300	-0.50817700	3.75407000
O	0.12123700	0.65762400	3.69127100
O	0.70933000	-1.28149300	2.75335300
H	1.30914400	0.97492600	5.82281200

H	2.16218000	-1.38425400	4.88090700
H	0.58440900	-1.92570800	5.33045700
C	-3.83148400	-1.14292000	4.32032200
C	-4.30357900	0.20730000	3.84381200
C	-5.57822500	0.35810100	3.28505700
C	-3.48905200	1.33923900	3.96341500
C	-6.03680500	1.60880000	2.87581200
C	-3.94760500	2.59500100	3.56191100
C	-5.22610000	2.73789900	3.01983400
H	-2.74140500	-1.16159500	4.42087600
H	-4.11822200	-1.93175800	3.61922500
H	-6.21457800	-0.51429600	3.16047100
H	-2.47710200	1.22386500	4.34351700
H	-7.02503400	1.69239700	2.43581800
H	-3.30058500	3.46148800	3.63738000
H	-5.56833800	3.72245800	2.71111100
C	-2.33170200	-3.80175000	7.84012800
C	-0.86588200	-4.08742300	7.50844900
C	-0.52859600	-4.07904200	6.01275400
C	-0.98317700	-5.20712000	5.12903500
O	-2.14607800	-5.76991900	5.45839900
O	-0.35533600	-5.54105100	4.12870300
H	-2.96292900	-4.67292700	7.65061900
H	-0.23632900	-3.31293600	7.96064200
H	-0.55043800	-5.03595800	7.95744000
H	-0.96925100	-3.18103800	5.55624600
H	0.55018200	-4.00753300	5.85706400
C	-2.07677600	-0.36986400	1.18940000
C	-2.36421200	1.02250100	0.96573700
C	-3.57804900	1.36280800	0.28703500
C	-4.47242600	0.39279700	-0.08900200
C	-4.21298100	-0.98209100	0.17789700
C	-3.06899600	-1.34820200	0.83276800
O	-1.55373600	1.96017100	1.31112600
O	-0.42765300	-1.87411500	-0.22188700
O	0.23306500	0.29768900	0.07067900
Mn	0.50190000	1.78689500	1.86953600
O	0.00746300	3.47209500	3.34897200
O	1.52741500	-3.81755200	2.88686300
O	-2.13243500	4.50665700	1.69185300
O	5.90238800	1.57487200	-2.77795400
C	2.03435600	-8.36914800	-0.64777400
C	3.01093700	-7.22391500	-0.96816600
N	2.36585900	-5.98980000	-1.42210200
C	1.73079500	-5.11250700	-0.61986000
N	1.31605000	-3.96348300	-1.14303600
N	1.48091600	-5.41277800	0.66759900
H	1.32462700	-8.48710600	-1.47188200
H	1.44933700	-8.17636200	0.25632200
H	3.65660900	-7.01082200	-0.10464100
H	3.67677500	-7.52824100	-1.77826000
H	2.57918500	-5.62228800	-2.35300900
H	1.69762900	-3.70508300	-2.06277000
H	0.79504500	-3.28175000	-0.60025800
H	1.90595200	-6.25139600	1.02890000
H	1.37678900	-4.66083200	1.37779900
C	-3.44146700	-9.08197700	-0.39634000
C	-3.02578300	-7.96065200	0.51085400
C	-1.81359100	-7.81340300	1.13899800
C	-3.84487400	-6.85973100	0.95339900
C	-3.05419200	-6.07629300	1.84571700
C	-5.15059900	-6.43642600	0.65411900
N	-1.82449200	-6.69062100	1.94787700
C	-3.53373800	-4.89247800	2.42130300
C	-5.63174700	-5.26577100	1.22937500
C	-4.83028800	-4.50103700	2.10163200
H	-2.59146000	-9.49440900	-0.94699900
H	-4.19545500	-8.76295200	-1.12105700
H	-0.92979900	-8.43234700	1.07408600
H	-1.05502600	-6.34031900	2.50302800
H	-5.77397300	-7.01294700	-0.02303500

H	-2.91173400	-4.28345200	3.06971600
H	-6.64048800	-4.93172900	1.00957100
H	-5.23181800	-3.58715400	2.52798800
O	2.69158300	-4.00152600	-3.51531500
H	-4.93960300	-1.72043200	-0.14098100
H	-5.38978900	0.67558900	-0.59206600
H	-2.86740700	-2.39045100	1.04751800
C	-0.44111100	-0.71534000	0.11378700
O	-3.83532500	2.69582000	0.04561400
H	-3.41266300	3.23896700	0.73840900
H	-2.02921500	5.18532600	1.00659500
H	-1.78482300	3.65319900	1.34049600
H	-0.39053600	2.94676500	4.05798600
H	-0.69918400	4.03497900	2.97302000
C	-3.88113700	7.80208100	2.07355600
C	-3.48508700	7.87445200	0.60920400
O	-3.07341600	8.89502600	0.07636800
C	-5.39541800	7.62514700	2.27500800
C	-6.23106100	8.63334600	1.49394600
O	-5.70618800	6.27094300	1.88094100
H	-3.39399400	6.93621200	2.53912100
H	-5.60591200	7.73202200	3.34838900
H	-6.64033400	6.22360000	1.64045500
H	-7.29947600	8.49197400	1.69360800
H	-5.96787000	9.65500400	1.78124300
H	-6.06119000	8.53266200	0.41794100
N	-3.64916900	6.67544500	-0.05369100
C	-3.75777400	6.66353200	-1.51051400
C	-2.96358200	5.50969900	-2.12045000
H	-4.26828800	6.03533900	0.43668900
H	-3.39193300	7.62828300	-1.86541700
H	-3.10440400	5.46776200	-3.20480900
H	-1.89579100	5.64102600	-1.91296100
H	-3.27889200	4.54933800	-1.69996000
H	-1.44410300	-0.58020700	2.05039100
H	2.14652300	-1.15282400	1.47424200
H	1.17209800	-2.89104900	2.88561500
H	0.93253900	-4.34964500	3.44156300
H	5.58899500	0.65682400	-2.99308400
H	6.34261900	1.87017100	-3.58414900
H	3.56276500	-3.51985800	-3.70733600
H	2.20522300	-3.99486000	-4.34917900
H	-3.50240700	8.69839300	2.57590100
H	-4.81847800	6.58176100	-1.76979200
H	-10.99503800	2.86542900	-0.65413500
H	-8.72659000	-2.81197900	0.74103700
H	-3.86654600	-9.88248300	0.21835000
H	-4.15799400	-3.35619300	-6.10713200
H	-1.20041900	-6.12615200	-3.57278400
H	2.55602000	-9.32347300	-0.51852200
H	8.12854600	-4.37031300	-6.02936900
H	8.44187700	-3.70672000	-4.38392400
H	7.29665100	-4.25727800	-0.17701100
H	4.73461000	-3.34865600	4.25914700
H	6.34371000	-2.68918300	4.74044200
H	10.52803800	2.54986500	-1.52708900
H	10.01936900	1.25415200	-2.67801500
H	8.37143400	7.72287700	-0.30727500
H	4.90084200	4.01175900	6.33107000
H	3.17666200	3.92153300	5.93875200
H	1.95982300	-0.18602600	6.94439700
H	0.17081700	0.03239800	6.74590400
H	4.67092000	5.55980600	-3.02101700
H	0.51761400	3.66593800	-5.08200000
H	0.45312100	-1.50576000	-6.43016200
H	-4.26072400	-1.39252600	5.29609700
H	-2.72886900	-2.98217800	7.23334000
H	-2.43778400	-3.52312800	8.89417400
H	-2.38832100	-6.40490400	4.75931800

E:P$_{Mn}$ (–4.9)

C	-4.15563100	-3.93378500	5.49730200
C	-4.36437600	-2.73933100	4.55346600
C	-3.54913500	-2.92595200	3.25686800
C	-3.54588900	-1.72359100	2.31521800
O	-4.64845700	-1.19395100	2.00853600
O	-2.43567800	-1.30072200	1.84924600
H	-4.24992700	-4.87836500	4.94963300
H	-5.41975600	-2.62132100	4.29731100
H	-4.05270800	-1.81340900	5.05385500
H	-2.51766100	-3.20912100	3.49012700
H	-4.00057300	-3.76263100	2.70642300
C	10.24601500	-3.63926200	-0.42461000
C	8.81180800	-3.29594500	-0.66766500
C	7.84651300	-3.18492200	0.30776900
C	8.12001900	-3.18294500	-1.92898300
C	6.73591300	-3.01021000	-1.63036900
C	8.51970400	-3.23092500	-3.27609000
N	6.60226000	-3.01344000	-0.26041200
C	5.76263800	-2.88892600	-2.62975400
C	7.55646800	-3.11330900	-4.27181500
C	6.19155400	-2.94629600	-3.95093300
H	10.51145600	-4.56915700	-0.94510100
H	10.41133800	-3.81981200	0.64320300
H	7.95925300	-3.24030100	1.38084700
H	5.71517400	-2.85297900	0.20886100
H	9.56698500	-3.35910500	-3.53459700
H	4.71913100	-2.75347200	-2.37173800
H	7.85561600	-3.14980100	-5.31495200
H	5.46227600	-2.85773700	-4.75101800
C	7.99888400	2.02184600	0.53791600
C	7.63691800	1.84461800	-0.91404800
C	7.26467200	2.94702600	-1.69540500
C	7.64528200	0.57630900	-1.50752900
C	6.90758300	2.78433700	-3.03245200
C	7.27456800	0.40685900	-2.84341000
C	6.90081800	1.51163300	-3.60812900
H	8.36557900	1.08485900	0.96689800
H	7.12287500	2.32357700	1.12544000
H	7.25686700	3.93972500	-1.25275700
H	7.94611100	-0.28942500	-0.92285000
H	6.64166600	3.65352400	-3.62653700
H	7.28582700	-0.58376800	-3.28157000
H	6.61039600	1.38372300	-4.64646400
C	-7.86788600	-7.53165700	0.64532400
C	-7.67234000	-6.09838600	1.08464100
C	-8.46572400	-5.54863700	2.10037000
C	-6.67731900	-5.28022100	0.53284100
C	-8.26234500	-4.25527400	2.56966100
C	-6.45823500	-3.97806100	0.98738800
C	-7.24236200	-3.46141100	2.03184400
O	-7.05803000	-2.22771200	2.56278900
H	-8.44285800	-8.08761700	1.39211600
H	-6.90472600	-8.04246200	0.53137900
H	-9.25012700	-6.15535600	2.54647900
H	-6.05969900	-5.66657700	-0.27575100
H	-8.87187100	-3.84264100	3.36665100
H	-5.69632900	-3.35260500	0.53120400
H	-6.18449600	-1.85934100	2.28986600
C	-3.87080000	-4.84039700	-2.73077800
C	-2.94275100	-4.59381700	-3.90319800
O	-2.66905900	-5.48825700	-4.70577400
C	-3.10877600	-5.63116400	-1.63183700
C	-2.04653600	-4.82610600	-0.97032900
C	-2.05445100	-4.14182000	0.21723200
N	-0.84660500	-4.47675700	-1.57239900
C	-0.19768400	-3.59715200	-0.76814100
N	-0.90692000	-3.38428800	0.33232200
H	-4.30378500	-3.92519500	-2.32111900
H	-3.81623900	-5.94959800	-0.86131700
H	-2.69377100	-6.53529800	-2.09062500
H	-0.56581700	-4.74597600	-2.50466700
H	-2.81538200	-4.14448100	0.97915100
H	0.75256500	-3.13713600	-0.99013400
N	-2.33157400	-3.37951400	-3.95499200
C	-1.47218800	-3.05300500	-5.09717200
C	-1.27011600	-1.53827200	-4.98580000
C	-1.47693500	-1.23899000	-3.49340300
C	-2.59455600	-2.20670900	-3.09814700
H	-1.98112200	-3.34005800	-6.02333200
H	-0.29403400	-1.22510000	-5.34957200
H	-2.03609800	-1.01935600	-5.57135700
H	-0.56165400	-1.45205800	-2.93073500
H	-1.75598300	-0.20052200	-3.29994400
H	-2.55932400	-2.47947300	-2.04086900
H	-3.58510000	-1.78801600	-3.30206300
C	3.80358800	3.16322200	-5.12485200
C	2.63595300	4.04643800	-4.76860500
O	1.73387600	4.32558100	-5.56151100
H	3.47736700	2.12166400	-5.10763300
H	4.63709100	3.25857100	-4.43217500
N	2.62579400	4.50719300	-3.48785700
C	1.47226000	5.25113200	-3.00033300
C	1.82586000	5.53994100	-1.54298300
C	2.60776800	4.28791900	-1.14108100
C	3.46182800	4.00675500	-2.37833300
H	0.57125100	4.62175100	-3.06340200
H	2.47172100	6.41873400	-1.47804600
H	0.95073100	5.72741500	-0.91858700
H	3.21364200	4.41081400	-0.24608900
H	1.91830300	3.45303100	-0.97618100
H	4.41233800	4.55335000	-2.32780300
H	3.68640900	2.94521100	-2.48951100
C	-0.30175300	1.66521500	-5.36092100
C	0.90690500	0.98579200	-4.77156500
C	1.17103600	1.14164100	-3.40169300
C	1.79193100	0.20900400	-5.52746800
C	2.28109400	0.54922900	-2.80123800
C	2.90796400	-0.39052900	-4.93234700
C	3.16151200	-0.21774200	-3.57104400
H	-0.19883300	2.74625400	-5.20007900
H	-1.22177200	1.33691600	-4.86555000
H	0.49674700	1.74781100	-2.80045400
H	1.61227300	0.08340500	-6.59179100
H	2.46718700	0.68761400	-1.74236700
H	3.59056400	-0.97761200	-5.54054400
H	4.04382800	-0.65203300	-3.11370100
C	-9.69682800	-1.82583000	-1.84243600
C	-9.18290000	-0.96791300	-0.69758000
O	-9.93128800	-0.41125800	0.09897400
H	-8.91198300	-2.47841700	-2.23891200
N	-7.82522200	-0.85849400	-0.64897800
C	-7.21616400	0.08183400	0.24631500
C	-7.18123200	1.51112200	-0.30735100
O	-7.42239300	1.79332100	-1.47996000
H	-7.25195500	-1.20206100	-1.41792000
H	-7.74193000	0.06159400	1.20354000
H	-6.18166100	-0.22520400	0.42862900
N	-6.78438900	2.43864200	0.60582400
C	-6.36639000	3.76934900	0.18961700
C	-5.20812100	3.73348700	-0.83385600
C	-4.18872500	2.72073800	-0.39524900
C	-3.49599700	2.60847200	0.78469200
N	-3.89819700	1.57503900	-1.12186400
C	-3.07523800	0.79934200	-0.41520000
N	-2.80576500	1.41573700	0.74118300
H	-6.44332400	2.10455500	1.50583100
H	-6.01954700	4.27253900	1.09921000
H	-5.58069300	3.47005600	-1.82262200
H	-4.75660600	4.72952300	-0.90969500
H	-4.27960900	1.31243800	-2.11795100
H	-3.45104100	3.24035400	1.65399000

H	-2.70980400	-0.17393100	-0.69604800
C	-7.72233100	3.81339100	-5.20078600
C	-7.34772700	2.41730500	-5.70524700
C	-6.77798700	1.47433300	-4.63528400
C	-5.42360900	1.81092100	-4.02215900
O	-4.93830000	2.96541900	-4.10804300
O	-4.85486400	0.85246400	-3.37267700
H	-6.81375000	4.29383300	-4.82489000
H	-8.22872800	1.92785100	-6.13958800
H	-6.62004400	2.51790200	-6.52049000
H	-7.45435300	1.40418100	-3.77293500
H	-6.69690800	0.45749800	-5.03835600
C	-5.19676800	2.58912700	4.80250000
C	-4.89240000	1.24385600	4.15771000
O	-5.24475500	1.31194000	2.78552000
H	-4.88769100	2.59776700	5.85270900
H	-3.82546700	0.99666100	4.25631800
H	-5.45403200	0.44696100	4.66718400
H	-5.01730200	0.44502400	2.38269200
C	-1.08626000	0.03856800	6.22594500
C	-1.07981500	1.03996700	5.07451700
C	-0.67814700	0.45653400	3.72188100
O	-0.20672000	-0.71725200	3.68497200
O	-0.84367700	1.18852300	2.69808200
H	-1.26159900	-0.95960600	5.81165000
H	-2.05789000	1.50797300	4.93173400
H	-0.39829900	1.86779100	5.26933100
C	3.88246500	1.13156300	4.28977500
C	4.23921600	-0.27664800	3.88768100
C	5.54734200	-0.59150400	3.50297200
C	3.28378600	-1.29817900	3.90899600
C	5.89987100	-1.89767000	3.16813100
C	3.63696900	-2.60626000	3.57677300
C	4.94571900	-2.91713500	3.20324400
H	2.79703700	1.25556600	4.35526900
H	4.25517800	1.85487600	3.55913700
H	6.29575800	0.19594100	3.46085300
H	2.25372800	-1.05980500	4.16161300
H	6.92373900	-2.11243400	2.87998000
H	2.88890900	-3.38871500	3.57989500
H	5.20774400	-3.93805800	2.93756700
C	2.41504300	3.79022200	7.82371200
C	0.94954500	4.08761200	7.50208100
C	0.60417500	4.06103100	6.00972400
C	1.05413700	5.17354400	5.10523400
O	2.19082400	5.77884200	5.44435300
O	0.44132700	5.45800100	4.07926000
H	3.05141800	4.65765600	7.63361800
H	0.31586200	3.32519300	7.96866500
H	0.64740200	5.04499900	7.94117300
H	1.04647700	3.15845300	5.56438400
H	-0.47489700	3.98280200	5.85823300
C	1.99765000	0.47799500	1.05891300
C	2.26934100	-0.87406500	0.79614300
C	3.55290400	-1.20089800	0.30431000
C	4.49696900	-0.21805600	0.03152800
C	4.20338900	1.12577800	0.28435600
C	2.95856700	1.46195900	0.81821900
O	1.37174800	-1.84679700	0.99528700
Mn	-0.43452500	-1.91076800	1.94281300
O	0.10340100	-3.64443700	3.25479100
O	-1.47830000	3.77178100	2.89498300
O	2.07034000	-4.32302800	1.49312300
O	-5.89836700	-1.55266000	-2.74514600
C	-1.96156500	8.40791700	-0.63164900
C	-2.93501900	7.27638200	-1.00232400
N	-2.28238700	6.01653900	-1.37859000
C	-1.73012900	5.15822500	-0.50400400
N	-1.26518500	3.98831100	-0.96729400
N	-1.59316100	5.47139800	0.78990100
H	-1.22354700	8.53503500	-1.42900900
H	-1.40695000	8.19246000	0.28657400
H	-3.64752900	7.09200100	-0.18614400
H	-3.53183800	7.57319300	-1.86686000
H	-2.46144300	5.61143800	-2.30161900
H	-1.65005800	3.67403500	-1.87110500
H	-0.98078200	3.29347500	-0.29322000
H	-2.01979200	6.32318800	1.11541300
H	-1.45999300	4.72863100	1.51247700
C	3.51970800	9.08703300	-0.40280900
C	3.09691400	7.96636100	0.50160400
C	1.89092800	7.83241900	1.14425600
C	3.90692200	6.85156500	0.92702100
C	3.11922100	6.07503600	1.82664200
C	5.20186900	6.41201800	0.60494900
N	1.89821700	6.70602100	1.94974500
C	3.59168500	4.88288300	2.39072700
C	5.67528100	5.23219800	1.16735200
C	4.87722000	4.47524600	2.04897900
H	2.67252600	9.50802800	-0.95163400
H	4.27005200	8.76389400	-1.12938100
H	1.01550600	8.46489800	1.09563800
H	1.12852500	6.35226700	2.50255600
H	5.82227600	6.98290000	-0.07966700
H	2.97159600	4.27531300	3.04190800
H	6.67400000	4.88266300	0.92792300
H	5.26892700	3.55058600	2.45972500
O	-2.60757500	3.90666200	-3.36377800
H	4.93872500	1.89044500	0.05967400
H	5.46089900	-0.50571600	-0.37099000
H	2.73098100	2.49651500	1.04786900
O	3.87608400	-2.53048100	0.06512800
H	3.45279300	-3.09231000	0.73903500
H	1.97560200	-4.98516300	0.79231200
H	1.76372600	-3.43277600	1.14602300
H	0.51913400	-3.29637500	4.05495700
H	0.80412400	-4.12306500	2.74725400
C	3.86829600	-7.80644500	2.01720400
C	3.48265200	-7.88023000	0.55050800
O	3.08950700	-8.90693000	0.01358600
C	5.37750700	-7.61236500	2.23351300
C	6.23602400	-8.59055400	1.43910300
O	5.66193500	-6.24642600	1.86642400
H	3.37012200	-6.94350700	2.47708000
H	5.58257100	-7.73671800	3.30629600
H	6.60188600	-6.16344500	1.66038900
H	7.30020800	-8.43638400	1.65131300
H	5.98624500	-9.62216100	1.70201700
H	6.07429600	-8.46854300	0.36418200
N	3.63286500	-6.67989600	-0.10745800
C	3.73571200	-6.65704400	-1.56304700
C	2.95344200	-5.48403600	-2.15280000
H	4.22631500	-6.02000900	0.38804700
H	3.35857200	-7.61423600	-1.92587500
H	3.10129700	-5.41722700	-3.23491800
H	1.88300200	-5.61036600	-1.95425900
H	3.27623100	-4.53599700	-1.70977200
H	1.02656600	0.74407700	1.46554100
H	-2.21347000	1.03133300	1.48894100
H	-1.18720700	2.82147500	2.86183500
H	-0.83297000	4.25626900	3.43900900
H	-5.60204600	-0.63801200	-2.99071200
H	-6.32734600	-1.88887900	-3.54125600
H	-3.50022200	3.48333800	-3.59040000
H	-2.08473800	3.84378400	-4.17360400
H	3.48641800	-8.70183500	2.51875700
H	4.79568700	-6.58060600	-1.82678700
H	10.99953000	-2.90490500	-0.72844800
H	8.77181300	2.78189900	0.69307300
H	3.95199400	9.88318800	0.21228900
H	4.17602700	3.37342300	-6.13287400
H	1.24650300	6.15389000	-3.57738500

H	-2.47692600	9.36497900	-0.49708900
H	-8.10391200	4.46131900	-5.99687300
H	-8.41385300	3.79489800	-4.35189400
H	-7.24639100	4.32648100	-0.14868000
H	-4.67017100	3.38972600	4.27320700
H	-6.28097500	2.73847300	4.76017300
H	-10.52492200	-2.45717000	-1.50346100
H	-10.01362500	-1.16125400	-2.65300100
H	-8.39414500	-7.64655700	-0.30807800
H	-4.87139200	-3.97543500	6.32507500
H	-3.14848400	-3.89448000	5.92521800
H	-1.90229500	0.20264200	6.93677700
H	-0.11553300	-0.02566500	6.72997300
H	-4.69287400	-5.49801800	-3.03231000
H	-0.53750200	-3.62337100	-5.10665200
H	-0.44786000	1.55164900	-6.44038800
H	4.31814200	1.37700100	5.26385100
H	2.80429200	2.96965700	7.21275500
H	2.52400900	3.50760900	8.87633000
H	2.43666700	6.39607300	4.73047700

The enzyme-substrate complex with a monodentate binding mode of the substrate to the Mn-enzyme of 2,3-DHBD_*Ao* (+1.7)

C	4.54677100	3.57240900	5.53678000
C	4.66082500	2.34770700	4.61834100
C	3.98222600	2.61347100	3.26263200
C	3.93183000	1.41684700	2.31703600
O	4.95387700	0.69694300	2.18860200
O	2.85793900	1.19499100	1.66167900
H	4.78415300	4.48888300	4.98392400
H	5.70806800	2.08954900	4.44418900
H	4.19448800	1.47692600	5.09641700
H	2.96786100	3.00175200	3.41395100
H	4.55661500	3.39631500	2.74774800
C	-9.55383000	5.03580600	-0.91276700
C	-8.20440200	4.37990800	-0.82239500
C	-7.59383000	3.92450200	0.32247600
C	-7.22721100	4.23211100	-1.87694000
C	-6.04173600	3.69629600	-1.28977300
C	-7.22655900	4.52321100	-3.25184500
N	-6.30668300	3.49439700	0.04788500
C	-4.87436400	3.47143500	-2.02854000
C	-6.07173300	4.29640400	-3.99221700
C	-4.90726100	3.78113500	-3.38510300
H	-9.51717300	5.87631200	-1.61478300
H	-9.83692000	5.44854900	0.06117200
H	-7.97339400	3.89282800	1.33334500
H	-5.64146100	3.21030200	0.75304900
H	-8.11637700	4.92491000	-3.72793200
H	-3.97453500	3.07817300	-1.56528200
H	-6.06151800	4.52195000	-5.05424000
H	-4.01682700	3.62007300	-3.98510200
C	-8.11199500	-0.84718300	0.24332400
C	-7.65916100	-0.75844500	-1.18865400
C	-7.43720900	-1.91814600	-1.94238400
C	-7.41006300	0.48708400	-1.77653600
C	-6.98124600	-1.82767400	-3.25611300
C	-6.93437700	0.58051000	-3.08583200
C	-6.71752300	-0.57999300	-3.82887200
H	-8.35660300	0.14077100	0.64427600
H	-7.33554200	-1.28083900	0.88273100
H	-7.61944100	-2.89223200	-1.49434900
H	-7.58809200	1.39302100	-1.20541200
H	-6.83499500	-2.73402600	-3.83609100
H	-6.74126100	1.55742700	-3.51645700
H	-6.34792300	-0.51607900	-4.84770700
C	8.86860300	6.53056500	0.75781200
C	8.51318400	5.14691900	1.25001300
C	9.17321400	4.59236000	2.35428800
C	7.49384300	4.38619600	0.66120500
C	8.82013500	3.35002800	2.87054700
C	7.12612700	3.13551000	1.16142200
C	7.77977900	2.61519700	2.29048500
O	7.44962000	1.43041000	2.86279100
H	9.46825000	7.06093000	1.50371900
H	7.96680800	7.12948100	0.58349800
H	9.97172800	5.15535300	2.83149500
H	6.97635900	4.77499600	-0.21410800
H	9.32745300	2.93425700	3.73471200
H	6.35262100	2.54998800	0.67384500
H	6.56280800	1.13732300	2.55157200
C	4.66860300	4.30879900	-2.71237800
C	3.75133000	4.16221600	-3.90845100
O	3.61981900	5.07186600	-4.73046900
C	4.03495800	5.29006000	-1.68526800
C	2.83694600	4.72409300	-1.02322200
C	2.67428100	4.15897100	0.21343500
N	1.64915200	4.47332300	-1.69066900
C	0.84040600	3.74841800	-0.88199700
N	1.43393100	3.55618100	0.29195100
H	4.94631800	3.36265300	-2.24474100
H	4.77233500	5.52849200	-0.91400800
H	3.79912200	6.21867100	-2.21650800
H	1.46900300	4.69847400	-2.65919600
H	3.37796100	4.12928600	1.02896700
H	-0.13744100	3.37669000	-1.14960700
N	2.99267200	3.03610800	-3.96453500
C	2.13713700	2.79635500	-5.13148500
C	1.73232400	1.32542600	-4.98874400
C	1.83177200	1.04807900	-3.48040000
C	3.04734600	1.87592300	-3.05343300
H	2.71176600	2.98725400	-6.04357500
H	0.74000800	1.12992700	-5.38861400
H	2.44775500	0.69248900	-5.52374900
H	0.92845000	1.39158200	-2.96454400
H	1.96603600	-0.01153300	-3.25019500
H	2.98751300	2.19704100	-2.01035500
H	3.98346300	1.32345900	-3.18164800
C	-3.91243000	-2.66867200	-5.23531600
C	-2.87597000	-3.67545200	-4.81231200
O	-1.95215600	-4.03864000	-5.54442200
H	-3.45397500	-1.67896500	-5.23503400
H	-4.77663600	-2.63243600	-4.57730700
N	-2.99217400	-4.13587900	-3.53804900
C	-1.95341000	-4.99373500	-2.98485700
C	-2.44560100	-5.28664300	-1.56580800
C	-3.22409000	-4.02499200	-1.20283700
C	-3.93068600	-3.66177700	-2.50651100
H	-0.98679500	-4.47505600	-2.99214500
H	-3.11934100	-6.14727200	-1.57798200
H	-1.63868200	-5.51057700	-0.86605000
H	-3.91588900	-4.15620300	-0.37306800
H	-2.52382000	-3.22919500	-0.94226300
H	-4.89741100	-4.17656700	-2.58961900
H	-4.11180800	-2.58951600	-2.58752100
C	0.36126900	-1.73163500	-5.34695100
C	-0.79697900	-0.90533300	-4.85170700
C	-1.23108900	-1.05609700	-3.52553500
C	-1.47780000	0.00755800	-5.66576200
C	-2.31488100	-0.33134400	-3.03405300
C	-2.55097800	0.75635700	-5.17003900
C	-2.97922200	0.58594800	-3.85314200
H	0.13579600	-2.78726000	-5.15562300
H	1.28764700	-1.48030300	-4.81957400
H	-0.72502000	-1.76712600	-2.87672800
H	-1.16908900	0.12582600	-6.70098400
H	-2.65243900	-0.48391600	-2.01700500

H	-3.06580900	1.45571300	-5.82293200
H	-3.83101800	1.13799000	-3.47107900
C	10.00910000	0.57480100	-1.54839600
C	9.35400600	-0.18057000	-0.40480800
O	9.99993300	-0.80884500	0.42718000
H	9.32959500	1.31443100	-1.98444800
N	7.99251000	-0.11063200	-0.39602000
C	7.24479100	-0.94432800	0.49935500
C	7.02273700	-2.36244100	-0.03826600
O	7.27241800	-2.70035000	-1.19415900
H	7.49019400	0.27812500	-1.19257900
H	7.76097800	-0.98716400	1.46154900
H	6.26254800	-0.49282100	0.66699000
N	6.45799900	-3.20373000	0.86981600
C	5.89786900	-4.48170200	0.46629900
C	4.81483600	-4.34893100	-0.63206500
C	3.89431900	-3.21522400	-0.29311600
C	3.15162000	-2.98523100	0.83716400
N	3.80011400	-2.05874400	-1.05228400
C	3.04866600	-1.16583200	-0.40698100
N	2.63839600	-1.71239000	0.74004000
H	6.12541700	-2.80775700	1.74739100
H	5.44472100	-4.91225600	1.36582600
H	5.27768300	-4.16374200	-1.60042100
H	4.25827400	-5.28990400	-0.70880800
H	4.25730000	-1.85590700	-2.02727400
H	2.98379100	-3.59082300	1.70987500
H	2.82917600	-0.16197000	-0.72828100
C	7.42112800	-4.83459000	-4.86767900
C	7.27699100	-3.41466700	-5.42171500
C	6.80277800	-2.37031800	-4.40177100
C	5.39002000	-2.50324300	-3.84868800
O	4.76364000	-3.58831400	-3.91875900
O	4.92166400	-1.45661200	-3.25798400
H	6.43983400	-5.17045700	-4.52120800
H	8.23892800	-3.07171900	-5.82335600
H	6.57987700	-3.43237800	-6.26910600
H	7.44365300	-2.37378400	-3.50967300
H	6.88356200	-1.36313300	-4.82816600
C	4.73701700	-3.04574400	5.00869000
C	4.61566300	-1.69559000	4.31624400
O	4.93673800	-1.87004400	2.94555300
H	4.39481400	-2.98817100	6.04699800
H	3.59282000	-1.30235500	4.41260800
H	5.28843000	-0.96494700	4.78784500
H	4.90393000	-0.98270700	2.52485200
C	0.95382000	0.05834000	6.22757500
C	0.83123700	-0.96292400	5.10237700
C	0.78616600	-0.36372500	3.70093200
O	0.52711800	0.86636000	3.55556000
O	1.01014500	-1.16358100	2.74714700
H	1.27341300	1.01310400	5.79809700
H	1.65337300	-1.68384900	5.10535800
H	-0.07961000	-1.55817200	5.20526100
C	-4.04569100	-0.41681200	4.12844400
C	-4.24534000	1.01346600	3.69614200
C	-5.53697300	1.55394800	3.65143400
C	-3.16752800	1.83566200	3.34960700
C	-5.74802900	2.88359000	3.28814400
C	-3.37970200	3.16208800	2.97010300
C	-4.66753100	3.70129200	2.94169500
H	-2.98426800	-0.66018400	4.23407000
H	-4.47507400	-1.11420100	3.40685000
H	-6.38456500	0.92769200	3.91884200
H	-2.16051600	1.42414400	3.36988900
H	-6.75638000	3.28766300	3.29051700
H	-2.54745100	3.79395300	2.69510700
H	-4.81611000	4.74104700	2.66013300
C	-3.06706900	-3.15968400	7.76608600
C	-1.68438700	-3.69741200	7.40392300
C	-1.39838600	-3.71065500	5.88977700

C	-2.03118300	-4.73462400	4.97327100
O	-3.32917600	-4.97458200	5.16230400
O	-1.40644300	-5.25085200	4.05286900
H	-3.83582500	-3.91498900	7.57714400
H	-0.91229100	-3.05699600	7.84531600
H	-1.52950700	-4.69720600	7.82458800
H	-1.70622500	-2.74098900	5.47312800
H	-0.32371800	-3.80443300	5.71967000
C	-2.45319100	0.30293200	0.32236400
C	-2.04559000	-1.00022600	0.67040900
C	-3.00042400	-1.92395400	1.12849200
C	-4.35307600	-1.62238900	1.00219200
C	-4.75843200	-0.35724000	0.56357600
C	-3.81776900	0.60899100	0.26886600
O	-0.75820300	-1.42371000	0.58865100
O	-1.84310300	2.54573600	-0.14346100
O	-0.21475400	1.05575300	0.30739200
Mn	0.96653600	1.95559700	1.75876000
O	0.08451600	3.55441400	2.96459100
O	-0.08547700	-3.51030100	2.47580100
O	-1.33748800	4.88846000	1.08990800
O	6.23228700	0.78959800	-2.56437300
C	0.94784600	-8.51731900	-0.42850700
C	2.07113900	-7.49893300	-0.70304800
N	1.60596600	-6.19785700	-1.20483500
C	1.02372600	-5.25618000	-0.44069700
N	0.76289700	-4.05461000	-0.96810800
N	0.67402700	-5.51433800	0.82316500
H	0.27716700	-8.55966600	-1.29197500
H	0.33565700	-8.24609700	0.43715600
H	2.68645300	-7.34740500	0.19424000
H	2.73926100	-7.89118900	-1.47208300
H	1.93589100	-5.85506300	-2.11136000
H	1.25661200	-3.80963600	-1.83597000
H	0.33114100	-3.33428500	-0.40068500
H	0.84730200	-6.42124800	1.21918600
H	0.39453200	-4.73724000	1.44601300
C	-4.58000800	-8.46169100	-0.39146900
C	-4.53916200	-7.20616900	0.44521100
C	-3.52915800	-6.73435900	1.23762300
C	-5.68369800	-6.35023900	0.69981700
C	-5.28706000	-5.39746500	1.67134700
C	-6.98828900	-6.30148300	0.18629000
N	-3.96349800	-5.66166900	2.03589600
C	-6.15754700	-4.41709000	2.14874200
C	-7.85136500	-5.30547900	0.63559700
C	-7.44240200	-4.37550400	1.61017800
H	-3.58326500	-8.86022600	-0.59428200
H	-5.10877000	-8.31384000	-1.33680200
H	-2.50762300	-7.07709100	1.32531900
H	-3.34506300	-4.85803100	2.15248500
H	-7.31919200	-7.02456400	-0.55342200
H	-5.84661700	-3.72114800	2.92220600
H	-8.86174000	-5.25165400	0.24200300
H	-8.14260800	-3.62210600	1.95620200
O	2.31122000	-4.25081700	-3.24773100
H	-5.80388700	-0.11395600	0.48724000
H	-5.08176300	-2.37851900	1.26779400
H	-4.10762900	1.61301300	-0.00015600
C	-1.46470000	1.38312900	0.12495500
O	-2.59928700	-3.11263000	1.66400500
H	-1.62292000	-3.12226700	1.83593000
H	-0.58345700	5.24367600	0.59896200
H	-1.56266600	4.04685500	0.61399600
H	-0.44977600	3.22657800	3.69955100
H	-0.46743300	4.16627000	2.41384000
C	-2.76759200	8.38353800	1.68440600
C	-2.65552100	8.48376000	0.18317600
O	-2.71765400	9.53969100	-0.44051500
C	-4.24110400	8.24413500	2.14001900
C	-5.13070600	9.39622700	1.68211600

O	-4.77959000	6.97868900	1.74008100
H	-2.24897100	7.48378600	2.03630900
H	-4.23985700	8.19966900	3.23434000
H	-4.81895500	6.96378900	0.77328200
H	-6.15295100	9.23973400	2.03666900
H	-4.76259500	10.35057500	2.07086100
H	-5.13929000	9.47595700	0.59071100
N	-2.58245700	7.26311600	-0.42255400
C	-2.66533200	7.14037700	-1.86540400
C	-2.01774200	5.84727200	-2.34742200
H	-2.51459700	6.42724100	0.14805200
H	-2.17257100	8.01487800	-2.29879500
H	-2.12589000	5.74573100	-3.43061400
H	-0.94883000	5.84363000	-2.10553500
H	-2.48147400	4.97037000	-1.88692000
H	-0.23021200	-0.58896800	0.47854400
H	2.08087900	-1.24596400	1.47475400
H	0.37835500	-2.65008600	2.68506100
H	-0.30265300	-4.01933300	3.27907800
H	5.83398100	-0.08042200	-2.82953700
H	6.72352900	1.07560700	-3.34404000
H	3.24939200	-3.92894400	-3.44522200
H	1.84854900	-4.20854200	-4.09456900
H	-2.28801400	9.23279500	2.18223700
H	-3.71644100	7.19792300	-2.16701600
H	-10.38718600	4.40011100	-1.22988000
H	-8.98385500	-1.49463500	0.38423000
H	-5.13474000	-9.17925900	0.22237100
H	-4.27452500	-2.85240400	-6.25230200
H	-1.82961000	-5.93198300	-3.53544600
H	1.32642500	-9.53035500	-0.25607700
H	7.74050700	-5.54634500	-5.63610300
H	8.07923900	-4.88699000	-3.99394600
H	6.70744300	-5.15774900	0.17232800
H	4.12713600	-3.78264100	4.47562500
H	5.79236300	-3.33799200	5.00943500
H	10.90155200	1.09935800	-1.19087800
H	10.26282200	-0.14530200	-2.33324100
H	9.43796500	6.55184600	-0.17730600
H	5.23279300	3.53918500	6.38971400
H	3.52921700	3.67649200	5.92738300
H	1.71666200	-0.19491400	6.97131700
H	-0.01584800	0.26215000	6.69312800
H	5.58058800	4.84471700	-2.99561400
H	1.28730500	3.48466800	-5.18700900
H	0.55778800	-1.66392000	-6.42214300
H	-4.54341600	-0.57884300	5.09015100
H	-3.32216200	-2.30947700	7.12482100
H	-3.17329600	-2.83881600	8.80786300
H	-3.63068300	-5.53473200	4.41427300

References

[1] R. P. Joosten, F. Long, G. N. Murshudov, A. Perrakis, *IUCrJ* **2014**, *1*, 213-220.

[2] G. Hofer, S. Wieser, M. K. Bogdos, P. Gattinger, R. Nakamura, M. Ebisawa, M. Mäkelä, N. Papadopoulos, R. Valenta, W. Keller, *Allergy* **2019**, *74*, 1009–1013.

[3] A. Burkhardt, T. Pakendorf, B. Reime, J. Meyer, P. Fischer, N. Stübe, S. Panneerselvam, O. Lorbeer, K. Stachnik, M. Warmer, P. Rödig, D. Göries, A. Meents, *Eur. Phys. J. Plus* **2016**, *131*, 56.

[4] D. Liebschner, P. V. Afonine, M. L. Baker, G. Bunkóczi, V. B. Chen, T. I. Croll, B. Hintze, L. W. Hung, S. Jain, A. J. McCoy, N. W. Moriarty, R. D. Oeffner, B. K. Poon, M. G. Prisant, R. J. Read, J. S. Richardson, D. C. Richardson, M. D. Sammito, O. V. Sobolev, D. H. Stockwell, T. C. Terwilliger, A. G. Urzhumtsev, L. L. Videau, C. J. Williams, P. D. Adams, *Acta Cryst. D* **2019**, *75*, 861-877.

[5] P. Emsley, B. Lohkamp, W. G. Scott, K. Cowtan, *Acta Cryst. D* **2010**, *66*, 486–501.

[6] X. Sheng, K. Plasch, S. E. Payer, C. Ertl, G. Hofer, W. Keller, S. Braeuer, W. Goessler, S. M. Glueck, F. Himo, K. Faber, *Front. Chem.* **2018**, *6*, 608.

[7] https://web.expasy.org/protparam

[8] S. Xu, W. Li, J. Zhu, R. Wang, Z. Li, G.-L. Xu, J. Ding, *Cell Res*. **2013**, *23*, 1296-1309.

[9] A. Vladimirova, Y. Patskovsky, A. A. Fedorov, J. B. Bonanno, E. V. Fedorov, R. Toro, B. Hillerich, R. D. Seidel, N. G. J. Richards, S. C. Almo, F. M. Raushel, *J. Am. Chem. Soc*. **2016**, *138*, 826-836.

[10] M. Yoshida, N. Fukuhara, T. Oikawa, *J. Bacteriol.* **2004**, 186, 6855-6863.

[11] X. Sheng, Y. Patskovsky, A. Vladimirova, J. Bonanno, S. C. Almo, F. Himo, F. M. Raushel, *Biochemistry* **2018**, *57*, 3167-3175.

[12] K. Kirimura, H. Gunji, R. Wakayama, T. Hattori, Y. Ishii, *Biochem. Biophys. Res. Commun.* **2010**, *394*, 279-284.

[13] X. Zhang, J, Ren, P. Yao, R. Gong, M. Wang, Q. Wu, D. Zhu, *Enz. Microb. Technol.* **2018**, *113*, 37–43.

[14] R. Santha, N. A. Rao, C. S. Vaidyanathan, *Biochim. Biophys. Acta Prot. Struct. Mol. Enzymol.* **1996**, *1293*, 191–200.

[15] a) A. D. Becke, *J. Chem. Phys.* **1993**, *98*, 5648–5652; b) C. Lee, W. Yang, R. G. Parr, *Phys. Rev. B* **1988**, *37*, 785–789; c)S. Grimme, J. Antony, S. Ehrlich, H. Krieg, *J. Chem. Phys.* 2010, *132*, 154104; d) S. Grimme, S. Ehrlich, L. Goerigk, *J. Comput. Chem.* **2011**, *32*, 1456–1465.

[16] M. J. Frisch, G. W. Trucks, H. B. Schlegel, G. E. Scuseria, M. A. Robb, J. R. Cheeseman, G. Scalmani, V. Barone, B. Mennucci, G. A. Petersson, H. Nakatsuji, M. Caricato, X. Li, H. P. Hratchian, A. F. Izmaylov, J. Bloino, G. Zheng, J. L. Sonnenberg, M. Hada, M. Ehara, K. Toyota, R. Fukuda, J. Hasegawa, M. Ishida, T. Nakajima, Y. Honda, O. Kitao, H. Nakai, T. Vreven, J. A. Montgomery Jr., J. E. Peralta, F. Ogliaro, M. Bearpark, J. J. Heyd, E. Brothers, K. N. Kudin, V. V. Staroverov, T. Keith, R. Kobayashi, J. Normand, K. Raghavachari, A. Rendell, J. C. Burant, S. S. Iyengar, J. Tomasi, M. Cossi, N. Rega, J. M. Millam, M. Klene, J. E. Knox, J. B. Cross, V. Bakken, C. Adamo, J. Jaramillo, R. Gomperts, R. E. Stratmann, O. Yazyev, A. J. Austin, R. Cammi, C. Pomelli, J. W. Ochterski, R. L. Martin, K. Morokuma, V. G. Zakrzewski, G. A. Voth, P. Salvador, J. J. Dannenberg, S. Dapprich, A. D. Daniels, O. Farkas, J. B. Foresman, J. V. Ortiz, J. Cioslowski, D. J. Fox, Gaussian 09, Revision D.01; Gaussian, Inc., Wallingford, CT, **2013**.

[17] P. J. Hay, W. R. Wadt, *J. Chem. Phys.* **1985**, *82*, 270–283.

[18] A. V. Marenich, C. J. Cramer, D. G. Truhlar, *J. Phys. Chem. B* **2009**, *113*, 6378–6396.

[19] a) F. Himo, *J. Am. Chem. Soc.* **2017**, *139*, 6780–6786; b) X. Sheng, M. Kazemi, F. Planas, F. Himo, *ACS Catal.* **2020**, *10*, 6430−6449.
